# Biomimetic syntheses of kadcoccitane H and kadcotrione C methyl ester†‡

**DOI:** 10.1039/d5sc00669d

**Published:** 2025-03-03

**Authors:** Dattatraya H. Dethe, Salman A. Siddiqui, Chirantan Singha

**Affiliations:** a Department of Chemistry, Indian Institute of Technology Kanpur Kanpur – 208016 India ddethe@iitk.ac.in

## Abstract

Kadcotriones and kadcoccitanes, renowned for their intricate 6/6/5-tricyclic and 6/6/5/6-tetracyclic ring systems, respectively, exhibit promising biological activities. This work proposes a biosynthetic pathway that elucidates how nature synthesizes these triterpenoids from lanosterol. Inspired by this pathway, we present the first biomimetic syntheses of kadcoccitane H and kadcotrione C methyl ester. These syntheses showcase key transformations including olefin transposition, a biomimetic ring contraction/expansion, SeO_2_ mediated one-pot allylic oxidation/isomerization–elimination/allylic oxidation cascade, regioselective dihydroxylation of sterically hindered double bond, unusual POCl_3_ mediated cleavage of diol and Still–Gennari olefination.

## Abstract 2

The first biomimetic syntheses of kadcoccitane H and kadcotrione C methyl ester were achieved by following our biosynthetic proposal for lanosterol-to-kadcoccitanes.

## Graphical abstract

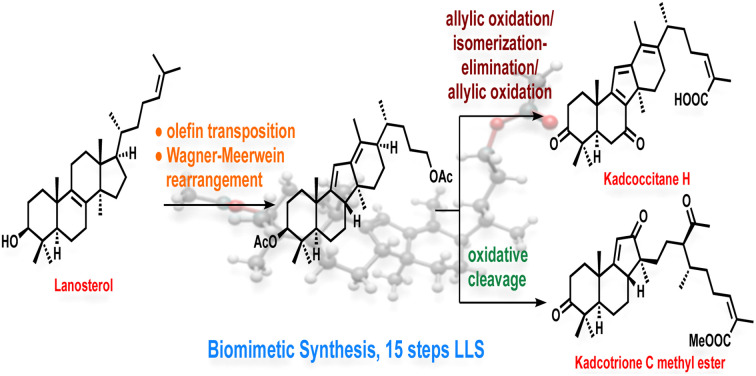

Plants of the family Schisandraceae, consisting of the genera *Schisandra* and *Kadsura*, are a rich source of triterpenoids with novel complex polycyclic structures and diverse biological activities.^1^*Kadsura coccinea*, an evergreen climbing shrub widely distributed in the southwestern provinces of China, is one of the few intensively studied *Kadsura* species. This is partly because it has long been used as traditional Chinese medicine for its beneficial pharmacological effects, such as gastropathy, rheumatic arthritis, anticancer, antihepatitis, and anti-HIV-1 activities. Furthermore, it has been proven to be a rich source of novel kadcotriones and kadcoccitanes which are 14(13 → 12)-*abeo*-12,13-*seco*-lanostane and 14(13 → 12)-*abeo*-lanostane triterpenoids, respectively, possessing rearranged lanostane frameworks with interesting structural features of daedal oxygenation and cleavage patterns.

In 2013, Sun and co-workers isolated kadcotriones A–C (1–3, Fig. 1)^2^ and subsequently in 2015 the same group reported the isolation of kadcoccinic acids A–J.^3^ Later in 2019, Puno and co-workers isolated kadcoccitanes A–D^4^ and recently in 2023, they reported the isolation of kadcoccitanes E–H (4–6, Fig. 1)^5^ from the stem of *Kadsura coccinea.* Kadcotriones possess 6/6/6-fused tricyclic (for kadcotrione A) and 6/6/5-fused tricyclic (for kadcotriones B–C) ring skeletons with a unique keto–acid side chain. In addition to that, kadcoccitanes E–H have a unique 6/6/5/6-fused tetracyclic ring skeleton featuring α,β,γ,δ-unsaturated aldehyde or α,β,γ,δ,ε,ζ-unsaturated aldehyde/ketone and an α,β-unsaturated acid side chain with *Z*-olefin geometry. Kadcoccitanes and kadcotriones contain three quaternary carbons and four to six stereocenters.

**Fig. 1:**
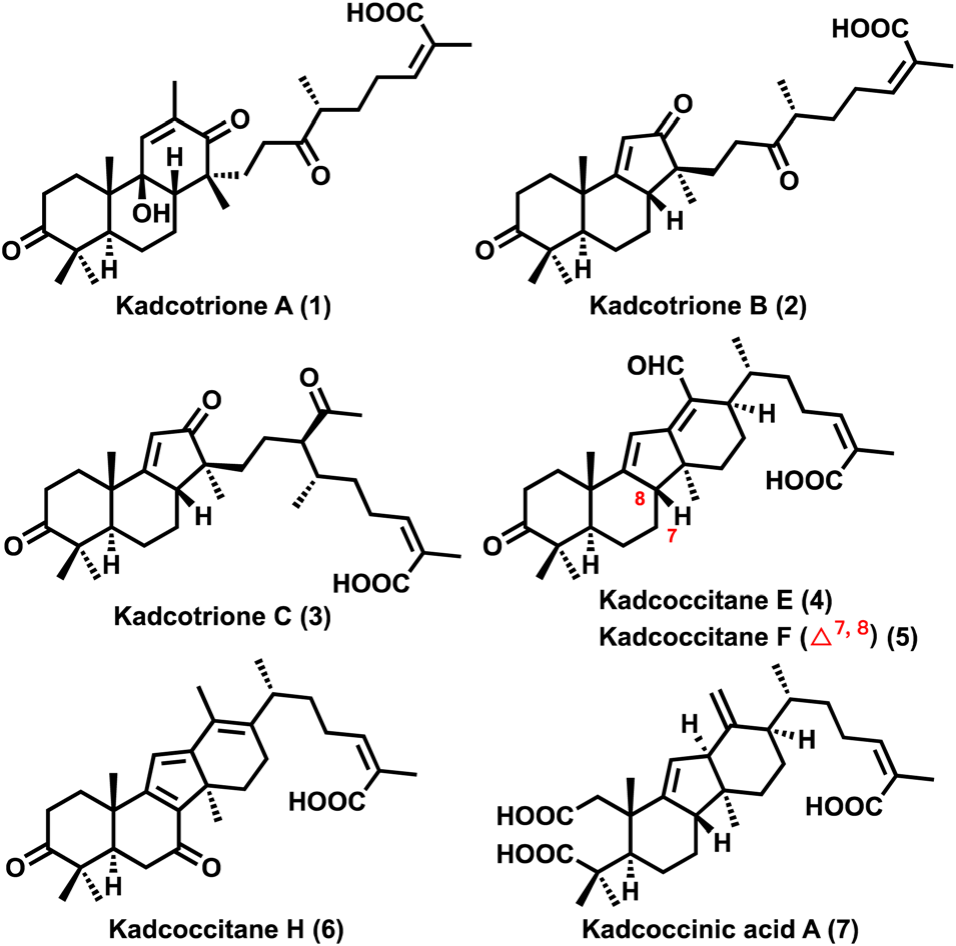
Kadcotriones A–C and kadcoccitanes E–H.

Herein, we report expeditious syntheses of kadcoccitane H and kadcotrione C methyl ester starting from commercially available lanosterol and making use of biomimetic and diastereoselective transformations. Prior to our synthesis, Trost's group^6^ reported an elegant synthesis of the trimethyl ester of kadcoccinic acid A (7) in 23 LLS, starting from 2-methyl-1,3-cyclohexadione. Our synthesis, which entails an oxidative olefin transposition, Wagner–Meerwein type rearrangement, dihydroxylation/diol cleavage, and finally a Still–Gennari olefination reaction, enables the rapid construction of these unusual natural products. This strategy also provides access to various kadcoccitane analogues that do not exist in nature which might be useful in investigating the biological activities of these unique natural products. In recent years, rearranged steroids have garnered significant attention from synthetic chemists, driven by their diverse structural motifs and intriguing biological activities. A significant subgroup of rearranged steroids, known as *abeo*-steroids, are often produced naturally from classical steroids.^7*a*–*d*^

Our synthetic strategy for kadcotrione C (3) was primarily guided by a biosynthetic pathway proposed by Sun and co-workers (Scheme 1A).^2^ They suggested that diene intermediate 9 might be derived from 12β-hydroxycoccinic acid 8 by means of a carbocation intermediate followed by oxidation of 9 which would afford kacotrione B & C. This proposed biosynthetic pathway motivated us to design a practical and efficient route to synthesize these rearranged lanostane frameworks since the carbocation intermediate 8a is likely to undergo a C13–C14 bond migration from the C13 to C12 position to afford 8b (Scheme 1A).^7*e*,*f*^ Further, we have proposed and experimentally validated a combined biosynthetic analysis (Scheme 1B) for kadcotrione C and kadcoccitane H, highlighting a 6/6/5/6-fused tetracyclic ring system, likely originating from lanosterol (10). It was envisioned that regioselective olefin transposition would occur from C8–C9 to C9–C11 positions, followed by enzymatic oxidation to form 12β-hydroxycoccinic acid 8. The alcohol group in 8 could facilitate a Wagner–Meerwein type rearrangement, leading to the formation of diene 9*via* carbocation intermediate 8b. Thereafter, further oxidation of 9 could yield kadcoccitane H (6). On the other hand, chemoselective oxidative cleavage of the C12–C13 double bond in 10 would generate kadcotrione C (3).

**Scheme 1:**
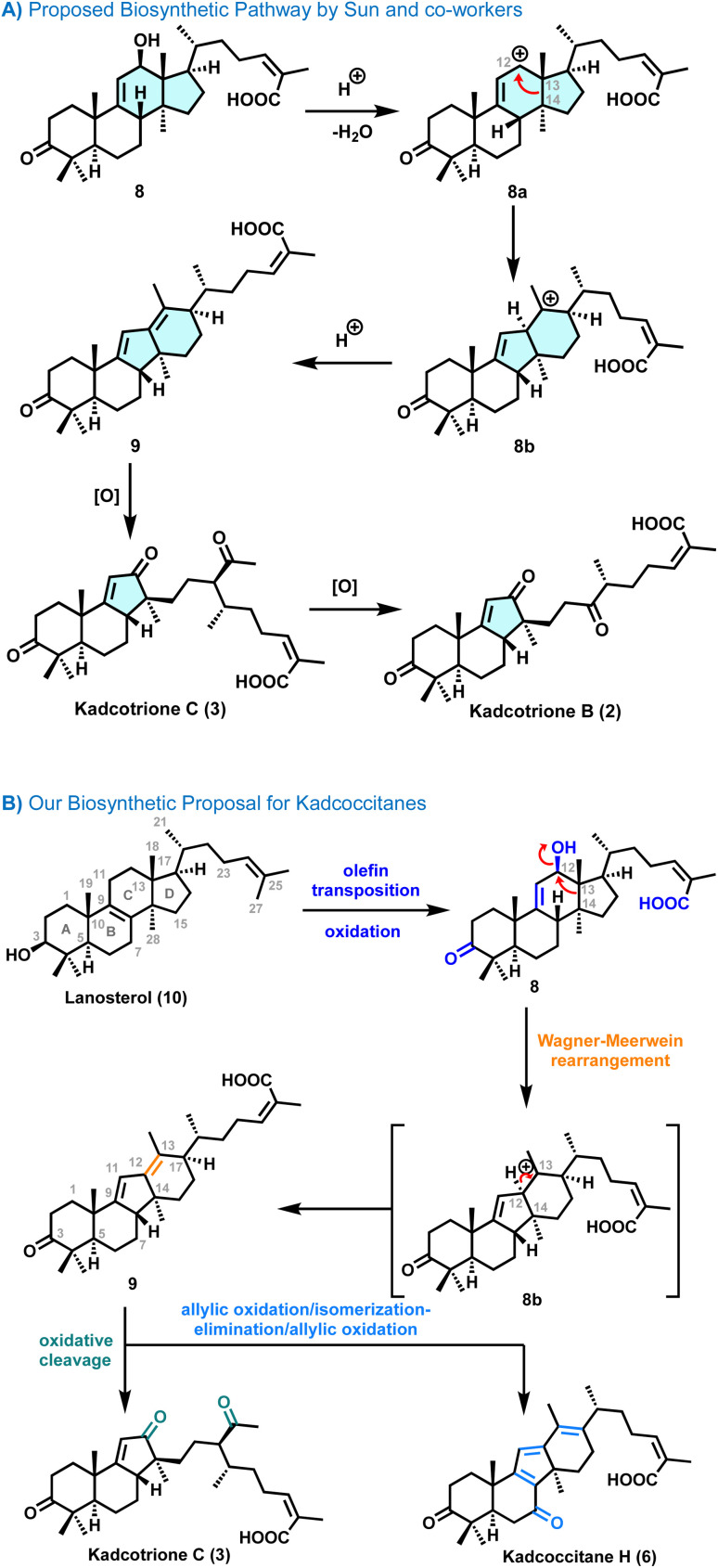


Although the total synthesis of the kadcoccinic acid A trimethyl ester was reported by Trost's group from the (+)-Wieland–Miescher ketone derivative, we chose a semisynthetic route to this C-*nor*-D-*homo* steroid substructure for the sake of brevity and efficiency. We presumed that the cationic rearrangement of 12β-hydroxy steroids into their C-*nor*-d-*homo* counterparts^8^ was a much easier approach. Since 12β-hydroxy steroids are rare and properly functionalized, one resembling the kadcoccitane ABCD skeleton was not available, so we envisioned the hydroxylation of commercially available steroids (*e.g.* lanosterol) at the C12 position by olefin transposition followed by allylic oxidation.

To experimentally validate the biosynthetic proposal, our synthesis commenced with the known acid^9^11 that was prepared from technical grade lanosterol (8) (*ca.* 50% pure) in two steps and 47% overall yield (Scheme 2). Acid 11 was converted to the corresponding methyl ester using K_2_CO_3_/MeI. Next, we focused on installing an olefin at the C9–C11 position by transposing the C8–C9 olefin, which could be used as a handle for the allylic oxidation to obtain kadcoccitane H. To our delight, exposure of the methyl ester to conditions described by Staliński and Paryzek^10^ (CrO_2_Cl_2_, CH_2_Cl_2_, −30 °C) led to Δ^9(11)^-7-oxo 12 as the major product (60%, 95% brsm), with the desired configuration at C8. The high regio- and stereoselectivity of this transformation could be attributed to the specific scaffold of the Δ^8(9)^-lanostane substrate. Direct deoxygenation at C7 in 12 was unsuccessful; therefore, the 7-oxo derivative 12 was transformed into the corresponding xanthate through reduction with NaBH_4_, followed by treatment of the resultant alcohol with CS_2_ and MeI in the presence of DBU. Subsequent Barton–McCombie deoxygenation (AIBN/^*n*^Bu_3_SnH) provided 13 (overall 75% in three steps).

**Scheme 2:**
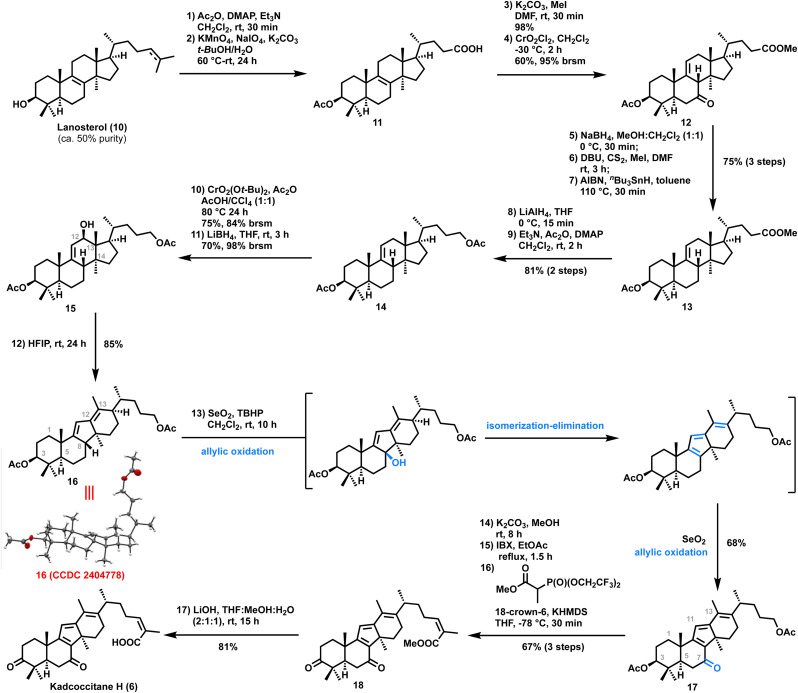
Synthesis of kadcoccitane H.

With the well-defined tetracyclic core 13 in hand, we turned our attention to the key Wagner–Meerwein rearrangement. We initially attempted to introduce a hydroxyl group at the C12 position by allylic oxidation of 13 followed by ketone reduction (see the ESI‡). However, upon exposure to various Wagner–Meerwein rearrangement conditions, the alcohol with the –CO_2_Me side chain yielded a complex reaction mixture. To circumvent this issue, we converted the side chain –CO_2_Me group to an acetate. Thus, reduction of 13 with lithium aluminum hydride followed by acetylation of the corresponding diol afforded diacetate 14 in 81% yield over two steps. Allylic oxidation of diacetate 14 using freshly prepared di-*tert*-butyl chromate led to the formation of an α,β-unsaturated ketone in 75% (84% brsm) yield.^11^ Reduction of the C12 ketone using sodium borohydride afforded the corresponding alcohol 15 with poor yield (only 9%) probably due to the sterically hindered nature of the ketone. Exposure to Luche conditions (sodium borohydride/cerium(iii) chloride heptahydrate) also generated similar results. Fortunately, lithium borohydride reduction led to the formation of the corresponding alcohol 15 in 70% (98% brsm) yield. With this alcohol in hand, the stage was set to perform the Wagner–Meerwein rearrangement. Initially, alcohol 15 was exposed to different Lewis/Brønsted acids (*e.g.*.BF_3_·OEt_2_, Cu(OTf)_2_, *p*-TSA *etc.*, see the ESI‡) which led to the formation of a complex reaction mixture. Also, alcohol 15 on treatment with trifluoromethanesulfonic anhydride/pyridine or methane sulfonyl chloride/triethyl amine generated a complex reaction mixture. To our delight, alcohol 15 on reaction with 1,1,1,3,3,3-hexafluoroisopropanol (HFIP) at room temperature for 24 hours afforded the desired tetracyclic diene 16 in 85% yield. The structure of diene 16 was unambiguously established by single crystal X-ray crystallographic analysis (CCDC 2404778).

With the key tetracyclic diene 16 in hand, we embarked on the completion of the targets kadcoccitane E/H and kadcotrione C methyl ester. It was envisioned that selective allylic oxidation of the C13 methyl group would generate the fully functionalized core of kadcoccitane E; on the other hand, allylic oxidation of diene at the tertiary position (C8) would generate an allylic *tert*-alcohol which could be transformed to kadcoccitane H on further functional group manipulation. To our surprise, treatment of diene 16 with SeO_2_/TBHP (for detailed screening of conditions, see the ESI‡) directly generated α,β,γ,δ,ε,ζ-unsaturated ketone 17 in 68% yield by a one-pot allylic oxidation/isomerization–elimination/allylic oxidation cascade (Scheme 2).^12^ Next, to install the *Z*-olefinic acid side chain,^13^ diacetate in 17 was first hydrolyzed using K_2_CO_3_/MeOH to the corresponding diol. IBX mediated oxidation of the diol followed by Still–Gennari olefination of the resultant keto–aldehyde yielded the desired *Z*-olefinic methyl ester 18 in 67% yield over three steps. Gratifyingly, hydrolysis of the methyl ester of 18 using lithium hydroxide afforded kadcoccitane H (6) in 81% yield. All analytical data (^1^H NMR, ^13^C NMR, HRMS, IR, and [*α*]_D_^20^) of the synthesized 6 matched with the isolation report of the natural product.

On the other hand, synthesis of kadcotrione C methyl ester 22 was achieved by selective cleavage of the C12–C13 double bond of diene 16. Thus, regioselective dihydroxylation of C12–C13 olefin 19 using NaIO_4_ or Pb(OAc)_4_ was unsuccessful. Interestingly, while trying dehydration of one of the hydroxy groups in diol 19 using POCl_3_/pyridine, we observed diol cleavage to afford the enone 20 in 80% yield which might be occurring *via* a 5-membered phosphorus(v) intermediate^14^ which eventually got cleaved, providing the tricyclic core skeleton of kadcotrione C. Although the reaction of a diol with POCl_3_ to generate a 5-membered phosphorus(v) intermediate is reported in the literature,^14^ its cleavage to form a diketone has been observed for the first time. Next, the acetate groups of 20 were hydrolyzed. Thus, the treatment of 20 with K_2_CO_3_/MeOH resulted in a 1 : 1 diastereomeric mixture, presumably due to epimerization of the acetyl group on the side chain of 20. To address this issue, we employed dibutyltin oxide in MeOH, a mild *trans*-esterification reagent. Treatment of 20 with excess dibutyltin oxide followed by IBX mediated oxidation of the resultant diol provided the aldehyde. Still–Gennari olefination of the aldehyde afforded the desired *Z*-olefinic methyl ester of kadcotrione C 21 in 48% yield in three steps (Scheme 3).

**Scheme 3:**
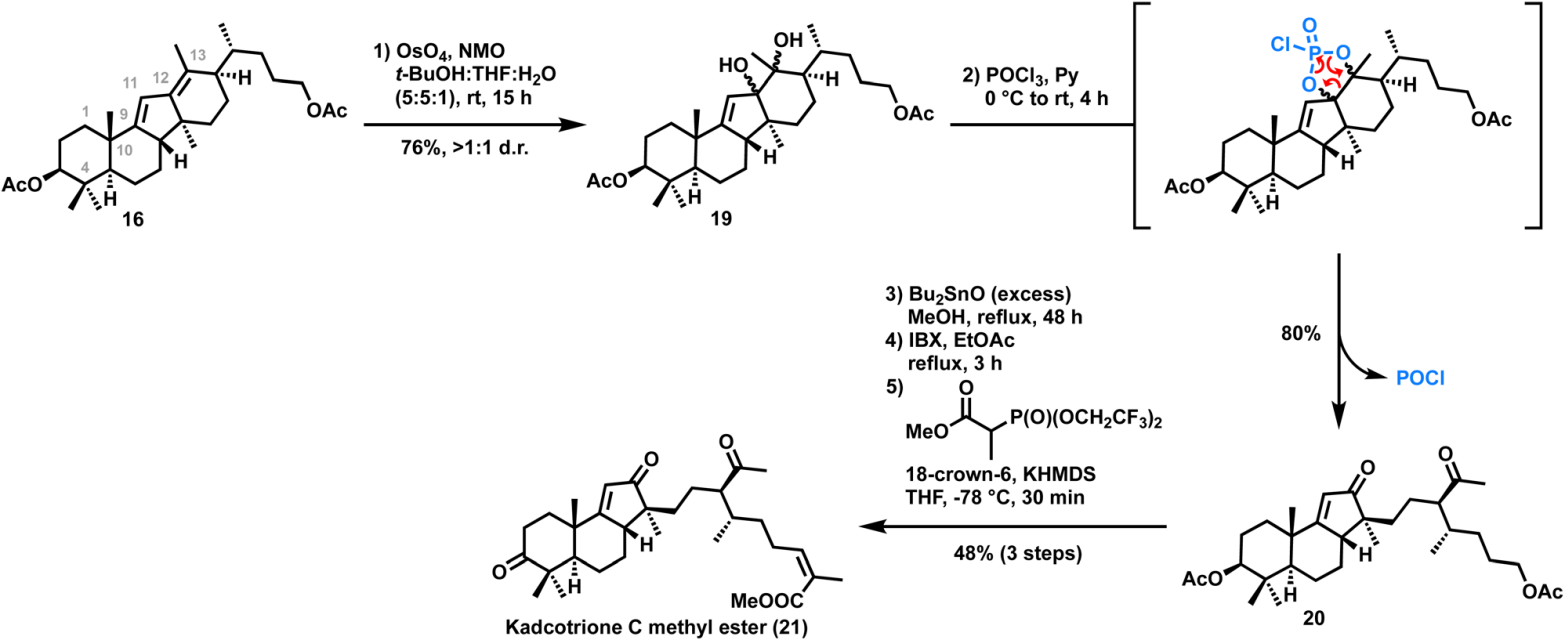
Synthesis of kadcotrione C methyl ester.

## Conclusion

In conclusion, we have developed a concise and efficient strategy for the syntheses of kadcoccitane H (17 steps, 6.8% overall yield) and kadcotrione C methyl ester (15 steps, 5.4% overall yield). These syntheses feature a regioselective olefin transposition, a biomimetic ring contraction/expansion, SeO_2_ mediated one-pot allylic oxidation/isomerization–elimination/allylic oxidation cascade, chemoselective dihydroxylation of sterically hindered double bond, unusual POCl_3_ mediated cleavage of diol and Still–Gennari olefination reaction. Our biomimetic approach highlights the power of steroidal skeletal rearrangements in efficiently synthesizing complex and biologically active natural products. Furthermore, additional manipulations of tetracyclic diene 16 could enable the synthesis of a broad range of natural and unnatural derivatives of kadcoccitanes. Work in this area is currently underway and will be reported in due course.

## Data availability

The data supporting this article have been included as part of the ESI.‡ Crystallographic data for compound 16 have been deposited at the CCDC under 2404778 and can be obtained from https://www.ccdc.cam.ac.uk/data_request/cif.

## Author contributions

D. H. D. directed the project and wrote the manuscript. S. A. S. and C. S. prepared the ESI‡ and wrote the manuscript.

## Conflicts of interest

The authors declare no competing financial interest.

## Supplementary Material

SC-OLF-D5SC00669D-s001

SC-OLF-D5SC00669D-s002
