# Supplementary material for: Biomimetic syntheses of kadcoccitane H and kadcotrione C methyl ester
Source: Chem Sci. 2025 Mar 3;16(14):6099–103. doi: 10.1039/d5sc00669d (PMC11894464; doi:10.1039/d5sc00669d)

Supplementary Information (SI) for Chemical Science.
This journal is © The Royal Society of Chemistry 2025

Supporting Information

Biomimetic Syntheses of Kadcoccitane H and Kadcotrione C Methyl Ester

Dattatraya H. Dethe*, Salman A. Siddiqui and Chirantan Singha

Department of Chemistry, Indian Institute of Technology Kanpur, Kanpur-208016, India

Tel: + 91-512-2596537, fax: + 91-512-2597436.

Table of Contents: -

1) **General Aspects…………………………….………………....S3**

2) **Initial Approaches…….……………………………….………...S4**

3) **Final Scheme……………..…...……………………………...…S12**

4) **Experimental Procedures and Characterisation Data…...…..S13**

5) **NMR data comparison tables ……………………...…………S27**

6) **X-Ray Crystallographic Data……………….….……………...…S28**

7) **References………………………………..………..……………S29**

8) **^{1}H & ^{13}C NMR data……………………...……………………….S30**

1) General Aspects: -

Experiments with moisture and air-sensitive components were conducted using oven dried glassware. Unless otherwise noted, all reactions were carried out under an argon atmosphere with dry solvents under anhydrous conditions. Tetrahydrofuran (THF) and toluene were distilled immediately before use from sodium and benzophenone. Dichloromethane (CH_2Cl_2 or DCM), Dimethylformamide (DMF) were distilled from calcium hydride and stored under an argon atmosphere. Ethyl acetate and hexane were distilled from calcium chloride. All other solvents were used in HPLC grade directly for reactions. Commercial reagents were used without further purification unless otherwise noted.

Reactions were monitored by thin layer chromatography (TLC) carried out on Merck TLC Silica Gel 60 F_{254}-aluminium plates and visualised under UV-light or staining with a *p*-anisaldehyde – sulfuric acid solution and heating using a heat gun. Column chromatography was performed with CDH Silica Gel 100 – 200 Mesh. Prep. TLC was performed on 20x20 cm Merck Silica Gel 60 F_{254}-aluminium plates. Yields refer to chromatographically pure compounds unless otherwise stated.

NMR was recorded on either a JEOL ECS-400 (1H, 400 MHz; 13C, 100 MHz), or JEOL JNM-ECZ500R (1H, 500 MHz; 13C, 125 MHz). The spectra were calibrated using residual un-deuterated solvents as internal references for 1H NMR and deuterated solvents for ^{13}C NMR. Specifically, chloroform (δH = 7.26 ppm) was used for ^{1}H NMR and $CDCl_3$ (δC = 77.16 ppm) for ^{13}C NMR. The following abbreviations were used to explain the multiplicities: s = singlet, d = doublet, t = triplet, q = quartet, m = multiplet, br = broad and combinations.

Mass spectrometric data were obtained using Agilent 6546 LC/Q-TOF instrument.

IR data recorded from PerkinElmer, FT-IR spectrometer, Spectrum Two.

Optical rotations were measured using a Polarimeter (Anton Paar MCP 150) at 20 °C, different cell lengths used were 10 mm and 50 mm in $CHCl_3$ and concentration in g/100 mL. Melting points were measured on a capillary melting point apparatus.

2) Initial approaches: -

1st approach

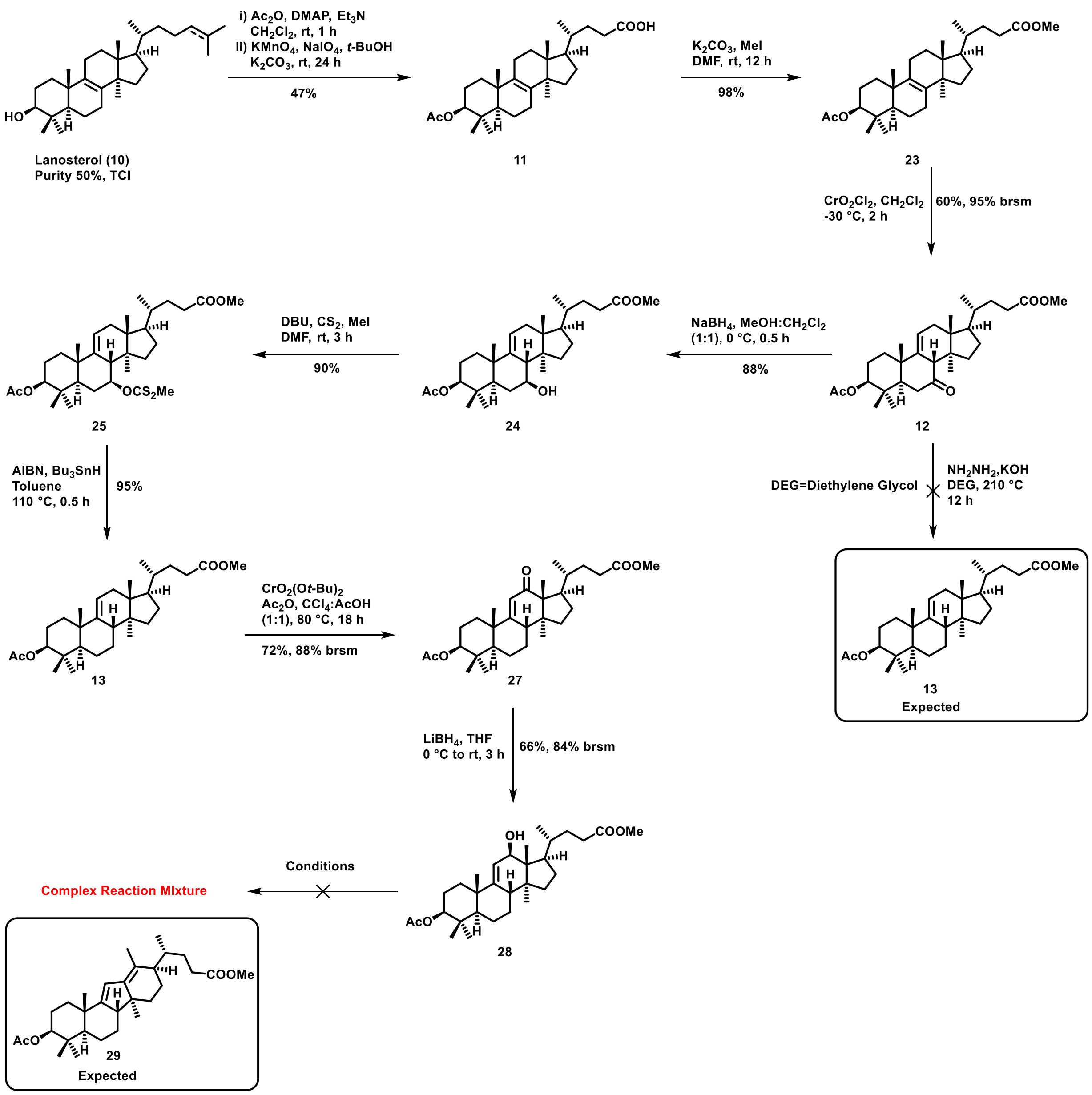

Table 1: -

Standardization of Allylic Oxidation

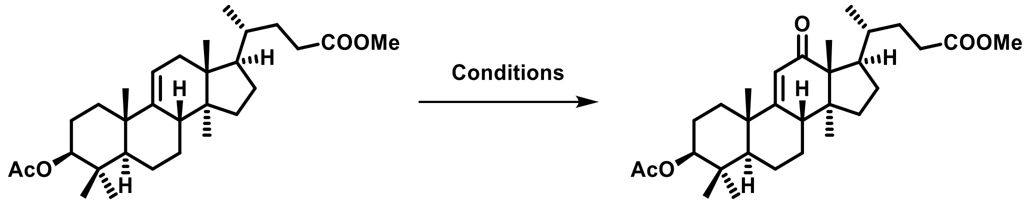

13 27

Entry	Reagent	Solvent	Temperature	Time	Yield
1.	CrO_3 (20 eq.) 3,5-DMP(20 eq.)	CH_2Cl_2	-20 °C to rt	24 h	No Reaction
2.	$RuCl_3.3H_2O$ (1 to 5 mol%) TBHP (3.5 to 5 eq)	Cyclohexane	rt	24 h	No Reaction
3.	$Mn(OAc)_3.2H_2O$ (10 - 50 mol%) TBHP (5 eq.)	EtOAc	rt	24 h	9%, 90% brsm
4.	$CrO_2(O^tBu)_2$ (5.0 eq.) Ac_2O (10 eq.)	$AcOH/CCl_4$ (1:1)	80 °C	24 h	72%, 88% brsm

Table 2: -

Standardization of Enone Reduction

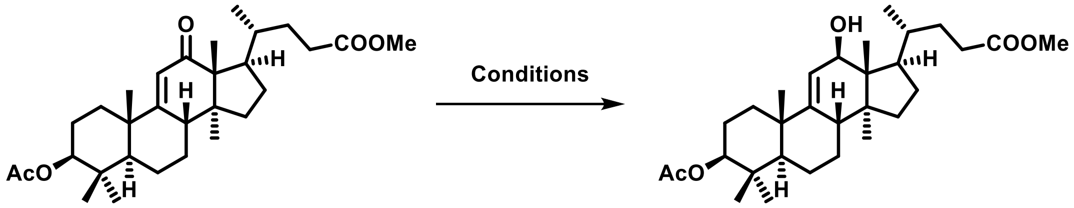

27 28

Entry	Reagent	Solvent	Temperature	Time	Product
1.	$NaBH_4$ (1.5 eq.) $CeCl_3.7H_2O$ (0.5 eq.)	$MeOH:CH_2Cl_2$ (3:1)	0 °C to rt	5 h	6%, 75% brsm
2.	$NaBH_4$ (1.5 eq.)	$MeOH:CH_2Cl_2$ (1:1)	0 °C to rt	2 h	9%, 88% brsm
3.	$LiBH_4$ (3 eq.)	THF	0 °C to rt	3 h	66%, 84% brsm

Table 3: -

Standardization of Wagner-Meerwein Rearrangement on -COOMe side chain

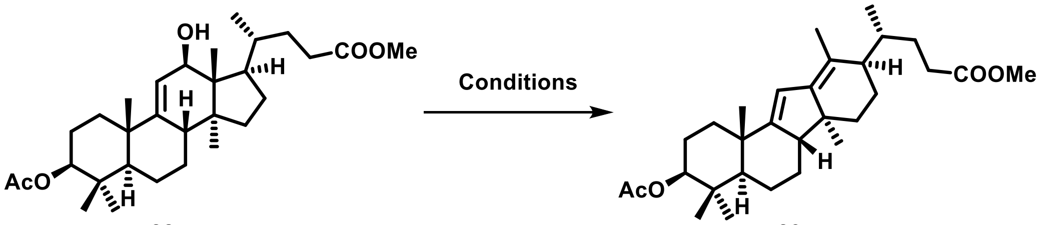

**Entry**	**Reagent**	**Solvent**	**Temperature**	**Time**	**Product**
1.	BF_3•OEt_2 (1 to 2.5 eq)	CH_2Cl_2	0 °C	10 min	Complex Reaction Mixture
2.	$Cu(OTf)_2$ (0.2 eq.)	CH_2Cl_2	rt	12 h	Complex Reaction Mixture
3.	*p*-TSA (0.2 eq.)	CH_2Cl_2	0 °C	10 min	Complex Reaction Mixture
4.	Tf_2O (1.0 eq.)	Pyridine	0 °C to 70 °C	5 h	Complex Reaction Mixture
5.	MsCl (1.0 eq.)	Pyridine	0 °C to rt	20 min	Complex Reaction Mixture
6.	HFIP	-	rt	12 h	Complex Reaction Mixture

2nd Approach: -

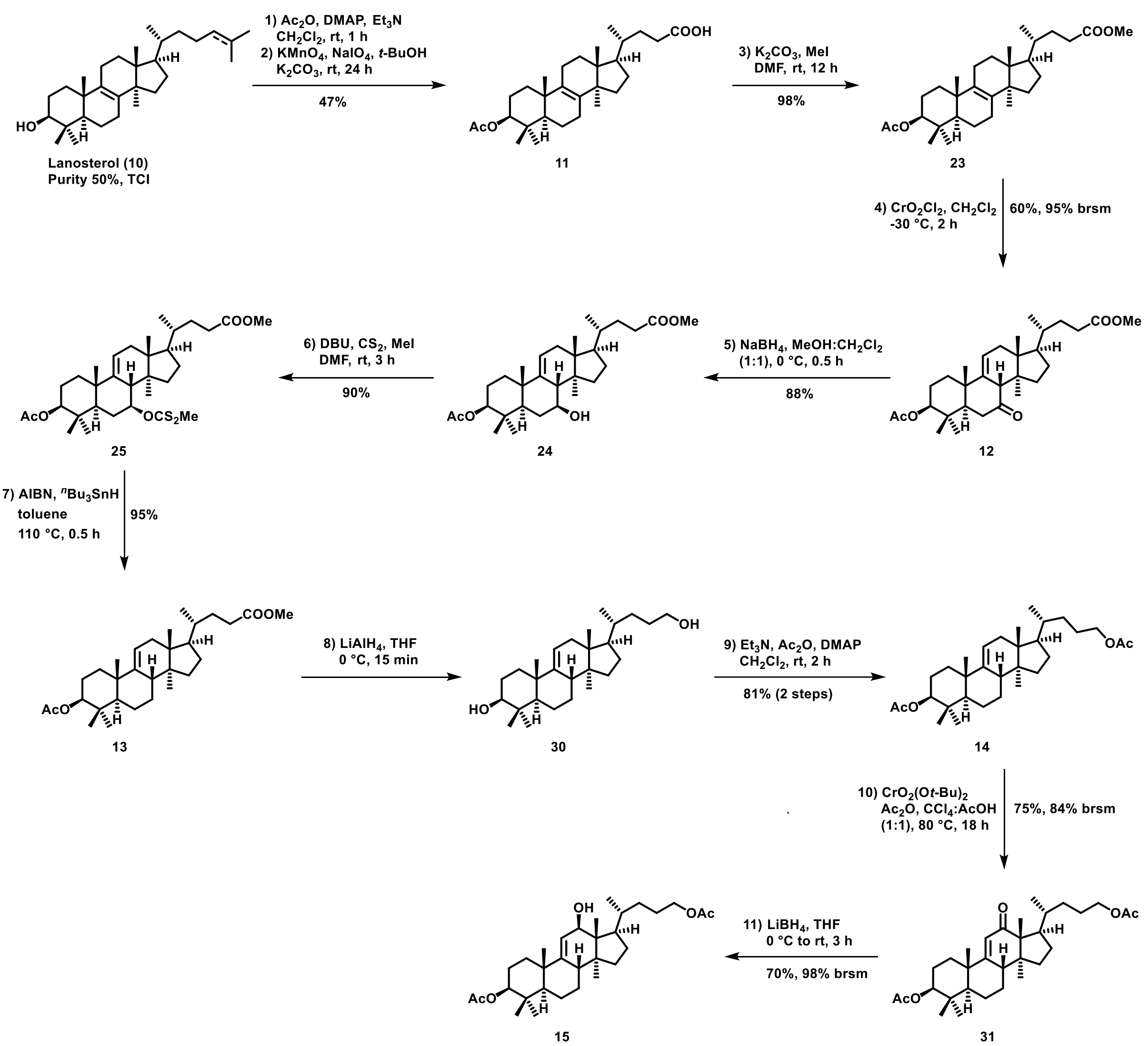

Table 4: -

Standardization of Wagner-Meerwein Rearrangement on -OAc side chain

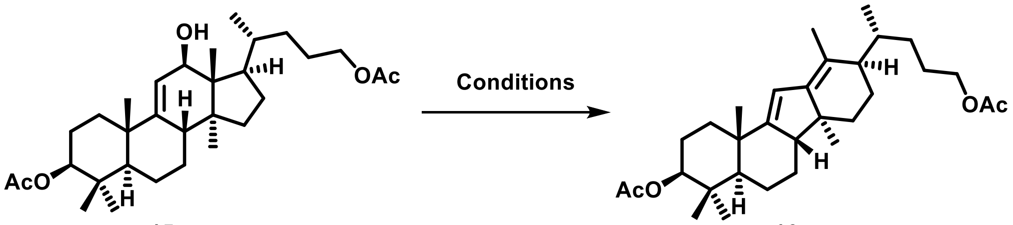

15 16

Entry	Reagent	Solvent	Temperature	Time	Product
1.	BF_3•OEt_2 (1 to 2.5 eq)	CH_2Cl_2	0 °C	10 min	Complex Reaction Mixture
2.	$Cu(OTf)_2$ (0.2 eq.)	CH_2Cl_2	rt	15 h	Complex Reaction Mixture
3.	*p*-TSA (0.2 eq.)	CH_2Cl_2	0 °C	10 min	Complex Reaction Mixture
4.	Tf_2O (1.0 eq.)	Pyridine	0 °C to 70 °C	5 h	Complex Reaction Mixture
5.	MsCl (1.0 eq.)	Pyridine	0 °C to rt	30 min	Complex Reaction Mixture
6.	HFIP	-	rt	24 h	85%

Table 5: -

Standardization of allylic oxidation/isomerization-elimination/allylic oxidation cascade

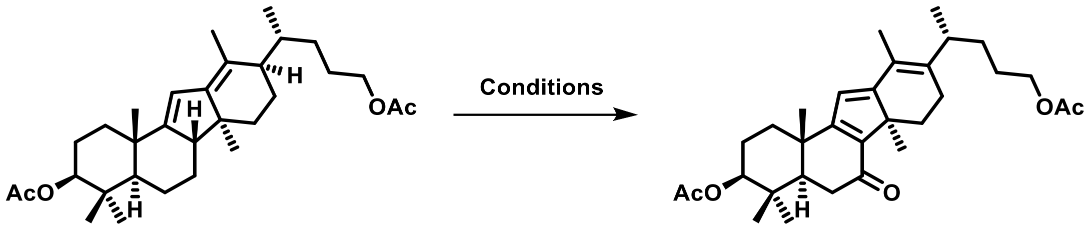

Entry	Reagent	Solvent	Temperature	Time	Product
1.	SeO_2 (4 eq.)	Dioxane: H_2O (1:1)	50 °C	16 h	Complex Reaction Mixture
2.	SeO_2 (4 eq.)	Dioxane	rt	24 h	Complex Reaction Mixture
3.	SeO_2 (2 eq.)	CH_2Cl_2	rt	12 h	SM decomposed
4.	CrO_2Cl_2 (1 eq.)	CH_2Cl_2	-30 °C	2 h	SM decomposed
5.	CrO_3 (20 eq.) 3,5-DMP (20 eq.)	CH_2Cl_2	-20 °C to rt	24 h	No Reaction
6.	$Cr_2(Ot\text{-}Bu)_2$ (2eq.) Ac_2O (10 eq.)	CCl_4: AcOH(1:1)	80 °C	12 h	Complex Reaction Mixture
7.	SeO_2 (1 eq.), AcOH (1.5 eq.)	Dioxane	rt	12 h	55%
8.	SeO_2 (1 eq.), TBHP (5 eq.)	CH_2Cl_2	rt	12 h	68%

Table 6: -

Failed Attempts to cleave C12-C13 Double Bond

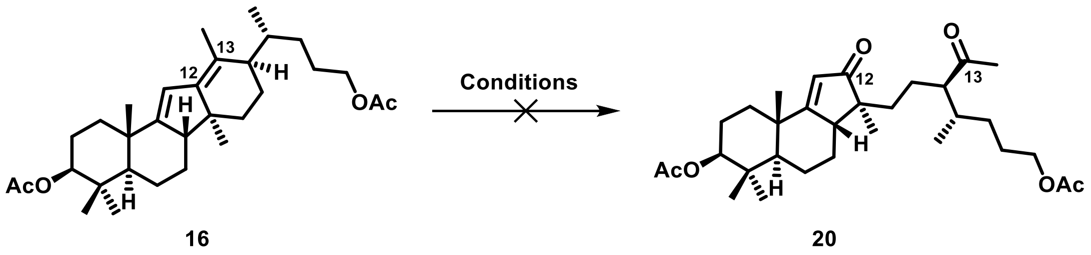

Entry	Reagent	Solvent	Temperature	Time	Product
1.	O_3, Me_2S (5 eq.)	CH_2Cl_2	-78 °C	1 min	S.M. Decomposed
2.	OsO_4 (0.1 eq.) , 2,6-lutidine (2 eq.) $NaIO_4$ (4 eq.)	1,4-Dioxane:H_2O (3:1)	rt	24 h	No Reaction
3.	$RuCl_3.3H_2O$ (0.5 eq.) $NaIO_4$ (2 eq.)	CH_3CN:H_2O (6:1)	0 °C	5 h	No Reaction

Table 7: -

Standardization of Oxidative Cleavage

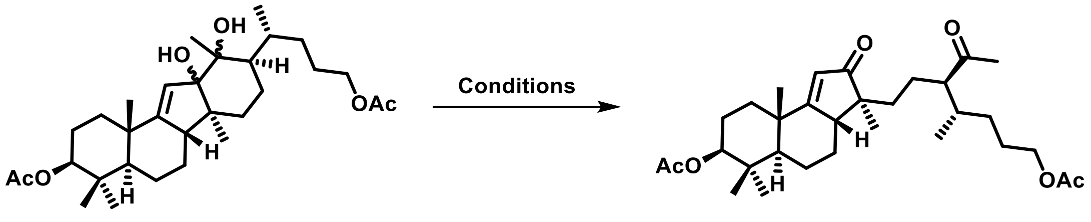

Entry	Reagent	Solvent	Temperature	Time	Product
1.	$Pb(OAc)_4$ (1.5-5eq.)	CH_2Cl_2/ Acetone (3:1)	0 °C	24 h	No Reaction
2.	$NaIO_4$ (0.5 eq.), $HIO_4.2H_2O$ (1.1 eq.)	$THF:H_2O$ (1:1)	rt	12 h	No Reaction
3.	$RuCl_3.3H_2O$(3.5 mol%), $NaIO_4$ (2 eq.)	$CH_3CN:H_2O$ (6:1)	0 °C	5 h	No Reaction
4.	Burgess Reagent (1 eq.)	Toluene	70 °C	12 h	45%
5.	Tf_2O (3 eq.)	Pyridine	0 °C to rt	1 h	30%
6.	$POCl_3$ (1 eq.)	Pyridine	0 °C to rt	4 h	80%

3) Final Scheme: -

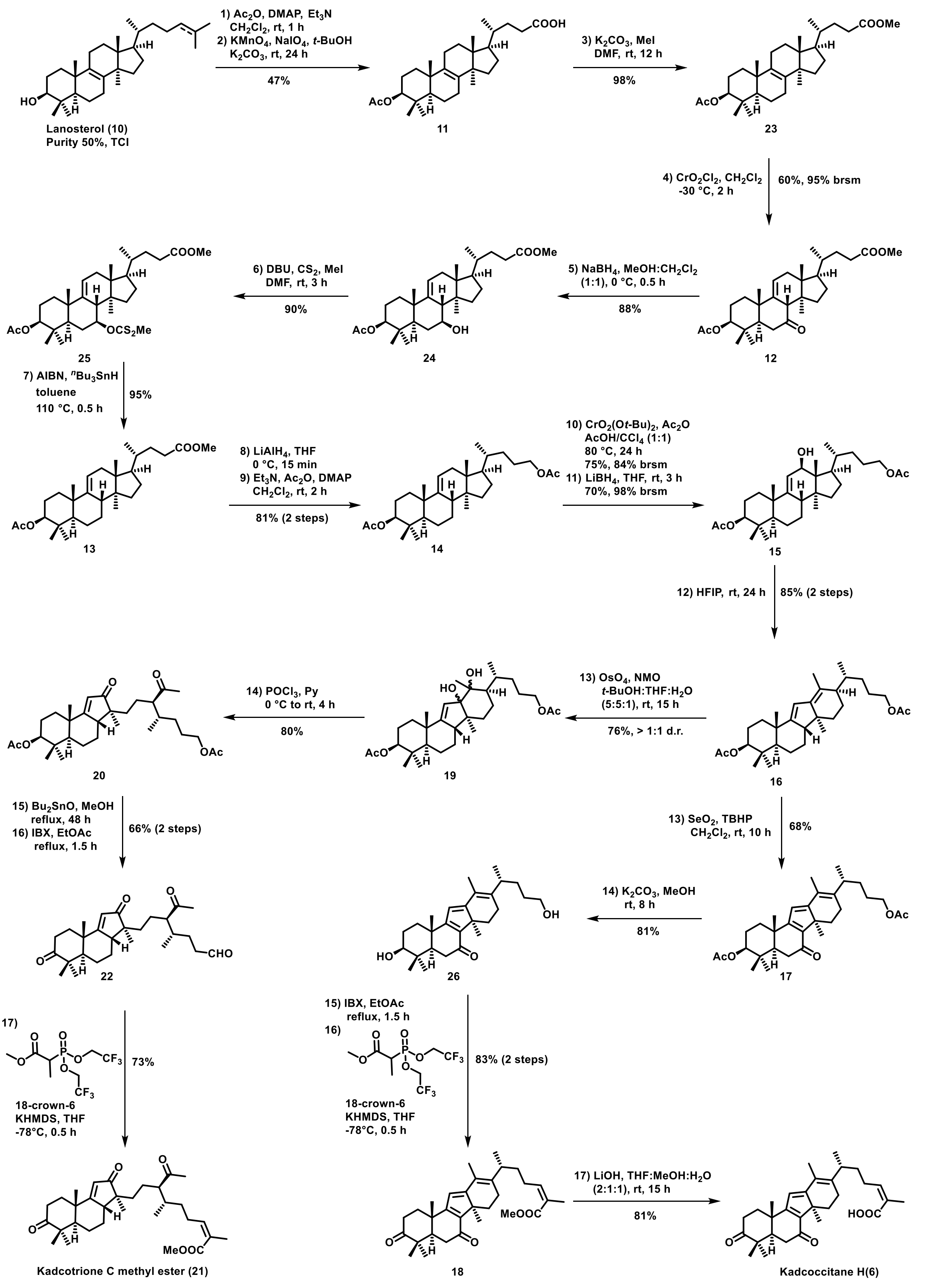

4) Experimental Methods and Characterisation Data: -

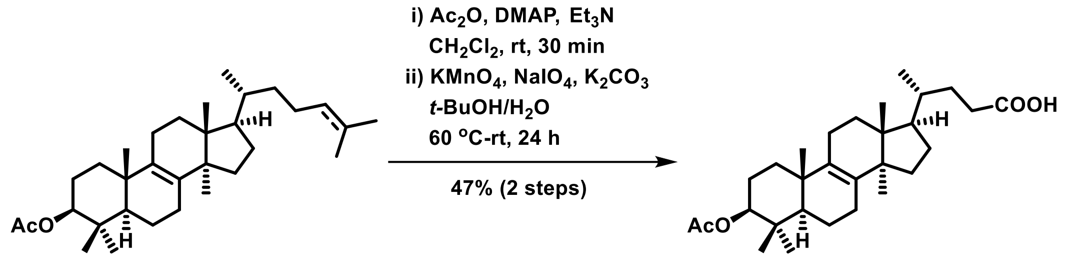

lanosterol (10)
(purity 50%, TCI)

11

To a solution of lanosterol **(10)** (10 gm, 23.47 mmol, 50% purity) in dry CH_2Cl_2 (50 mL) were added DMAP (287 mg, 2.35 mmol), Et_3N (17 mL, 118 mmol), and Ac_2O (4.5 mL, 46.94 mmol). The mixture was stirred at room temperature for 30 min. On complete conversion, all volatile parts were removed in rotary evaporator to afford the corresponding crude acetate as a yellowish solid.

To the stirred solution of the above yellowish solid in *t*-BuOH (500 mL), K_2CO_3 (9.72 g, 70.41 mmol) followed by an aqueous solution (300 mL) of $NaIO_4$ (25.12 g, 117.35 mmol) and $KMnO_4$ (1.85 g, 11.74 mmol) were added sequentially at 60 °C. It was stirred at 60 °C for 2 h then at rt for 24 h. Then to quench the reaction, 10% $NaHSO_3$ was added cautiously until the pale-yellow colouration comes. Then the *t*-BuOH was removed in rotary evaporator under reduced pressure. The aqueous phase was extracted with CH_2Cl_2 (3×100 mL) and dried over Na_2SO_4 and concentrated. At last, the residue was purified by column chromatography (silica gel, petroleum ether/EtOAc = 25:1 to 5:1, *v/v*) to afford acid **11** as a white crystal (5.40 g, 11.79 mmol, 47% over 2 steps).

TLC: R_f = 0.5 (petroleum ether/EtOAc = 4:1, *v/v*)

^{1}H NMR (400 MHz, $CDCl_3$): δ 4.50 (dd, *J* = 11.5, 4.6 Hz, 1H), 2.40 (ddt, *J* = 15.2, 10.3, 5.1 Hz, 2H), 2.30 – 2.18 (m, 1H), 2.05 (s, 3H), 2.03 – 1.91 (m, 4H), 1.87 – 1.80 (m, 1H), 1.66 (dddd, *J* = 27.5, 14.3, 7.9, 2.5 Hz, 8H), 1.48 (dd, *J* = 15.5, 7.5 Hz, 2H), 1.33 (ddd, *J* = 13.1, 8.4, 3.9 Hz, 4H), 1.19 – 1.12 (m, 2H), 1.00 (s, 3H), 0.91 (d, *J* = 5.8 Hz, 3H), 0.89 – 0.87 (m, 6H), 0.87 (s, 3H), 0.69 (s, 3H).

^{13}C NMR (100 MHz, $CDCl_3$): δ 179.7, 171.2, 134.6, 134.4, 81.1, 50.6, 50.4, 50.0, 44.7, 38.0, 37.1, 36.2, 35.4, 31.2, 31.2, 31.1, 30.9, 28.2, 28.1, 26.5, 24.4, 24.3, 21.5, 21.1, 19.3, 18.4, 18.3, 16.7, 15.9.

IR: ν_{max} = 3442, 2075, 1643, 1014, 747 cm^{-1}

HRMS (ESI-TOF): *m/z* for $[M+H]^+$ calcd. : 459.3474, found : 459.3649

m.p.: 183-184 °C

Opt. act.: $[\alpha]_D^{20}$= +193 (*c* = 0.72, $CHCl_3$)

All data are consistent with previous report.[9]

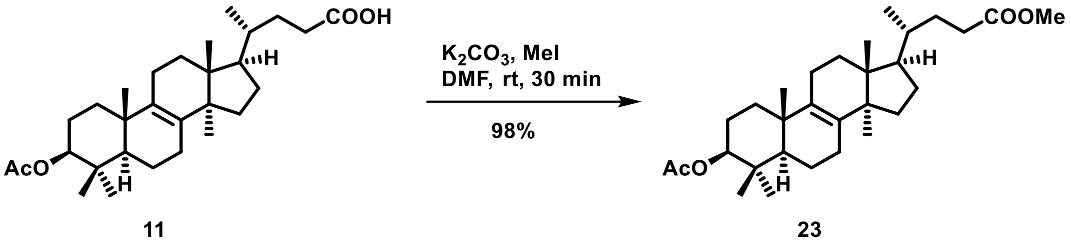

To a stirred solution of **11** (1 gm, 2.18 mmol) in dry DMF (0.5 mL), K_2CO_3 (692.06 mg, 5.01 mmol) was added at rt. After 5 min, MeI (0.68 mL, 10.9 mmol) was added dropwise and stirred for another 30 min and TLC was checked. On complete conversion reaction mixture was directly transferred to a short silica gel column and was purified to get **23** as white solid (1.01 g, 2.14 mmol, 98%).

TLC: R_f = 0.8 (petroleum ether/EtOAc = 4:1, *v/v*)

^{1}H NMR (400 MHz, $CDCl_3$): δ 4.49 (dd, J = 11.4, 4.6 Hz, 1H), 3.65 (s, 3H), 2.36 (ddd, J = 15.3, 10.0, 5.0 Hz, 1H), 2.22 (ddd, J = 18.8, 9.7, 6.6 Hz, 1H), 2.04 (d, J = 2.5 Hz, 4H), 2.02 – 1.88 (m, 3H), 1.85 – 1.77 (m, 1H), 1.75 – 1.70 (m, 2H), 1.66 (td, J = 6.1, 2.3 Hz, 3H), 1.63 – 1.52 (m, 3H), 1.51 – 1.45 (m, 1H), 1.45 – 1.37 (m, 2H), 1.37 – 1.23 (m, 3H), 1.22 – 1.10 (m, 2H), 0.99 (s, 3H), 0.91 – 0.82 (m, 12H), 0.68 (s, 3H).

^{13}C NMR (100 MHz, $CDCl_3$): δ 174.9, 171.1, 134.5, 134.4, 100.0, 81.0, 51.6, 50.6, 50.3, 49.9, 44.6, 37.9, 37.0, 36.2, 35.4, 31.4, 31.1, 30.9, 28.2, 28.0, 26.5, 24.3, 24.3, 21.4, 21.1, 19.3, 18.4, 18.2, 16.7, 15.9.

IR: ν_{max} = 2947, 2358, 2339, 1732, 1712, 1440, 1369, 1249, 1170, 1035, 773 cm^{-1}

HRMS (ESI-TOF): *m/z* for $[M+H]^+$ calcd.: 473.3631, found: 473.3613

m.p.: 154-156 ^oC

Opt. act.: $[\alpha]_D^{20}$= +149 (*c* = 0.61, $CHCl_3$)

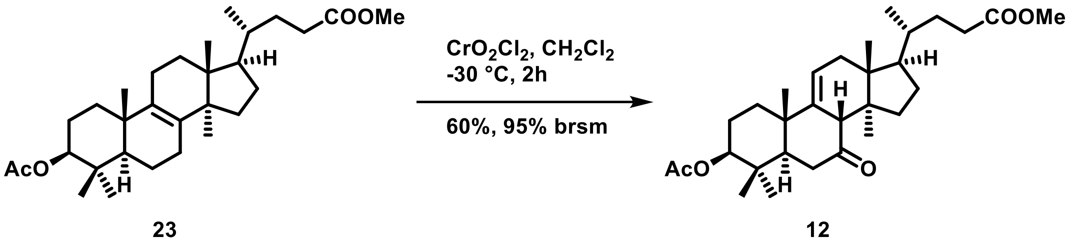

To a stirred solution of **23** (2.3 gm, 5 mmol) in CH_2Cl_2 (600 mL), CrO_2Cl_2 (0.34 mL) was added at -30 ^oC and stirred for 2 hrs. Then cooling bath was taken out and 10% Na_2SO_3 solution in H_2O (200 mL) followed by 5% NaOH solution in H_2O (200 mL) was added in 10 minutes. After that it was transferred to a separating funnel and organic layer was separated and aqueous layer was extracted with CH_2Cl_2 (3x50 mL) and dried over Na_2SO_4 then it was concentrated. At last, the residue was purified by column chromatography (silica gel, petroleum ether/EtOAc = 25:1 to 5:1, *v/v*) to afford olefin transposed product **12** as a white solid (1.46 g, 3 mmol, 60%, 95% brsm) and starting material **23** (868 mg, 1.84 mmol, 37%) was recovered.

TLC: R_f = 0.5 (petroleum ether/EtOAc = 4:1, *v/v*)

^{1}H NMR (400 MHz, $CDCl_3$): δ 5.39 (dt, J = 5.4, 2.6 Hz, 1H), 4.56 – 4.45 (m, 1H), 3.66 (s, 3H), 2.88 (d, J = 3.4 Hz, 1H), 2.43 – 2.37 (m, 2H), 2.37 – 2.32 (m, 1H), 2.23 (ddd, J = 15.7, 9.4, 6.7 Hz, 1H), 2.11 (dt, J = 18.4, 2.9 Hz, 1H), 2.06 (s, 3H), 1.98 – 1.71 (m, 8H), 1.51 – 1.40 (m, 3H), 1.37 – 1.23 (m, 3H), 1.11 (s, 3H), 0.92 (s, 3H), 0.89 (d, J = 6.1 Hz, 3H), 0.82 (s, 3H), 0.75 (s, 3H), 0.66 (s, 3H).

^{13}C NMR (100 MHz, $CDCl_3$): δ 212.4, 174.7, 170.9, 143.6, 117.8, 80.3, 56.5, 51.6, 49.7, 47.8, 47.3, 44.8, 39.0, 38.5, 38.1, 37.3, 35.8, 35.5, 34.5, 31.4, 31.3, 28.1, 27.4, 24.1, 21.4, 20.5, 18.1, 17.9, 15.8, 15.3.

IR: ν_{max} = 2951, 2358, 2339, 1737, 1373, 1240, 1029, 771 cm^{-1}

HRMS (ESI-TOF): *m/z* for $[M+H]^+$ calcd. : 487.3423, found : 487.3424

m.p.: 150-152 °C

Opt. act.: $[\alpha]_D^{20}$= +250 (*c* = 0.115, $CHCl_3$)

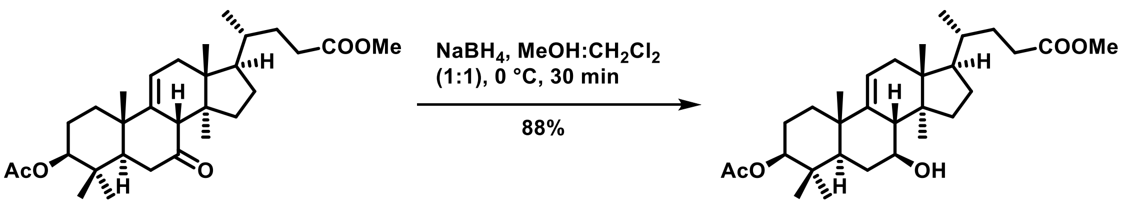

12 24

To a solution of ketone **12** (5 gm, 10.27 mmol) in dry CH_2Cl_2 (50 mL) and MeOH (50 mL) was added $NaBH_4$ (59.65 mmol) in portion wise at 0 °C and stirred for 30 min at the same temperature. After that TLC indicated that the starting material was consumed, then the reaction was quenched with sat. aq. NH_4Cl solution and the mixture was washed with brine and extracted with CH_2Cl_2 (3x30 mL), dried over Na_2SO_4, and concentrated. The residue was purified by flash column chromatography (petroleum ether/EtOAc = 2:1, *v/v*) to afford the corresponding alcohol **24** (4.41 gm, 9.03 mmol, 88%).

TLC: R_f = 0.3 (petroleum ether/EtOAc = 2:1, *v/v*)

^{1}H NMR (400 MHz, $CDCl_3$) δ 5.28 (d, *J* = 6.1 Hz, 1H), 4.45 (dd, *J* = 11.5, 4.1 Hz, 1H), 3.64 (s, 4H), 2.36 (ddd, *J* = 15.3, 10.0, 5.1 Hz, 1H), 2.22 (ddd, *J* = 15.5, 9.4, 6.5 Hz, 1H), 2.16 – 2.04 (m, 2H), 2.03 (s, 3H), 1.98 – 1.86 (m, 3H), 1.77 (dtd, *J* = 13.5, 10.3, 7.1 Hz, 3H), 1.65 (dt, *J* = 9.4, 5.3 Hz, 1H), 1.62 – 1.52 (m, 4H), 1.48 (dd, *J* = 14.2, 4.8 Hz, 1H), 1.41 – 1.30 (m, 3H), 1.07 (s, 3H), 0.96 (dd, *J* = 12.6, 1.6 Hz, 1H), 0.87 (d, *J* = 5.9 Hz, 6H), 0.86 (s, 3H), 0.82 (s, 3H), 0.64 (s, 3H).

^{13}C NMR (100 MHz, $CDCl_3$) δ 174.7, 170.9, 145.9, 117.5, 80.6, 72.2, 51.6, 50.2, 50.0, 48.8, 46.5, 45.2, 39.0, 37.8, 36.9, 36.7, 36.0, 35.7, 31.4, 31.3, 28.6, 28.1, 24.2, 22.1, 21.4, 18.3, 18.1, 16.8, 14.3.

IR: ν_{max} = 3556, 2946, 1732, 1372,1247, 1032 cm-1

HRMS (ESI-TOF): *m/z* for $[M-H]^-$ calcd.: 487.3423, found: 487.3412

m.p.: 153-155 °C

Opt. act.: $[\alpha]_D^{20}$= +12 (*c* = 0.25, $CHCl_3$)

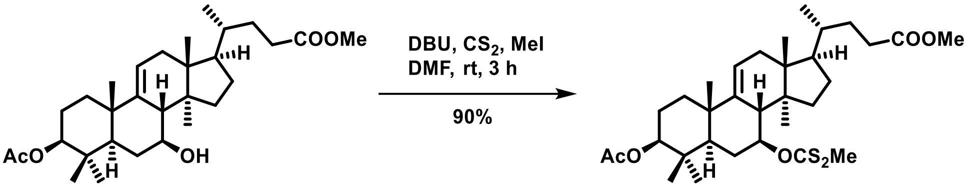

24 25

To a stirred solution of the alcohol **24** (2 gm, 4.09 mmol) in DMF (20 mL) was added DBU (3.67 mL, 24.55 mmol). The mixture was stirred at room temperature for 10 min and then CS_2 (3.70 mL, 61.38 mmol) was added. One hour later, MeI (1.29 mL, 20.46 mmol) was added. The solution was stirred at room temperature for further 1 h. The resulting mixture was diluted with CH_2Cl_2. The organic layer was washed with water and brine, respectively, and then dried over Na_2SO_4 and concentrated. The residue was purified by flash column chromatography (petroleum ether/EtOAc = 5:1, *v/v*) to afford the corresponding xanthate derivative **25** (2.13 gm, 3.68 mmol, 90%) as yellowish solid.

TLC: R_f = 0.3 (petroleum ether/EtOAc = 2:1, *v/v*)

^{1}H NMR (400 MHz, $CDCl_3$) δ 5.65 (td, *J* = 10.9, 5.1 Hz, 1H), 5.38 (d, *J* = 6.1 Hz, 1H), 4.47 (dd, *J* = 11.6, 3.8 Hz, 1H), 3.66 (s, 3H), 2.68 (d, *J* = 10.8 Hz, 1H), 2.57 (s, 3H), 2.41 – 2.17 (m, 3H), 2.09 (d, *J* = 17.8 Hz, 1H), 2.04 (s, 3H), 2.00 – 1.94 (m, 1H), 1.85 – 1.76 (m, 3H), 1.75 – 1.64 (m, 1H), 1.64 – 1.49 (m, 5H), 1.41 – 1.27 (m, 4H), 1.13 (s, 3H), 1.05 (d, *J* = 14.1 Hz, 1H), 0.88 (t, *J* = 3.1 Hz, 6H), 0.87 (s, 3H), 0.79 (s, 3H), 0.69 (s, 3H).

^{13}C NMR (100 MHz, $CDCl_3$) δ 214.74, 174.71, 170.89, 144.79, 118.76, 84.41, 80.44, 51.56, 50.07, 48.25, 46.87, 46.37, 44.91, 38.95, 37.96, 36.80, 35.89, 35.69, 35.54, 31.32, 31.29, 28.47, 27.97, 26.05, 24.07, 22.06, 21.35, 19.06, 18.67, 18.09, 16.69, 14.48.

IR: ν_{max} = 2953, 1740, 1727, 1362,1246, 1224, 1046 cm^{-1}

HRMS (ESI-TOF): *m/z* for $[M+Na]^+$ calcd. : 601.2997, found : 601.2991

m.p.: 129-130 °C

Opt. act.: $[\alpha]_D^{20}$= +57.6 (*c* = 0.25, $CHCl_3$)

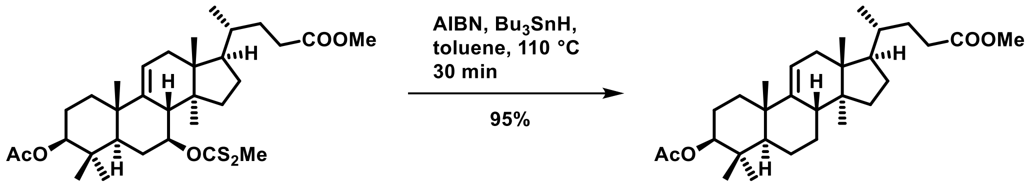

25 **13**

To a stirred solution of **25** (1 gm, 1.72 mmol) in toluene (10 mL) were added $^{n}Bu_3SnH$ (1 mL, 3.74 mmol) and AIBN (705.91 mg, 2.98 mmol). The mixture was stirred at 110 ^{o}C for 30 min, then cooled to room temperature and concentrated. The residue was purified by flash column chromatography (petroleum ether/EtOAc = 6:1, *v/v*) to afford **13** as a white solid (775 mg, 1.64 mmol, 95%).

TLC: R_f = 0.7 (petroleum ether/EtOAc = 9:1, *v/v*)

^{1}H NMR (400 MHz, $CDCl_3$): δ 5.20 (d, *J* = 6.1 Hz, 1H), 4.46 (dd, *J* = 11.6, 4.3 Hz, 1H), 3.64 (s, 3H), 2.36 (ddd, *J* = 15.3, 10.4, 5.2 Hz, 1H), 2.27 – 2.18 (m, 1H), 2.17 – 2.04 (m, 2H), 2.03 (s, 3H), 1.91 – 1.82 (m, 2H), 1.78 – 1.72 (m, 2H), 1.69 – 1.66 (m, 1H), 1.59 – 1.50 (m, 2H), 1.49 – 1.39 (m, 2H), 1.33 (q, *J* = 4.3 Hz, 4H), 1.30 – 1.19 (m, 4H), 1.04 (s, 3H), 0.95 (dd, *J* = 12.2, 2.4 Hz, 1H), 0.87 (s, 3H), 0.86 (d, *J* = 2.4 Hz, 3H), 0.84 (s, 3H), 0.71 (s, 3H), 0.62 (s, 3H).

^{13}C NMR (100 MHz, $CDCl_3$): δ 174.8, 171.1, 148.2, 115.1, 80.9, 52.6, 51.6, 50.8, 47.1, 44.4, 41.8, 39.3, 38.1, 37.2, 35.9, 35.8, 33.9, 31.4, 31.3, 28.3, 28.1, 28.0, 24.2, 22.4, 21.4, 21.3, 18.6, 18.0, 16.9, 14.5.

IR: ν_{max} = 2939, 2869, 1730, 1372, 1249, 1036 cm^{-1}

HRMS (ESI-TOF): *m/z* for $[M+Na]^+$ calcd. : 495.3553, found : 495.3433

m.p.: 151-153 ^{o}C

Opt. act.: $[\alpha]_D^{20}$= -5.6 (*c* = 0.25, $CHCl_3$)

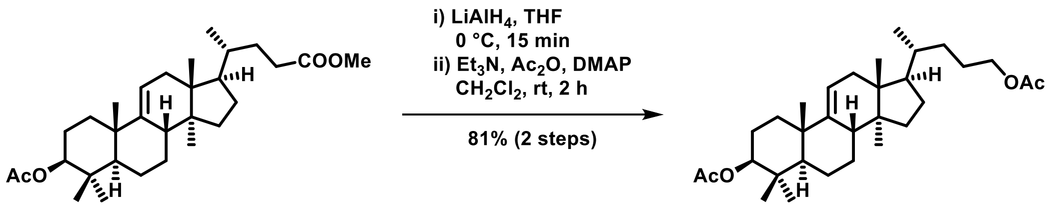

13 **14**

To a stirred solution of **13** (4 gm, 8.46 mmol) in THF (50 mL) was added $LiAlH_4$ (16.93 mmol) in portions at 0 ^{o}C. The mixture was stirred at same temperature for 15 min and was then quenched with sat. aq. NH_4Cl solution. The mixture was washed with brine and extracted with EtOAc (3x30 mL). The combined organic phase was dried over Na_2SO_4 and concentrated. The residue was purified by flash chromatography (petroleum ether/EtOAc = 1:1) to afford the corresponding diol as a white solid.

To the above white solid, CH_2Cl_2 (40 mL) was added followed by Et_3N (8.2 mL, 60.65 mmol) and Ac_2O (2.5 mL, 24.26 mmol) at 0 ^{o}C to rt under argon and stirred for 30

minutes. Then $NaHCO_3$ solution (sat. aq. 10 mL) was added and aqueous phase was extracted with CH_2Cl_2 (3x30 mL) and dried over Na_2SO_4 and concentrated. The residue was purified by column chromatography (silica gel, petroleum ether/EtOAc = 9:1, *v/v*) to afford pure di-acetate **14** as a white solid (3.31 g, 6.80 mmol, 81% over two steps).

TLC: R_f = 0.7 (petroleum ether/EtOAc = 9:1, *v/v*)

^{1}H NMR (400 MHz, $CDCl_3$): δ 5.19 (d, *J* = 6.0 Hz, 1H), 4.45 (dd, *J* = 11.5, 4.3 Hz, 1H), 4.09 – 3.89 (m, 2H), 2.12 (dd, *J* = 16.8, 6.9 Hz, 2H), 2.01 (s, 3H), 2.01 (s, 3H), 1.90 – 1.81 (m, 2H), 1.79 – 1.68 (m, 3H), 1.65 (dd, *J* = 10.2, 7.7 Hz, 3H), 1.60 – 1.48 (m, 3H), 1.43 (ddd, *J* = 15.0, 9.1, 4.9 Hz, 3H), 1.37 – 1.21 (m, 5H), 1.03 (s, 3H), 0.96 – 0.91 (m, 1H), 0.86 (t, *J* = 3.2 Hz, 6H), 0.83 (s, 3H), 0.70 (s, 3H), 0.61 (s, 3H).

^{13}C NMR (100 MHz, $CDCl_3$): δ 171.3, 171.0, 148.2, 115.2, 80.9, 65.2, 52.6, 50.9, 47.1, 44.4, 41.8, 39.3, 38.1, 37.2, 35.9, 35.8, 35.8, 34.0, 32.3, 28.3, 28.0, 25.5, 24.2, 22.4, 21.4, 21.3, 21.1, 18.6, 18.3, 16.9, 14.5.

IR: ν_{max} = 2917, 2870, 1736, 1711, 1369, 1239, 1040 cm^{-1}

HRMS (ESI-TOF): *m/z* for $[M-H]^-$ calcd. : 485.3629, found : 485.3985

m.p.: 144-147 °C

Opt. act.: $[\alpha]_D^{20}$= -2.4 (*c* = 0.25, $CHCl_3$)

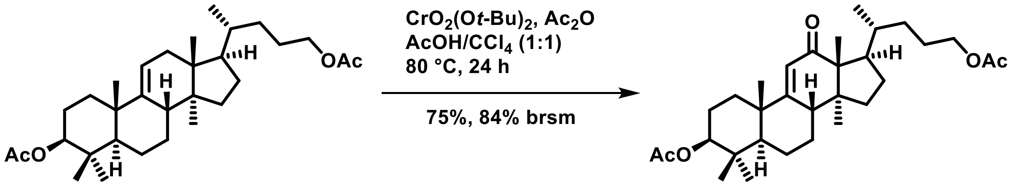

14 31

The 1 M *t*-butyl chromate ($CrO_2(t\text{-}BuO)_2$) solution was freshly prepared as follows: To a stirred solution of CrO_3 (1 gm, 10 mmol) in *t*-BuOH (1.9 mL, 20 mmol) was added CCl_4 (10 mL). The mixture was stirred at room temperature for 30 min. The $CrO_2(t\text{-}BuO)_2$ solution was directly used for allylic oxidation.[11]

To a stirred solution of **14** (1 gm, 2.05 mmol) in CCl_4/AcOH (30 mL/30 mL) was added Ac_2O (1.94 mL, 20.54 mmol) and freshly prepared $CrO_2(t\text{-}BuO)_2$ (10 mL, 10.27 mmol). The reaction mixture was stirred at 80 °C for 24 h. Then all volatile parts were removed in rotary evaporator and sat. aq. $NaHCO_3$ was added until the evolution of gas stopped. The mixture was extracted with CH_2Cl_2 (3×20 mL) and the organic layers were combined and dried over Na_2SO_4 and concentrated. The residue was purified by column chromatography (silica gel, petroleum ether/EtOAc = 4:1, *v/v*) to afford pure enone derivative **31** as a white solid (771 mg, 1.54 mmol, 75% yield, 84% brsm yield) and diacetate **14** was recovered as a white solid (108 mg, 0.21 mmol, 10%).

TLC: R_f = 0.4 (petroleum ether/EtOAc = 4:1, *v/v*)

^{1}H NMR (400 MHz, CDCl$_3$): δ 5.58 (d, *J* = 2.7 Hz, 1H), 4.49 (dd, *J* = 11.3, 4.5 Hz, 1H), 4.11 – 3.96 (m, 2H), 2.62 (ddd, *J* = 12.7, 5.7, 2.5 Hz, 1H), 2.16 (td, *J* = 10.2, 7.7 Hz, 1H), 2.06 (s, 3H), 2.05 (s, 3H), 2.04 – 1.92 (m, 1H), 1.86 – 1.73 (m, 5H), 1.72 – 1.63 (m, 2H), 1.61 – 1.55 (m, 2H), 1.55 – 1.45 (m, 3H), 1.45 – 1.26 (m, 4H), 1.19 (s, 3H), 1.09 (dd, *J* = 11.8, 2.3 Hz, 1H), 1.00 (s, 3H), 0.97 (d, *J* = 6.3 Hz, 3H), 0.93 (s, 3H), 0.89 (s, 3H), 0.75 (s, 3H).

^{13}C NMR (100 MHz, CDCl$_3$): δ 205.7, 171.4, 170.9, 167.1, 119.7, 80.0, 65.1, 57.7, 51.9, 50.1, 44.1, 43.3, 40.2, 38.3, 36.2, 35.2, 32.9, 32.2, 28.2, 28.1, 28.0, 25.9, 23.9, 22.2, 21.4, 21.1, 20.9, 19.4, 18.3, 17.1, 13.4.

IR: ν_{max} = 2953, 2924, 1737, 1683, 1460, 1377, 1242 cm^{-1}

HRMS (ESI-TOF): *m/z* for [M+Na]$^+$ calcd. : 523.3399, found : 523.3391

m.p.: 141-142 ^{o}C

Opt. act.: $[\alpha]_D^{20}$= +2.4 (*c* = 0.25, CHCl$_3$)

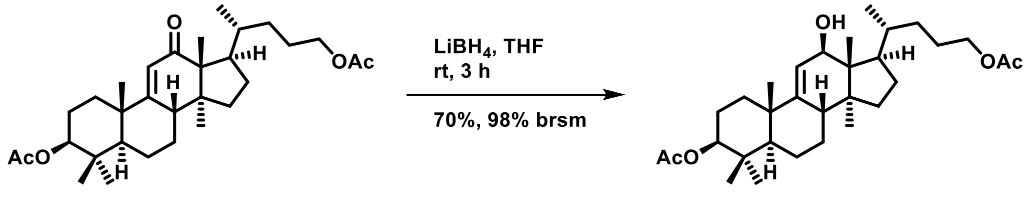

LiBH$_4$ (1 mL, 2 M in THF, 1.99 mmol) was added to a solution of compound **31** (500 mg, 0.99 mmol) in Dry THF (5 mL) at 0 ^{o}C and stirred at room temperature for 3 h. After that quenched the reaction by addition of sat. aq. NH$_4$Cl solution, diluted with brine and extract with EtOAc (3×20 mL) and the organic layers were combined and dried over Na$_2$SO$_4$ and concentrated in vacuo. The residue was purified by column chromatography (silica gel, petroleum ether/EtOAc = 7:3, *v/v*) to afford pure enol **15** (376 mg, 0.74 mmol, 70%, 98% brsm) as a white solid and starting material **31** (120 mg, 0.24 mmol, 24%) was recovered.

TLC: R$_f$ = 0.3 (petroleum ether/EtOAc = 4:1, *v/v*)

^{1}H NMR (396 MHz, CDCl$_3$) δ 5.47 (dd, *J* = 5.6, 1.6 Hz, 1H), 4.47 (dd, *J* = 11.2, 4.2 Hz, 1H), 4.10 – 3.96 (m, 2H), 3.86 (d, *J* = 5.3 Hz, 1H), 2.18 (dd, *J* = 18.6, 9.4 Hz, 2H), 2.04 (s, 3H), 2.03 (s, 3H), 1.98 – 1.89 (m, 1H), 1.80 (ddd, *J* = 12.4, 8.5, 3.4 Hz, 2H), 1.74 – 1.65 (m, 4H), 1.63 – 1.58 (m, 2H), 1.53 – 1.44 (m, 3H), 1.40 (dd, *J* = 12.0, 6.0 Hz, 2H), 1.33 (dd, *J* = 9.8, 6.6 Hz, 3H), 1.05 (s, 3H), 1.01 (s, 1H), 0.98 (d, *J* = 6.5 Hz, 3H), 0.89 (s, 3H), 0.88 (s, 3H), 0.86 (s, 3H), 0.61 (s, 3H).

^{13}C NMR (100 MHz, CDCl$_3$) δ 171.3, 171.0, 151.4, 117.4, 80.6, 74.6, 65.1, 52.2, 48.1, 46.0, 44.2, 41.8, 39.4, 38.1, 35.8, 35.7, 35.1, 32.3, 28.4, 28.3, 27.5, 25.6, 24.1, 22.6, 21.4, 21.1, 21.1, 20.4, 17.6, 16.9, 14.9.

IR: ν_{max} = 3516, 2945, 2870, 1733, 1365, 1238 cm^{-1}

HRMS (ESI-TOF): *m/z* for $[M-H]^-$ calcd. : 501.3580, found : 501.3563

m.p.: 152-155 ^oC

Opt. act.: $[\alpha]_D^{20}$= +89.6 (*c* = 0.25, $CHCl_3$)

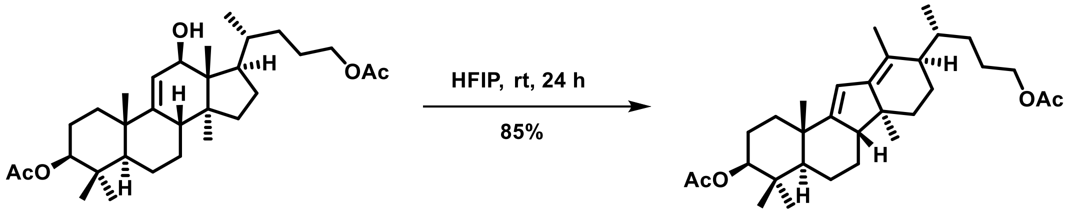

15 16

In compound **15** (50 mg, 0.09 mmol), HFIP (3.7 mL) was added, and the resultant mixture was stirred at room temperature for 24 h. The solvent was evaporated under vacuum, and the residue was purified by column chromatography (silica gel, petroleum ether/EtOAc = 95:5, *v/v*) to afford **16** as a viscous liquid (37 mg, 0.07 mmol, 85%).

TLC: R_f = 0.8 (petroleum ether/EtOAc = 95:5, *v/v*)

^{1}H NMR (400 MHz, $CDCl_3$): δ 5.78 (d, *J* = 2.1 Hz, 1H), 4.52 (dd, *J* = 11.0, 4.6 Hz, 1H), 3.99 (dtd, *J* = 17.7, 10.7, 6.9 Hz, 2H), 2.38 (dd, *J* = 11.7, 6.4 Hz, 1H), 2.05 (s, 3H), 2.04 – 2.01 (m, 1H), 2.00 (s, 3H), 1.88 (dd, *J* = 9.8, 3.0 Hz, 2H), 1.75 (dd, *J* = 9.1, 4.9 Hz, 2H), 1.68 (ddd, *J* = 13.3, 6.8, 3.1 Hz, 4H), 1.62 (d, *J* = 3.5 Hz, 1H), 1.60 (s, 3H), 1.59 (d, *J* = 3.2 Hz, 1H), 1.42 (dtd, *J* = 16.1, 12.8, 5.1 Hz, 4H), 1.29 – 1.20 (m, 3H), 1.07 (s, 3H), 1.03 – 1.00 (m, 1H), 0.96 (d, *J* = 6.8 Hz, 3H), 0.90 (s, 3H), 0.89 (s, 3H), 0.87 (s, 3H).

^{13}C NMR (100 MHz, $CDCl_3$): δ 171.3, 171.0, 158.7, 148.0, 120.2, 117.0, 80.8, 65.0, 52.4, 52.3, 43.1, 42.9, 38.0, 37.7, 35.4, 34.1, 33.7, 28.4, 27.4, 27.3, 26.4, 24.0, 22.5, 21.9, 21.4, 21.1, 20.9, 18.9, 18.9, 17.5, 16.9.

IR: ν_{max} = 2929, 1735, 1366, 1241, 1029, 607 cm^{-1}

HRMS (ESI-TOF): *m/z* for $[M+H]^+$ calcd. : 485.3631, found : 485.3613

Opt. act.: $[\alpha]_D^{20}$= -39.2 (*c* = 0.25, $CHCl_3$)

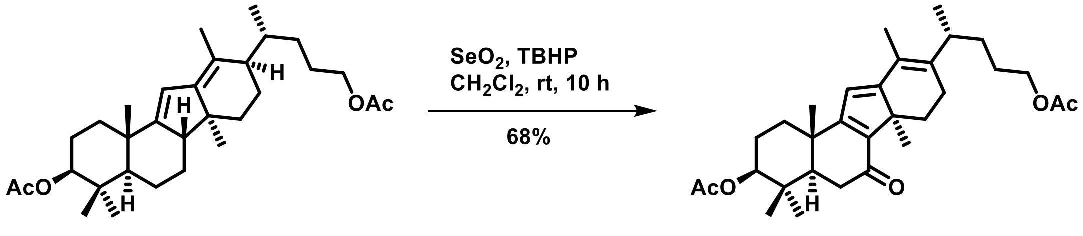

16 17

To a stirred solution of **16** (50 mg, 0.10 mmol) in CH_2Cl_2 (5 mL) was added Selenium (IV) oxide (11.45 mg, 0.10 mmol) and *t*-butyl hydroperoxide (5 M in decane solution, 0.10 mL, 0.51 mmol) at room temperature. The mixture was stirred at the same temperature for 10 h. After that reaction was quenched with sat. aq. sodium sulfite (2

mL) and extracted with CH_2Cl_2 (3×3 mL). The organic layers were washed with brine (2 mL) and the aqueous layers were back extracted with dichloromethane (5 mL) combined organic phase was dried over Na_2SO_4 and concentrated in vacuo. The residue was purified by flash chromatography (silica gel, petroleum ether/EtOAc = 4:1, *v/v*) to afford the corresponding tri-enone **17** as a yellow viscous liquid (33.77 mg, 0.06 mmol, 68%).

TLC: R_f = 0.8 (petroleum ether/EtOAc = 4:1, *v/v*)

^{1}H NMR (400 MHz, $CDCl_3$): δ 6.04 (s, 1H), 4.56 (dd, *J* = 11.4, 4.7 Hz, 1H), 4.02 (t, *J* = 6.2 Hz, 2H), 2.84 (dd, *J* = 13.9, 6.9 Hz, 1H), 2.52 – 2.33 (m, 3H), 2.27 (dt, *J* = 9.8, 8.2 Hz, 1H), 2.15 – 2.09 (m, 1H), 2.07 (s, 3H), 2.03 (s, 3H), 1.89 (s, 3H), 1.82 (ddd, *J* = 14.9, 13.2, 3.7 Hz, 3H), 1.63 (ddd, *J* = 17.0, 15.3, 5.4 Hz, 3H), 1.50 – 1.39 (m, 3H), 1.21 (s, 3H), 1.19 – 1.14 (m, 1H), 1.10 (s, 3H), 1.06 (d, *J* = 6.8 Hz, 3H), 0.99 (s, 3H), 0.92 (s, 3H).

^{13}C NMR (100 MHz, $CDCl_3$): δ 193.42, 172.56, 171.11, 170.79, 169.43, 144.73, 141.00, 122.82, 116.53, 79.84, 64.53, 51.32, 49.65, 37.69, 36.20, 35.63, 35.24, 34.28, 30.88, 30.39, 27.57, 26.92, 23.78, 22.07, 21.21, 20.97, 20.04, 19.81, 19.66, 16.28, 14.29.

IR: ν_{max} = 2956, 2924, 2849, 1738, 1639, 1242, 731 cm^{-1}

HRMS (ESI-TOF): *m/z* for $[M+H]^+$ calcd. : 497.3267, found : 497.3267

Opt. act.: $[\alpha]_D^{20}$= -25.6 (*c* = 0.25, $CHCl_3$)

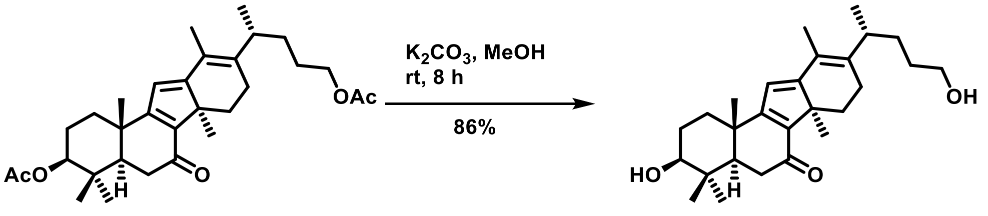

17 26

To the stirred solution of **17** (60 mg, 0.12 mmol) in 2 mL of MeOH, K_2CO_3 (50 mg, 0.36 mmol) was added at rt and stirred for 8 hrs. On complete conversion, MeOH was evaporated through a rotary evaporator then the yellowish solid was dissolved with H_2O (5 mL) and extracted with CH_2Cl_2 (3x5 mL) and dried over Na_2SO_4 and concentrated in vacuo. At last, the residue was purified by column chromatography (silica gel, petroleum ether/EtOAc = 2:1, *v/v*) to afford diol **26** as a yellow solid (43 mg, 0.10 mmol, 86%).

TLC: R_f = 0.2 (petroleum ether/EtOAc = 2:1, *v/v*)

^{1}H NMR (400 MHz, $CDCl_3$): δ 6.04 (s, 1H), 4.56 (dd, *J* = 11.4, 4.7 Hz, 1H), 4.02 (t, *J* = 6.2 Hz, 2H), 2.84 (dd, *J* = 13.9, 6.9 Hz, 1H), 2.52 – 2.33 (m, 3H), 2.27 (dt, *J* = 9.8, 8.2 Hz, 1H), 2.15 – 2.09 (m, 1H), 2.07 (s, 3H), 2.03 (s, 3H), 1.89 (s, 3H), 1.82 (ddd, *J* = 14.9, 13.2, 3.7 Hz, 3H), 1.63 (ddd, *J* = 17.0, 15.3, 5.4 Hz, 3H), 1.50 – 1.39 (m, 3H),

1.21 (s, 3H), 1.19 – 1.14 (m, 1H), 1.10 (s, 3H), 1.06 (d, J = 6.8 Hz, 3H), 0.99 (s, 3H), 0.92 (s, 3H).

^{13}C NMR (100 MHz, $CDCl_3$): δ 193.8, 172.9, 169.5, 145.0, 141.0, 122.7, 116.5, 78.3, 63.1, 51.3, 49.6, 38.8, 36.3, 35.9, 35.4, 34.6, 31.0, 30.8, 30.5, 27.6, 27.4, 22.1, 20.0, 19.8, 19.8, 15.2, 14.3.

IR: ν_{max} = 3284, 2921, 2852, 1732, 1640, 1391 cm^{-1}

HRMS (ESI-TOF): *m/z* for $[M+H]^+$ calcd. : 413.3056, found : 413.3059

m.p.: 159-160 ^oC

Opt. act.: $[\alpha]_D^{20}$= -4.8 (c = 0.25, $CHCl_3$)

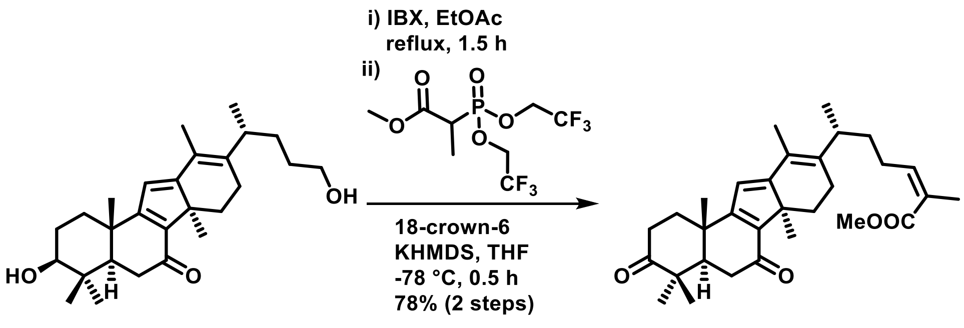

26 **18**

A solution of **19** (50 mg, 0.12 mmol) in EtOAc (2 mL) was refluxed with IBX (67.86 mg, 0.24 mmol) for 1.5 h. After that TLC indicated complete consumption of the starting material and the reaction mixture was filtered through a short Celite pad with a thin silica gel layer on top and washed with EtOAc (15 mL). Then it was concentrated through a rotary evaporator to afford a yellowish solid. The crude aldehyde was carried to the next step without purification.

To a solution of 18-crown-6 (800 mg, 3.02 mmol) in dry THF (5 mL) at –78 °C under argon was added the phosphonate (201.23 mg, 0.60 mmol) via a micro-syringe. KHMDS (0.6 mL, 0.60 mmol 1.0 M in THF) was then added to the mixture at the same temperature.[13] The crude aldehyde in THF (3 mL + 1 mL wash) was added to the reaction mixture and stirred at –78 °C. TLC after 30 min indicated complete consumption of aldehyde. Sat. aq. NH_4Cl solution (10 mL) was then added to the mixture at –78 °C and the reaction was warmed to room temperature. The aqueous layer was extracted with ethyl acetate (3 x 10 mL), and the organic layers were combined and dried over Na_2SO_4 and concentrated. The residue was purified by column chromatography (silica gel, petroleum ether/EtOAc = 4:1, *v/v*) to afford product **18** as a viscous liquid (44.80 mg, 0.09 mmol, 78% yield).

TLC: R_f = 0.2 (petroleum ether/EtOAc = 2:1, *v/v*)

^{1}H NMR (400 MHz, $CDCl_3$): ^{1}H NMR (400 MHz, $CDCl_3$) δ 6.03 (s, 1H), 5.88 (td, J = 7.6, 2.1 Hz, 1H), 3.67 (s, 3H), 2.90 – 2.69 (m, 2H), 2.60 – 2.50 (m, 2H), 2.44 – 2.23 (m, 8H), 2.14 (dd, J = 20.1, 7.3 Hz, 1H), 2.00 – 1.90 (m, 1H), 1.87 (s, 3H), 1.84 (s, 3H),

1.54 – 1.44 (m, 2H), 1.32 (s, 3H), 1.22 – 1.18 (m, 1H), 1.14 (s, 3H), 1.12 (s, 3H), 1.11 (s, 3H), 1.05 (d, *J* = 6.7 Hz, 3H).

^{13}C NMR (100 MHz, $CDCl_3$): δ 215.1, 192.8, 171.0, 170.0, 168.5, 145.5, 143.3, 141.3, 127.0, 122.8, 116.5, 51.4, 51.3, 49.8, 47.4, 36.5, 36.0, 35.5, 35.1, 34.5, 34.4, 30.6, 28.1, 25.9, 22.3, 21.3, 20.8, 20.0, 19.7, 19.6, 14.3.

IR: ν_{max} = 2713, 1612, 1561, 1479, 1276, 908 cm^{-1}

HRMS (ESI-TOF): *m/z* for $[M+H]^+$ calcd. : 479.3161, found : 479.3158

Opt. act.: $[\alpha]_D^{20}$= -7.2 (*c* = 0.25, $CHCl_3$)

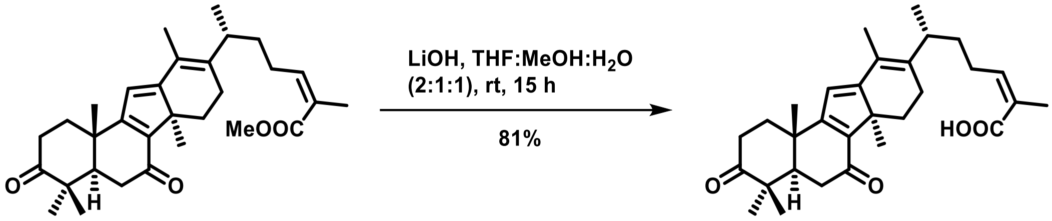

18 Kadcoccitane H (6)

To the stirred solution of **18** (30 mg, 0.06 mmol) in mixture of THF (1 mL), MeOH (0.5 mL) and H_2O (0.5 mL) was added LiOH (4 mg, 0.09 mmol) was added at room temperature and stirred for 15 hrs. On complete conversion, all volatile parts were removed in rotary evaporator then quenched with 1N HCl (3 mL) and the pH of the reaction mixture was adjusted to 1-2 and extracted with CH_2Cl_2 (3x5 mL) and dried over Na_2SO_4 and concentrated in vacuo. At last, the residue was purified by column chromatography (silica gel, petroleum ether/EtOAc = 1:1, *v/v*) to afford the pure kadcoccitane H **(6)** as a yellow amorphous solid (22.7 mg, 0.0489 mmol, 81%).

TLC: R_f = 0.2 (petroleum ether/EtOAc = 2:1, *v/v)*

^{1}H NMR (126 MHz, PYRIDINE-*D*$_5$): δ 6.31 (s, 1H), 6.00 (t, *J* = 7.5 Hz, 1H), 2.96 – 2.91 (m, 1H), 2.82 – 2.77 (m, 1H), 2.74 (dd, *J* = 12.5, 5.7 Hz, 1H), 2.73 – 2.69 (m, 1H), 2.70 – 2.63 (m, 2H), 2.55 (ddd, *J* = 15.9, 6.8, 3.3 Hz, 1H), 2.52 (dd, *J* = 16.6, 3.4 Hz, 1H), 2.42 (dd, *J* = 13.8, 3.3 Hz, 1H), 2.38 – 2.32 (m, 1H), 2.28 (dd, *J* = 18.4, 6.0 Hz, 1H), 2.19 – 2.14 (m, 1H), 2.13 (s, 3H), 2.04 (s, 3H), 1.91 – 1.84 (m, 1H), 1.61 – 1.56 (m, 1H), 1.55 – 1.51 (m, 1H), 1.51 – 1.46 (m, 1H), 1.40 (s, 3H), 1.25 (s, 3H), 1.14 (s, 3H), 1.11 (s, 3H), 1.08 (d, *J* = 6.9 Hz, 3H).

^{13}C NMR (126 MHz, PYRIDINE-*D*$_5$): δ 213.9, 192.1, 170.4, 170.3, 168.9, 145.3, 141.7, 141.4, 128.8,122.9, 117.3, 51.3, 49.9, 47.1, 36.6, 36.0, 35.6, 34.8, 34.7, 34.4, 30.9, 28.3, 25.7, 22.5, 21.3, 21.0, 20.1, 19.5, 19.0, 14.2.

IR: ν_{max} = 3053, 2711, 1609, 1584, 1560, 1275, 1149 cm^{-1}

HRMS (ESI-TOF): *m/z* for $[M+H]^+$ calcd. : 465.3005, found : 465.2998

Opt. act.: $[\alpha]_D^{20}$= -275.7 (*c* = 0.2, MeOH)

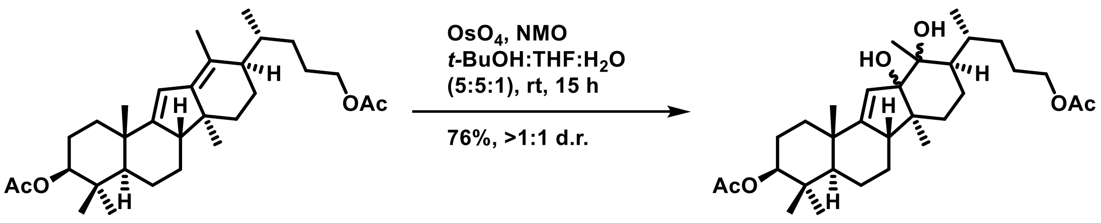

16 **19**

To a stirred solution of diene **16** (50 mg, 0.10 mmol) in *t*-BuOH (1 mL), THF (1 mL) and H_2O (0.2 mL). OsO_4 (0.16 M in *t*-BuOH, 1.1 mL, 0.10 mmol), N-methylmorpholine N-oxide (NMO) (24.16 mg, 0.20 mmol) were added at room temperature. After the reaction mixture was stirred at room temperature for 15 h, saturated aqueous $Na_2S_2O_3$ (3 mL) was added to the mixture. The resultant mixture was extracted with CH_2Cl_2 (3 mL×3). The combined organic layers were washed with brine (3 mL), dried over Na_2SO_4, filtered and concentrated in vacuo. The residue was purified by column chromatography (silica gel, petroleum ether/EtOAc = 9:1, *v/v*) to afford **19** as inseparable mixture of diastereomers as blackish viscous liquid (40 mg, 0.07 mmol, 76%, >1:1 d.r.).

TLC: R_f = 0.8 (petroleum ether/EtOAc = 9:1, *v/v*)

^{1}H NMR (400 MHz, $CDCl_3$) δ 5.16 (s, 1H), 5.13 (s, 1H), 4.48 – 4.43 (m, 2H), 4.02 – 3.99 (m, 4H), 2.63 – 2.33 (m, 4H), 2.03 (s, 6H), 2.02 (s, 6H), 1.74 – 1.61 (m, 24H), 1.39 (s, 3H), 1.38 (s, 3H), 1.36 – 1.29 (m, 6H), 1.07 (s, 6H), 1.02 (s, 6H), 0.98 (dd, *J* = 11.0, 6.1 Hz, 4H), 0.94 – 0.90 (m, 6H), 0.88 (s, 9H), 0.85 (s, 9H).

^{13}C NMR (101 MHz, $CDCl_3$) δ 171.3, 170.9, 120.7, 118.1, 80.6, 65.1, 64.9, 52.6, 52.4, 50.6, 48.6, 48.4, 46.8, 46.0, 38.2, 37.9, 37.5, 35.0, 33.7, 32.1, 31.4, 30.0, 28.2, 27.3, 26.8, 23.8, 21.3, 21.0, 20.9, 19.9, 16.9

IR: ν_{max} = 3430, 2926, 2870, 1508, 1365, 1234, 972 cm^{-1}

HRMS (ESI-TOF): *m/z* for $[M-H]^-$ calcd. : 517.3529, found : 517.3526

Opt. act.: $[\alpha]_D^{20}$= -294 (*c* = 0.07, $CHCl_3$)

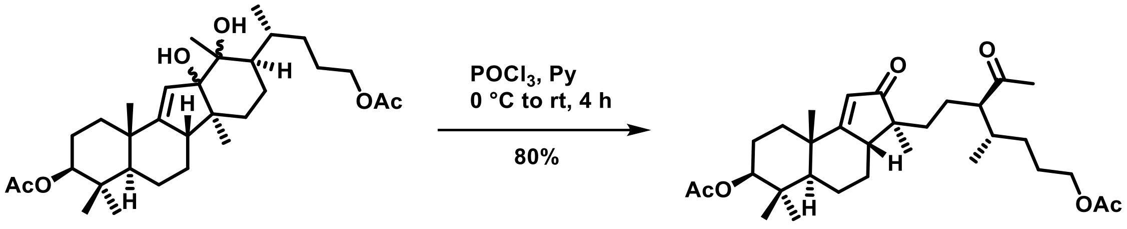

19 **20**

To a stirred solution of diol **19** (25 mg, 0.04 mmol) in pyridine (2 mL) was added $POCl_3$ (8 µL, 0.08 mmol) at 0 °C and the reaction mixture was stirred at rt for 4 h. After that the mixture was carefully neutralized with 1 N HCl and extracted with CH_2Cl_2. The organic extracts were combined, dried over Na_2SO_4, filtered and concentrated in

vacuo. The residue was purified by column chromatography (silica gel, petroleum ether/EtOAc = 9:1, *v/v*) to afford **20** as viscous liquid (16.53 mg, 0.03 mmol, 80%).

TLC: R_f = 0.3 (petroleum ether/EtOAc = 4:1, *v/v*)

^{1}H NMR (400 MHz, $CDCl_3$): δ 5.60 (d, *J* = 1.5 Hz, 1H), 4.50 – 4.45 (m, 1H), 4.01 (td, *J* = 6.7, 1.4 Hz, 2H), 2.67 – 2.57 (m, 1H), 2.25 (q, *J* = 6.6 Hz, 1H), 2.07 (s, 3H), 2.05 (s, 3H), 2.03 (s, 3H), 1.86 – 1.68 (m, 7H), 1.61 (dd, *J* = 12.7, 2.4 Hz, 1H), 1.53 – 1.48 (m, 1H), 1.46 – 1.37 (m, 3H), 1.33 – 1.25 (m, 2H), 1.19 (s, 3H), 1.17 – 1.06 (m, 4H), 1.05 – 1.00 (m, 2H), 0.95 (s, 3H), 0.92 (s, 3H), 0.89 (d, *J* = 6.7 Hz, 3H), 0.87 (s, 3H).

^{13}C NMR (100 MHz, $CDCl_3$): δ 214.00, 212.25, 190.80, 171.23, 170.87, 119.85, 80.02, 64.51, 58.75, 52.92, 49.24, 47.83, 39.60, 38.54, 37.69, 34.73, 34.51, 30.94, 30.58, 29.72, 28.15, 26.21, 23.69, 22.84, 21.31, 21.06, 20.03, 17.57, 17.53, 16.85.

IR: ν_{max} = 2926, 2870, 1732, 1365, 1234, 1028 cm^{-1}

HRMS (ESI-TOF): *m/z* for $[M+H]^+$ calcd. : 539.3349, found : 539.3333

Opt. act.: $[\alpha]_D^{20}$= -35.6 (*c* = 0.50, $CHCl_3$)

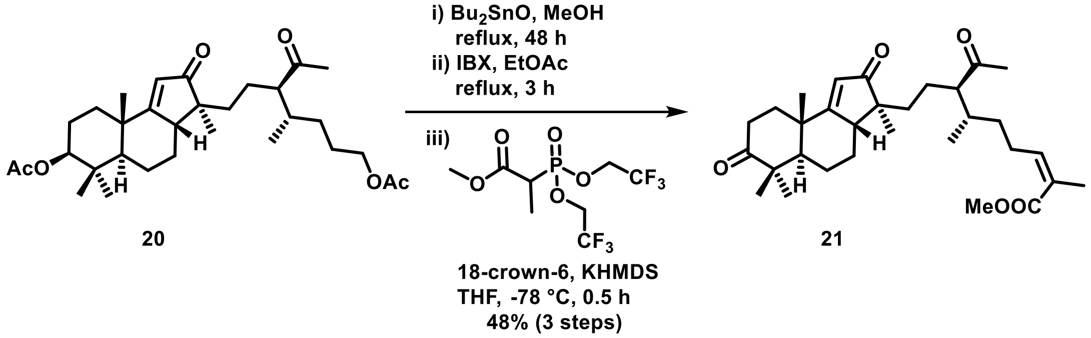

A solution of **20** (40 mg, 0.07 mmol) in MeOH (7 mL) was refluxed with Bu_2SnO (436.07 mg, 1.75 mmol) for 48 h. After that the reaction mixture was filtered through a short Celite pad and washed with EtOAc (15 mL). The solvent was evaporated under reduced pressure and the crude product purified by flash chromatography on silica gel (petroleum ether/EtOAc = 1:4, *v/v*) to afford the corresponding diol as a yellow solid.

To a stirred solution of diol in EtOAc (2 mL) was refluxed with IBX (47.22 mg, 0.16 mmol) for 3 h. After that TLC indicated complete consumption of the starting material, the reaction mixture was filtered through a short Celite pad with a thin silica gel layer on top and washed with EtOAc (10 mL). Then it was concentrated through a rotary evaporator to afford a yellowish solid. The crude aldehyde was carried to the next step without purification.

To a solution of 18-crown-6 (445 mg, 1.68 mmol) in dry THF (3 mL) at –78 °C under argon was added the phosphonate (112.03 mg, 0.33 mmol) via a micro-syringe. KHMDS (1.0 M in THF, 0.3 mL, 0.33 mmol) was then added to the mixture at the same temperature.[13] The crude aldehyde in THF (2 mL + 1 mL wash) was added to the reaction mixture and stirred at –78 °C. TLC after 30 min indicated complete consumption of aldehyde. Sat. aq. NH_4Cl solution (10 mL) was then added to the

mixture at –78 °C and the reaction was warmed to room temperature. The aqueous layer was extracted with ethyl acetate (3 x 10 mL), and the organic layers were combined and dried over Na_2SO_4 and concentrated. The residue was purified by column chromatography (silica gel, petroleum ether/EtOAc = 2:1, *v/v*) to afford product **21** as a viscous liquid (18.54 mg, 0.04 mmol, 73% yield).

TLC: R_f = 0.5 (petroleum ether/EtOAc = 3:1, *v/v*)

^{1}H NMR (400 MHz, $CDCl_3$): δ 5.86 (t, *J* = 7.3 Hz, 1H), 5.70 (s, 1H), 3.72 (s, 3H), 2.93 – 2.63 (m, 2H), 2.58 – 2.43 (m, 2H), 2.43 – 2.28 (m, 2H), 2.10 (s, 3H), 2.06 – 1.99 (m, 2H), 1.95 (dd, *J* = 13.4, 5.5 Hz, 1H), 1.88 (s, 3H), 1.75 (td, *J* = 9.2, 3.4 Hz, 3H), 1.48 – 1.41 (m, 2H), 1.39 (s, 3H), 1.37 – 1.32 (m, 2H), 1.21 – 1.15 (m, 3H), 1.13 (s, 3H), 1.10 (s, 3H), 1.05 – 1.00 (m, 1H), 0.92 (s, 3H), 0.73 (d, *J* = 6.7 Hz, 3H).

^{13}C NMR (100 MHz, $CDCl_3$): δ 214.9, 213.8, 212.4, 189.2, 168.4, 143.0, 127.3, 120.7, 58.7, 53.4, 49.3, 48.3, 47.9, 39.4, 37.7, 35.4, 34.7, 33.0, 32.0, 30.7, 27.1, 25.9, 22.8, 22.3, 22.1, 20.8, 19.2, 17.5, 14.2.

IR: ν_{max} = 2924, 2852, 1869, 1791, 1716, 1653, 1217 cm^{-1}

HRMS (ESI-TOF): *m/z* for $[M+H]^+$ calcd. : 499.3423, found : 499.3421

Opt. act.: $[\alpha]_D^{20}$= -2.66 (*c* = 0.37, $CHCl_3$)

5) NMR data comparison table: -

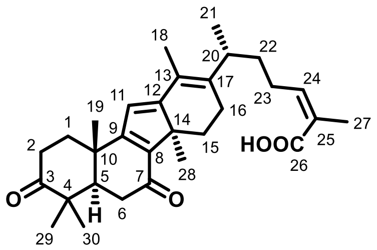

Kadcoccitane H (6)

Position	Natural Kadcoccitane H (^{1}H-NMR)[5]	Synthetic Kadcoccitane H (^{1}H-NMR)	Position	Natural Kadcoccitane H (^{13}C-NMR)[5]	Synthetic Kadcoccitane H (^{13}C-NMR)
1α	1.83, m	1.84, m	1	35.0	34.8
1β	2.15, m	2.14, m	2	34.6	34.7
2α	2.56, ddd (15.9, 6.8, 3.3)	2.55, ddd(15.9, 6.8, 3.3)	3	214.1	213.9
2β	2.77,m	2.77, m	4	47.3	47.3
5	2.41, dd(13.9,3.3)	2.42, dd(13.8,3.3)	5	51.5	51.3
6α	2.51, dd(16.5,3.3)	2.52, dd(16.6,3.4)	6	36.8	36.6
6β	2.65, overlap	2.65, overlap	7	192.3	192.1
11	6.30, s	6.31, s	8	141.6	141.4
15α	2.74, dd(12.5,5.7)	2.74, dd(12.5,5.7)	9	170.6	170.4
15β	1.48, m	1.46, m	10	36.2	36.0
16α	2.33, m	2.32, m	11	117.5	117.3
16β	2.27, dd(18.4,5.8)	2.28, dd(18.4,6.0)	12	169.1	168.9
18a	2.02, s	2.04, s	13	123.2	122.9
19	1.24, s	1.25, s	14	50.1	49.9
20	2.92, m	2.94, m	15	31.1	30.9
21	1.06, d(6.9)	1.08, d(6.9)	16	22.7	22.5
22a	1.59, m	1.56, m	17	145.5	145.3
22b	1.54, m	1.51, m	18	14.4	14.2
23a	2.70, m	2.73, m	19	19.2	19.0
23b	2.65, overlap	2.63, m	20	35.8	35.6
24	5.98, t(7.7)	6.00, t(7.5)	21	19.7	19.5
27	2.12, s	2.13, s	22	34.9	34.7
28	1.38, s	1.40, s	23	28.5	28.3
29	1.10, s	1.11, s	24	141.8	141.7
30	1.12, s	1.14, s	25	129.0	128.8
			26	170.6	170.4
			27	21.6	21.3
			28	20.3	20.1
			29	21.2	21.0
			30	25.8	25.7

6) X-ray crystallographic data: -

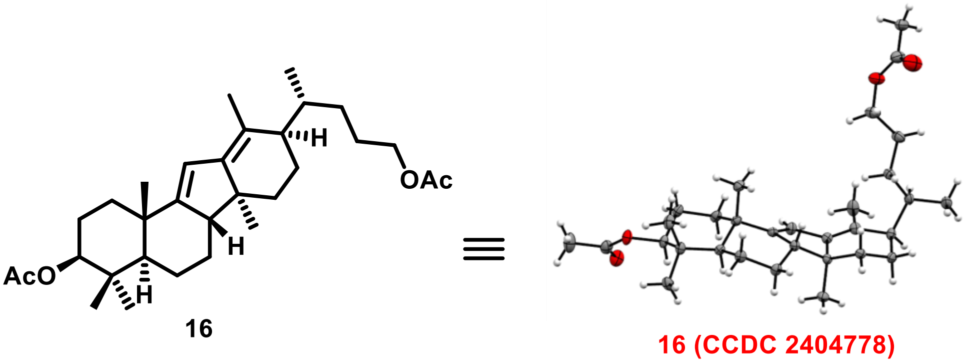

16

16 (CCDC 2404778)

Structure of compound **16** (CCDC 2404778), the anisotropic displacement parameters are drawn at the 50% probability displacement level. Red = Oxygen atom; Grey = Carbon atom; White = Hydrogen atom. Crystal was grown in CH_2Cl_2/Petroleum Ether = 1:1; v/v, CCD Bruker SMART APEX diffractometer, structure was solved by direct methods and refined (SHELXL-97) by full matrix least squares based on F^2 to R(reflections) = 0.0457 (6340) [I > 20(1)].

Structure Analysis-

CCDC	2404778
Formula	$C_{31}H_{48}O_4$
Crystal Color	white
Mr.	484.724 g mol^{-1}
cryst. system	Orthorhombic
space group	$P2_12_12_1$
Hall group	P 2ac 2ab
a, Å	7.5983(2)
b, Å	14.6061(3)
c, Å	25.2870(6)
α, (º)	90
B, (º)	90
γ, (º)	90
Z	4
V, $Å^3$	2806.39(11)
T, K	296.15 K
D_{calc}, g cm^{-3}	1.147
D_{report}, g cm^{-3}	1.147
λ, Å	0.71073
μ(Mo Kα), mm^{-1}	0.074
T_{min}	0.6695
T_{max}	0.7457
θ range (º)	2.79 to 28.00
refl. Collected	6340
R (reflections)	0.0457 (6340)
wR_2(reflections)	0.1240 (6968)
GOF on F^2	1.0373

7) References: -

5. Q.-Q. Zhang, K. Hu, H.-D. Sun and P.-T. Puno, *Nat. Products Bioprospect.*, 2023, **13**, 12.

9. X. Chen, X. Shao, W. Li, X. Zhang and B. Yu, *Angew. Chemie Int. Ed.*, 2017, **56**, 7648–7652.

11. (a) A. Srikrishna and D. H. Dethe, *Org. Lett.*, 2003, **5**, 2295–2298. (b) W. Liu, Z. Yu and N. Winssinger, *Org. Lett.*, 2021, **23**, 969–973.

13. W. C. Still and C. Gennari, *Tetrahedron Lett.*, 1983, **24**, 4405–4408.

8) ^{1}H & ^{13}C NMR data:-

^{1}H NMR of **11**

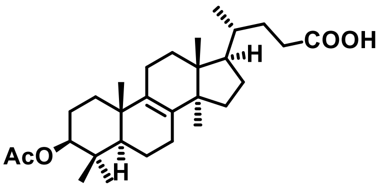

11

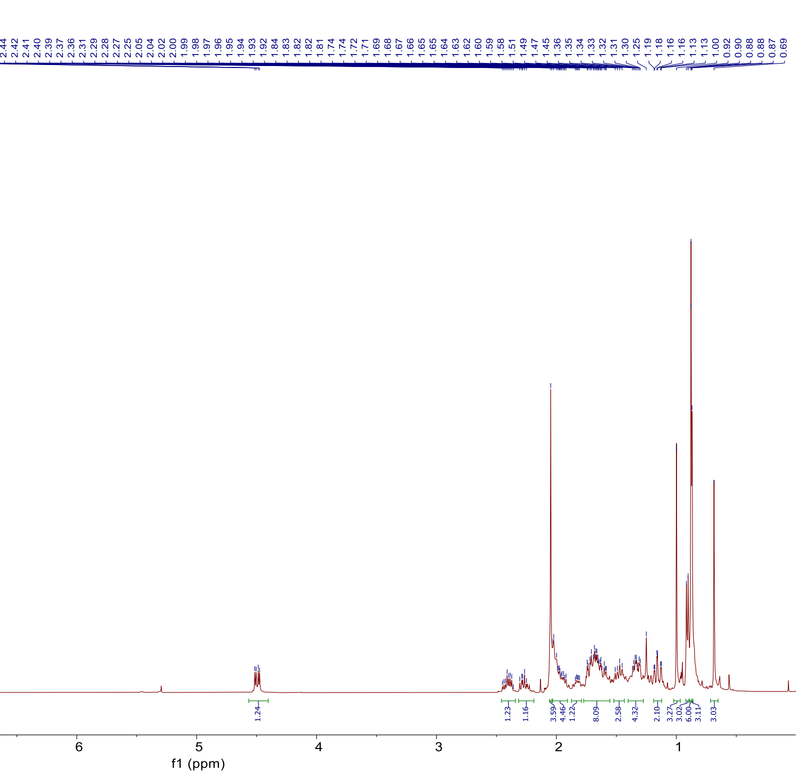

^{13}C NMR of **11**

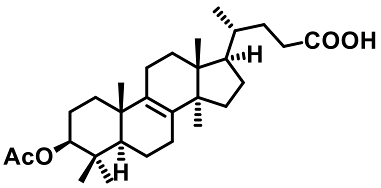

11

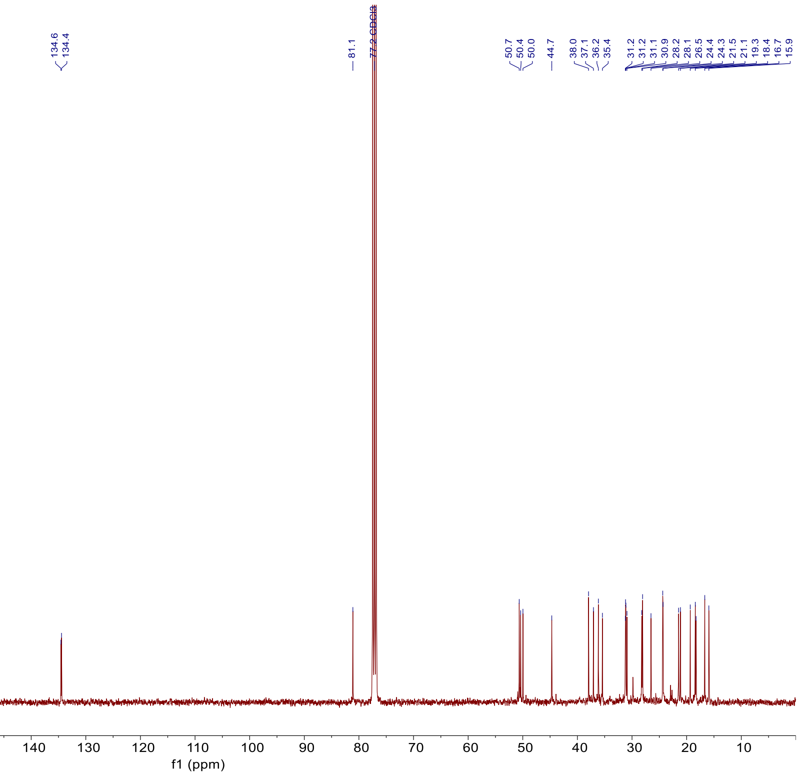

^{1}H NMR of **23**

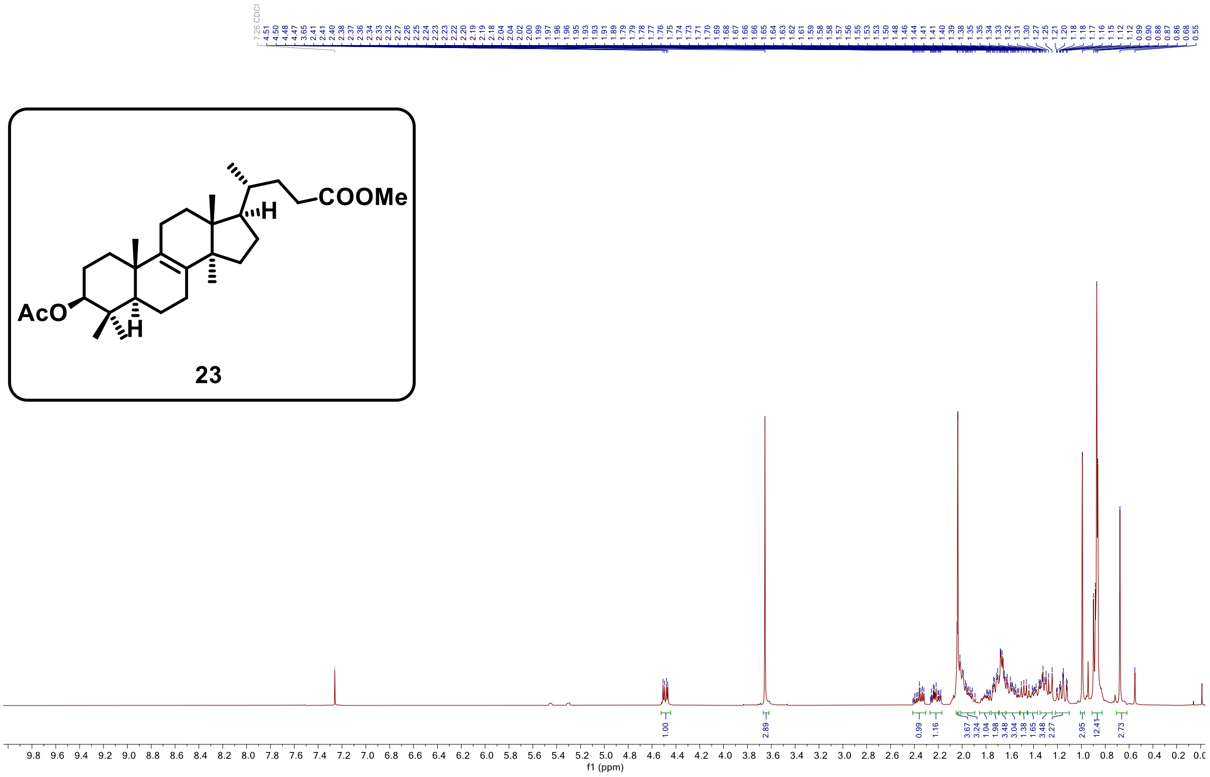

^{13}C NMR of **23**

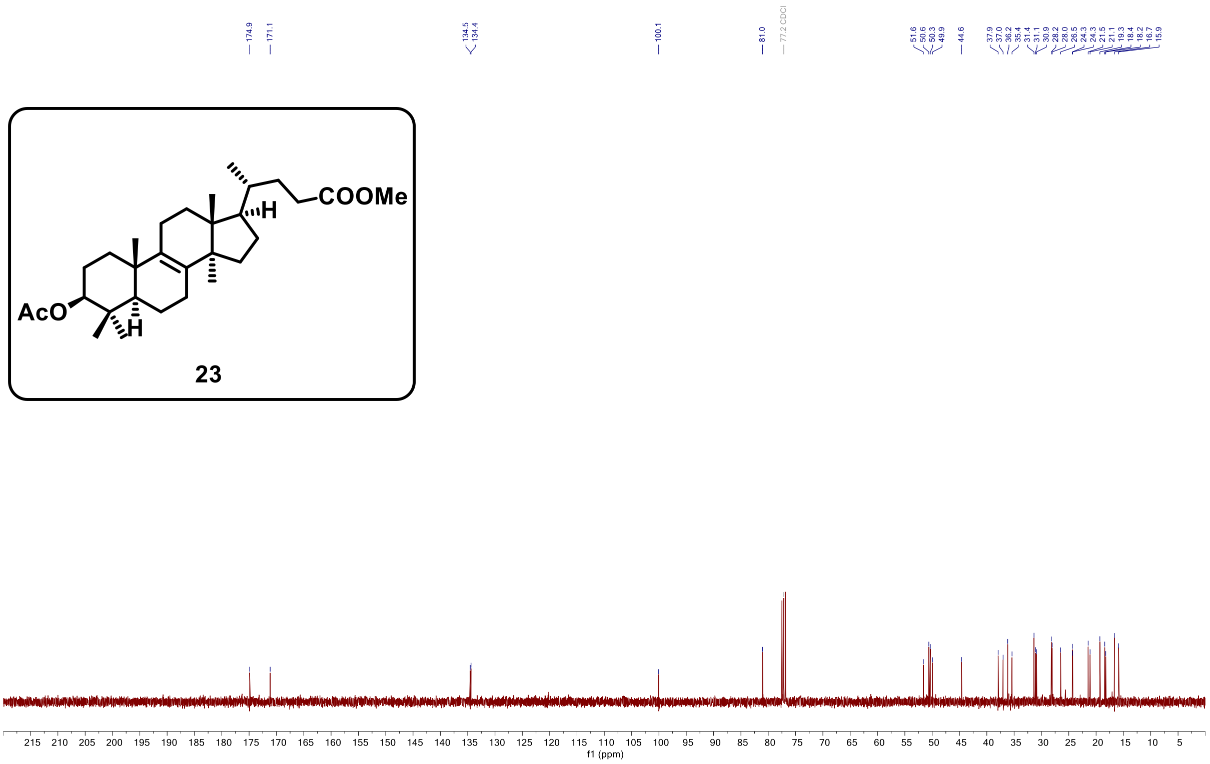

^{1}H NMR of **12**

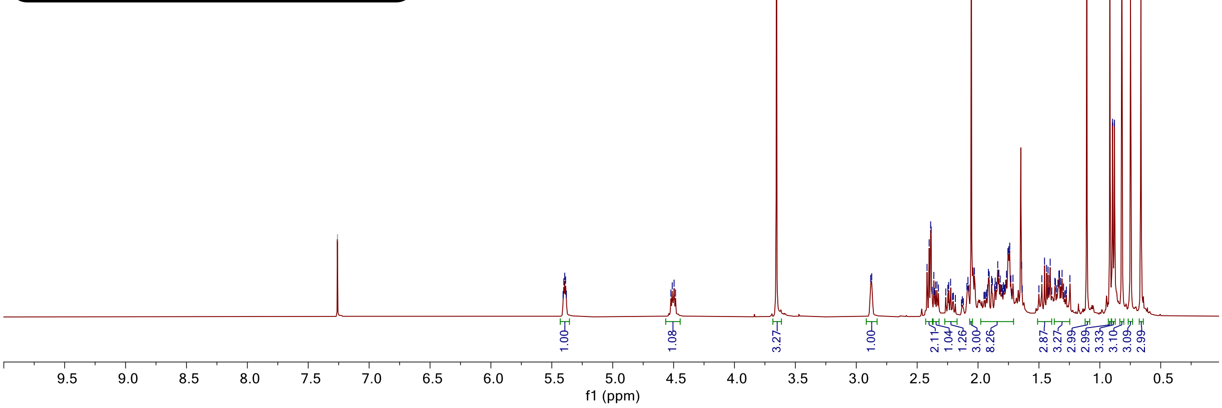

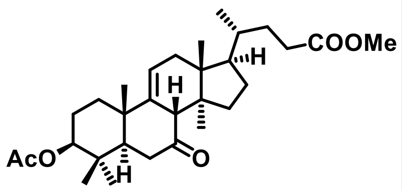

12

^{13}C NMR of **12**

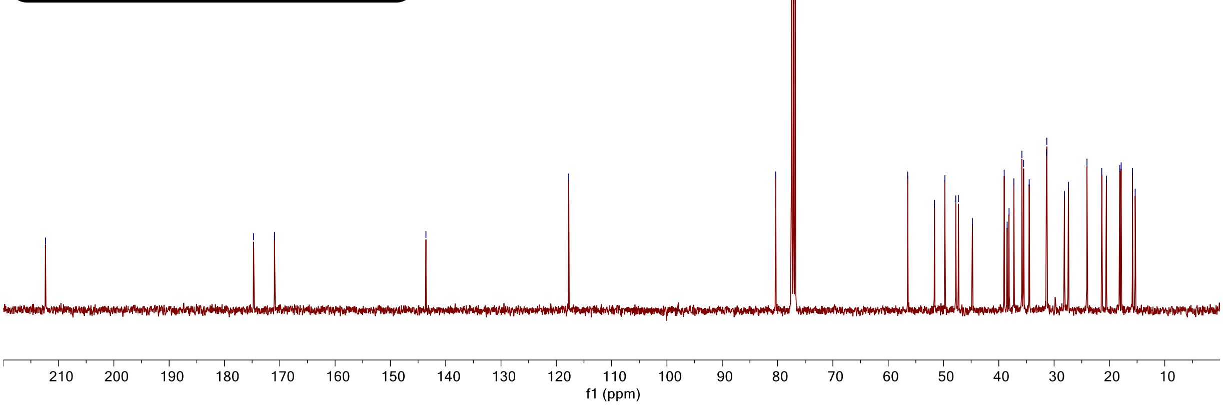

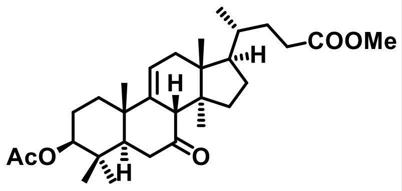

12

^{1}H NMR of **24**

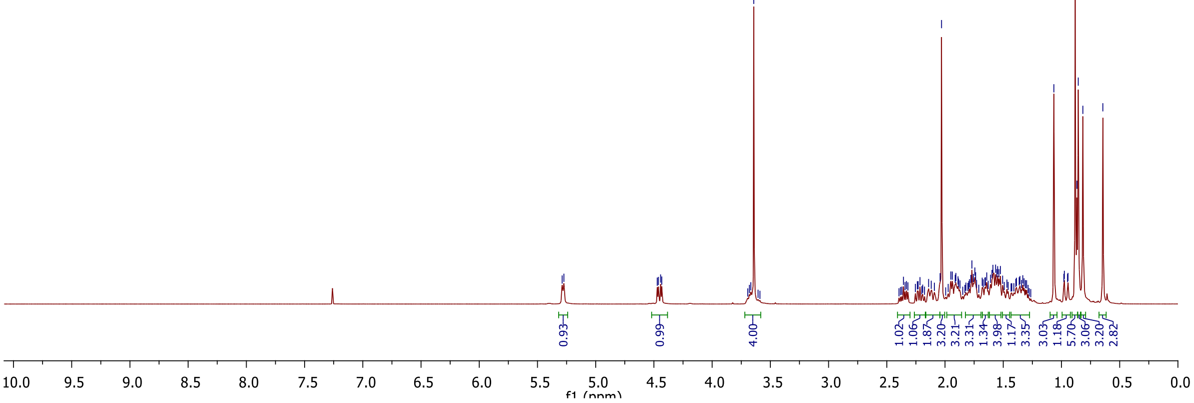

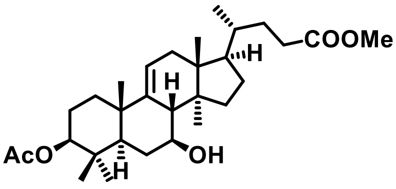

24

^{13}C NMR of **24**

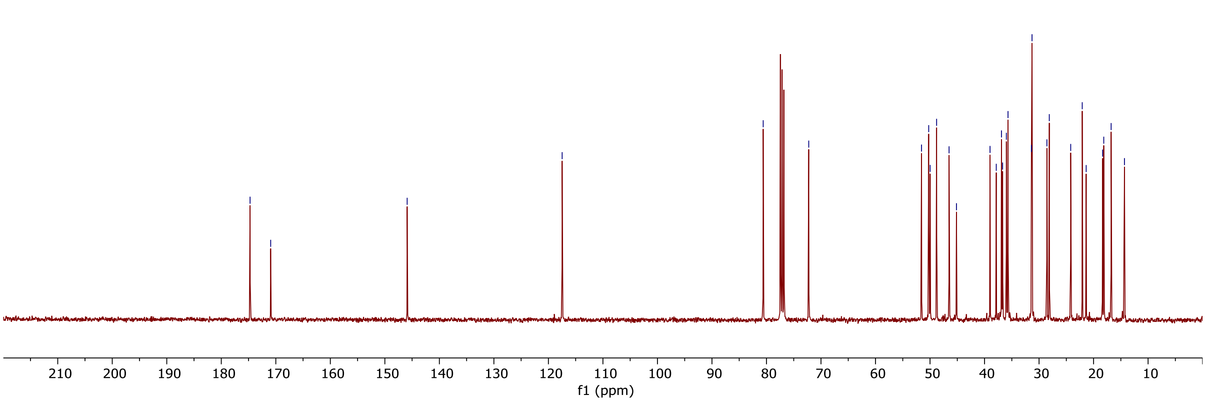

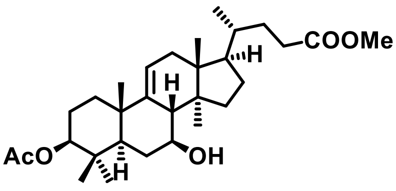

24

^{1}H NMR of **25**

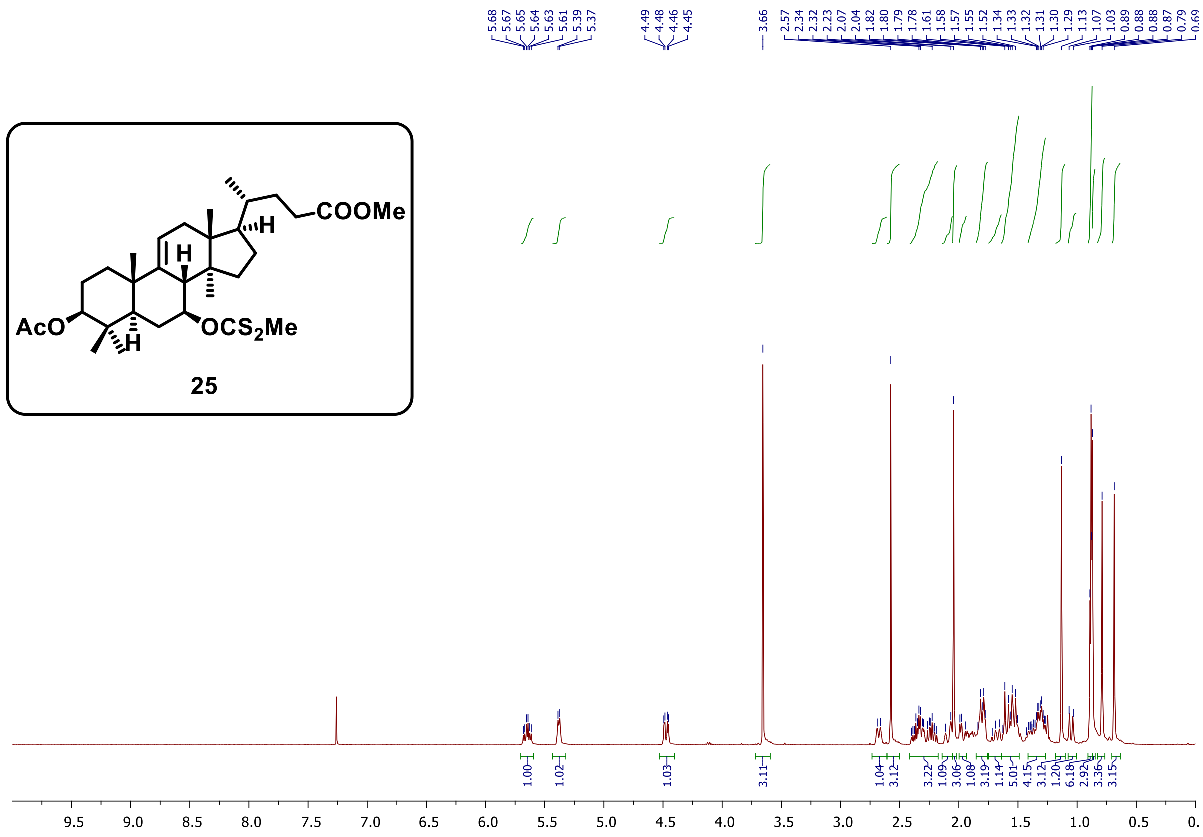

^{13}C NMR of **25**

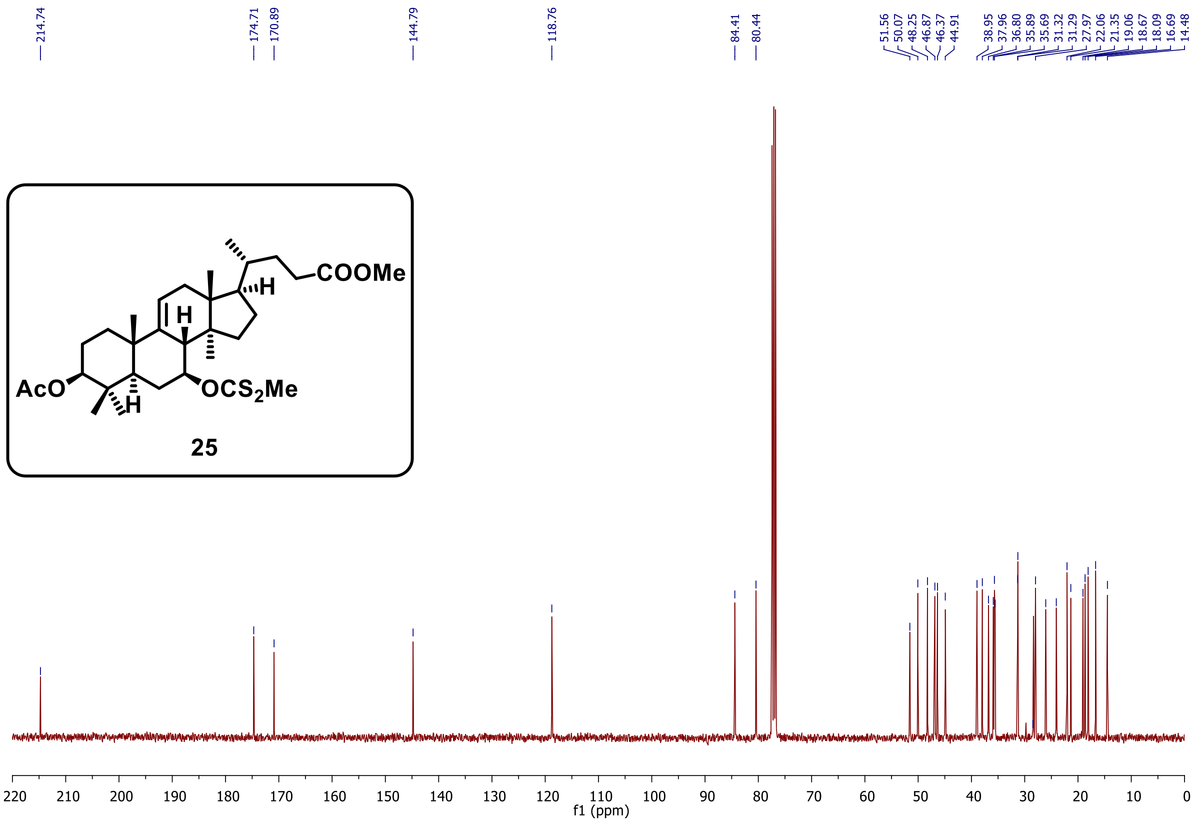

^{1}H NMR of **13**

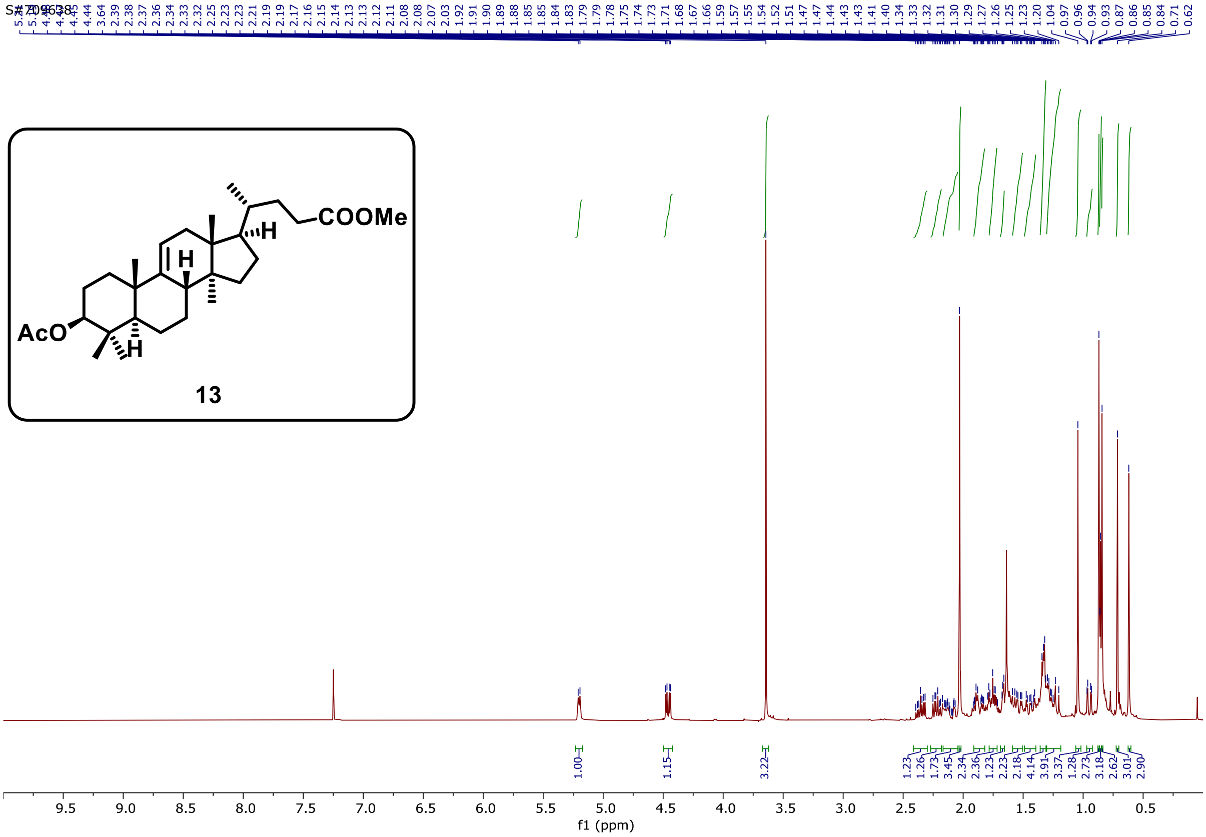

^{13}C NMR of **13**

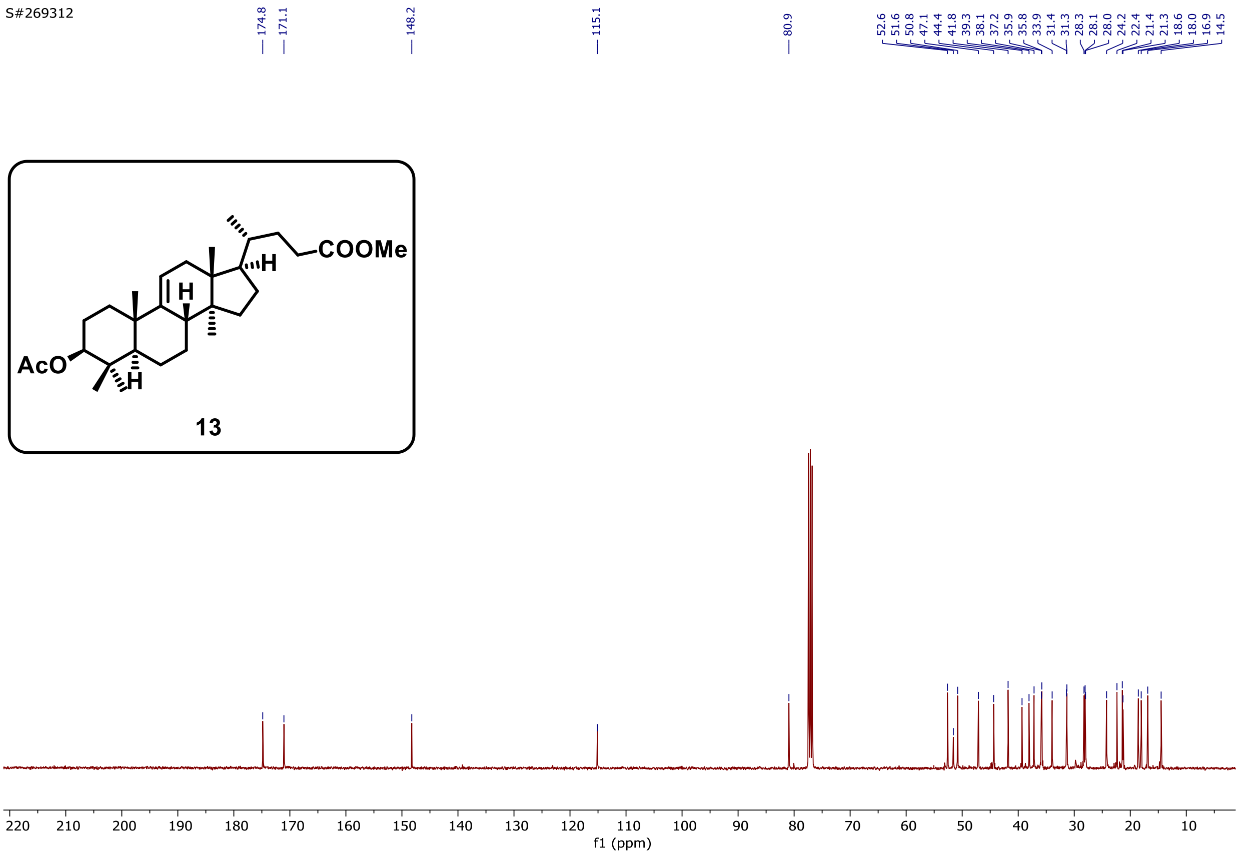

^{1}H NMR of **14**

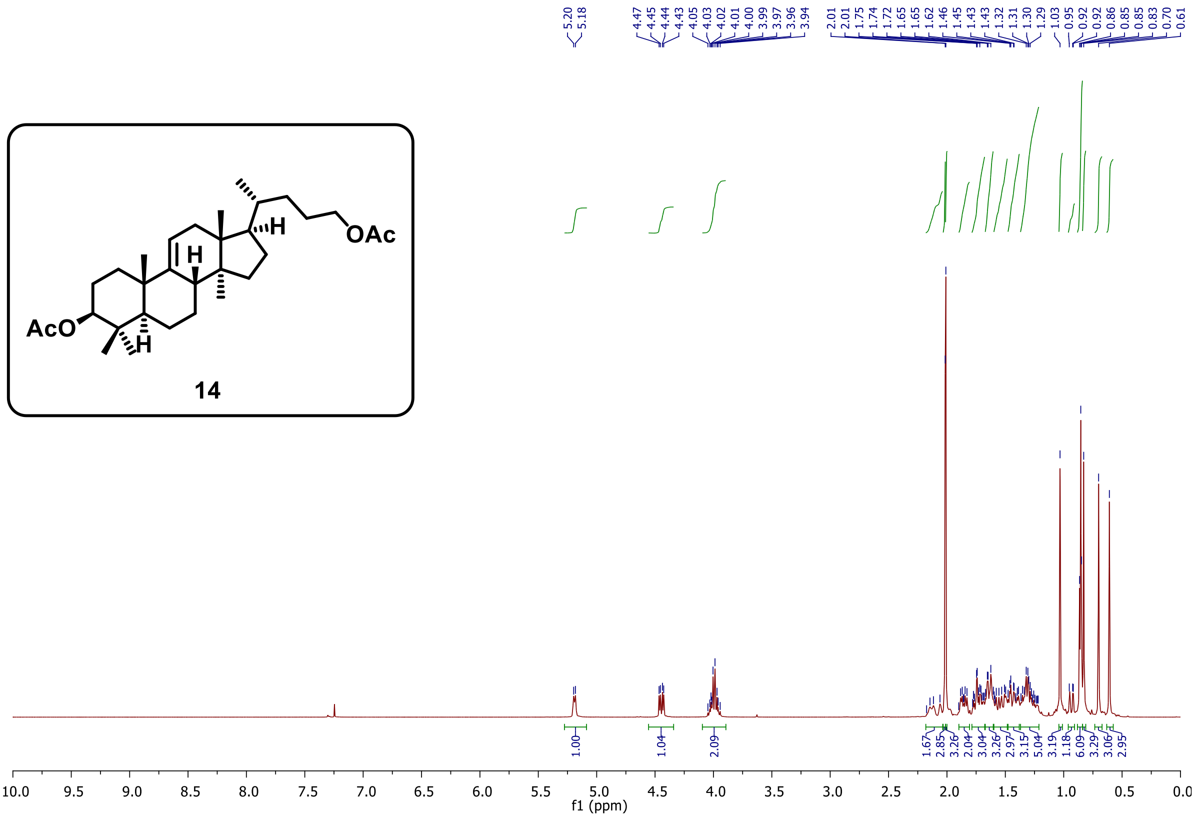

^{13}C NMR of **14**

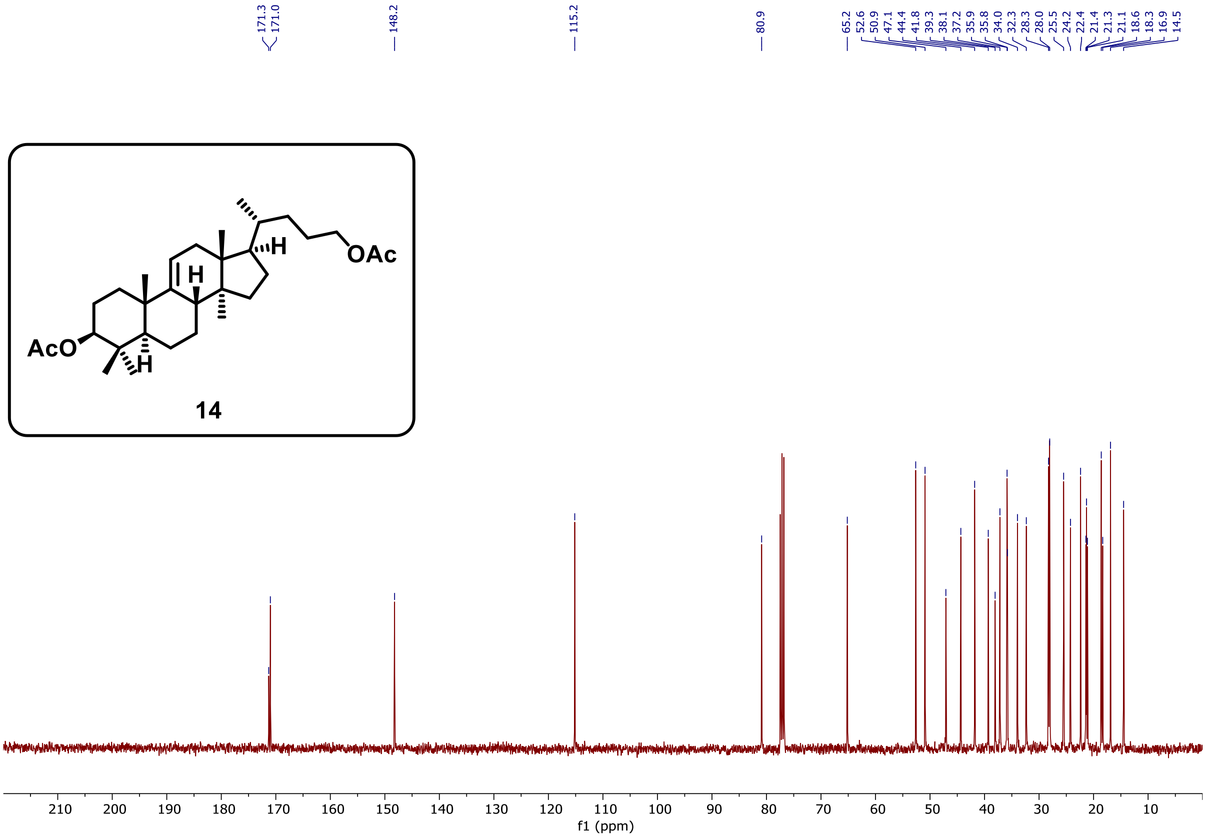

^{1}H NMR of **31**

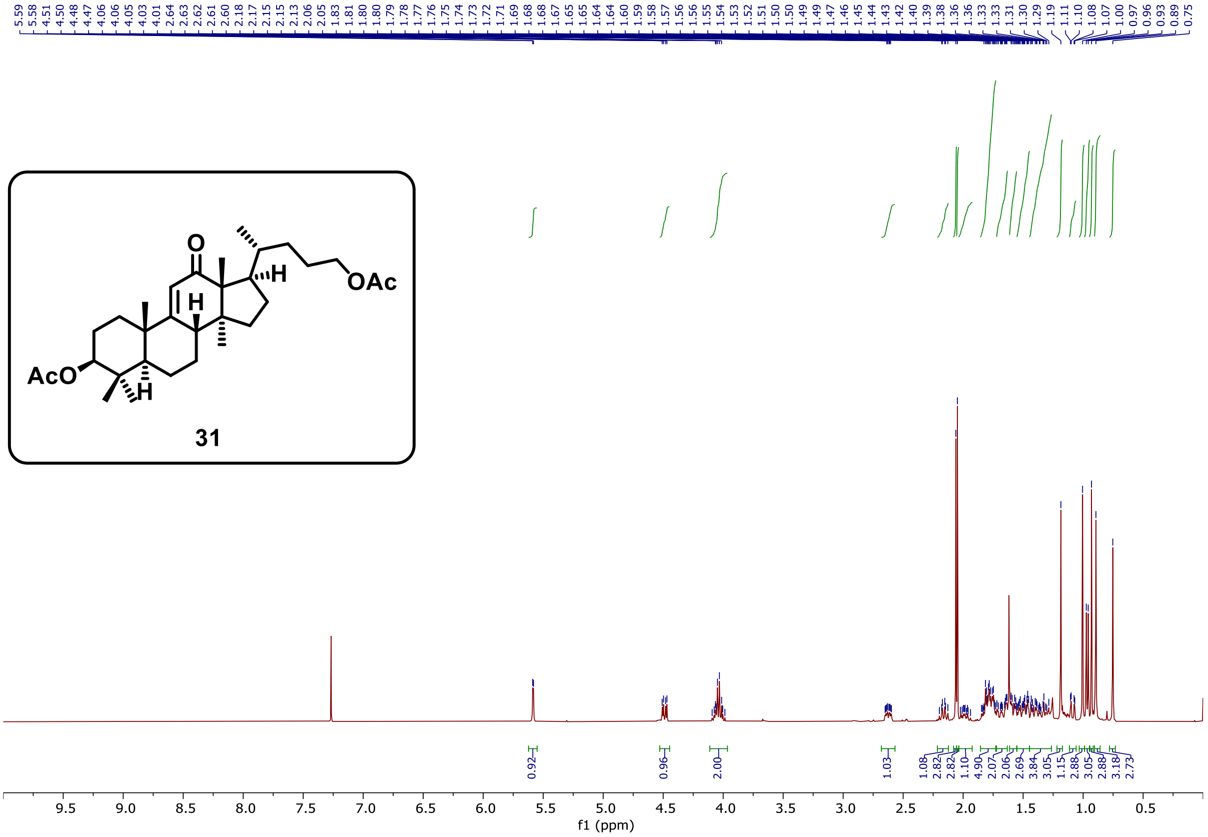

^{13}C NMR of **31**

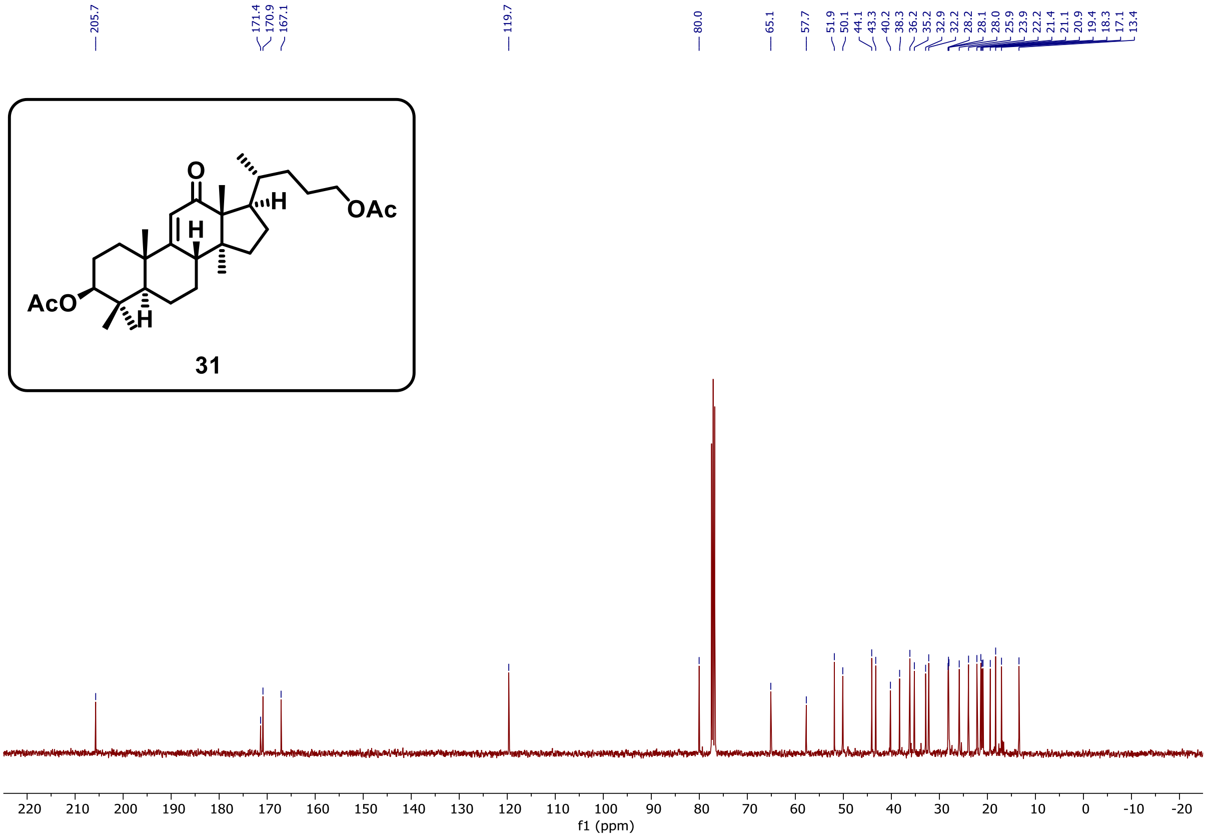

^{1}H NMR of **15**

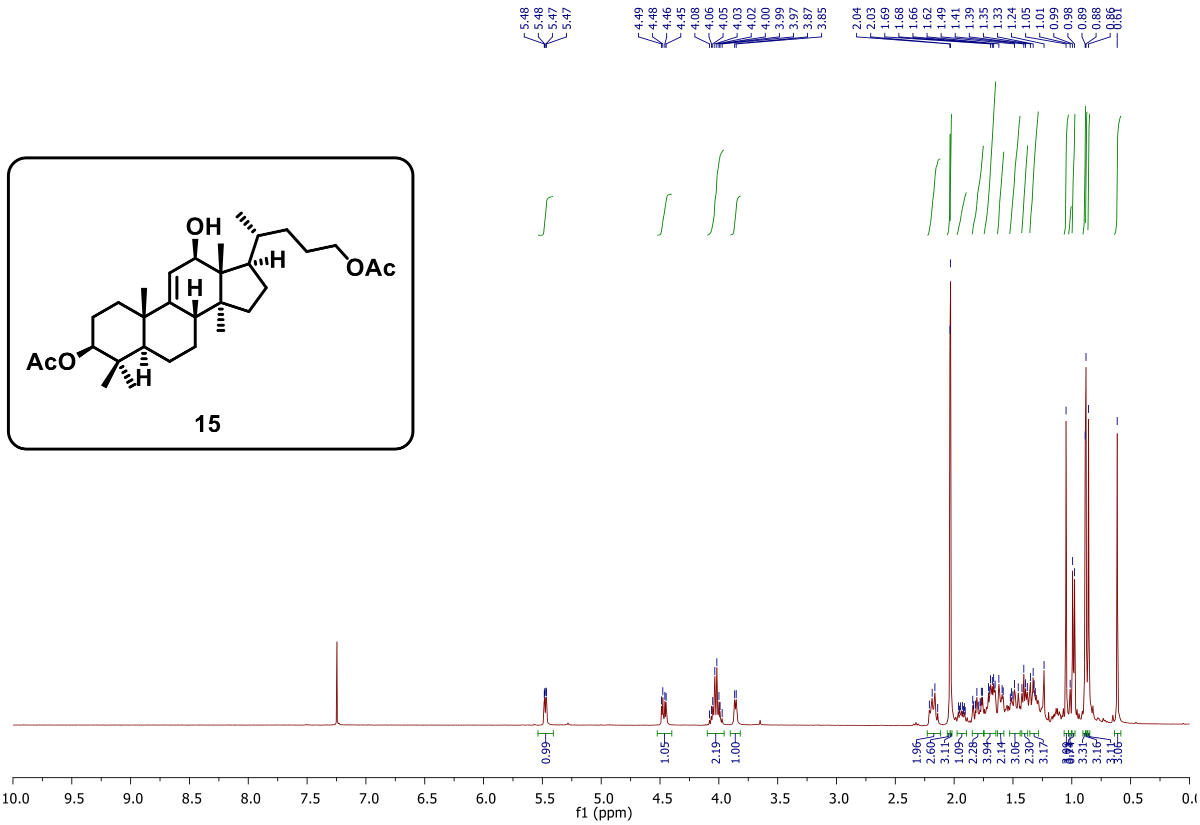

^{13}C NMR of **15**

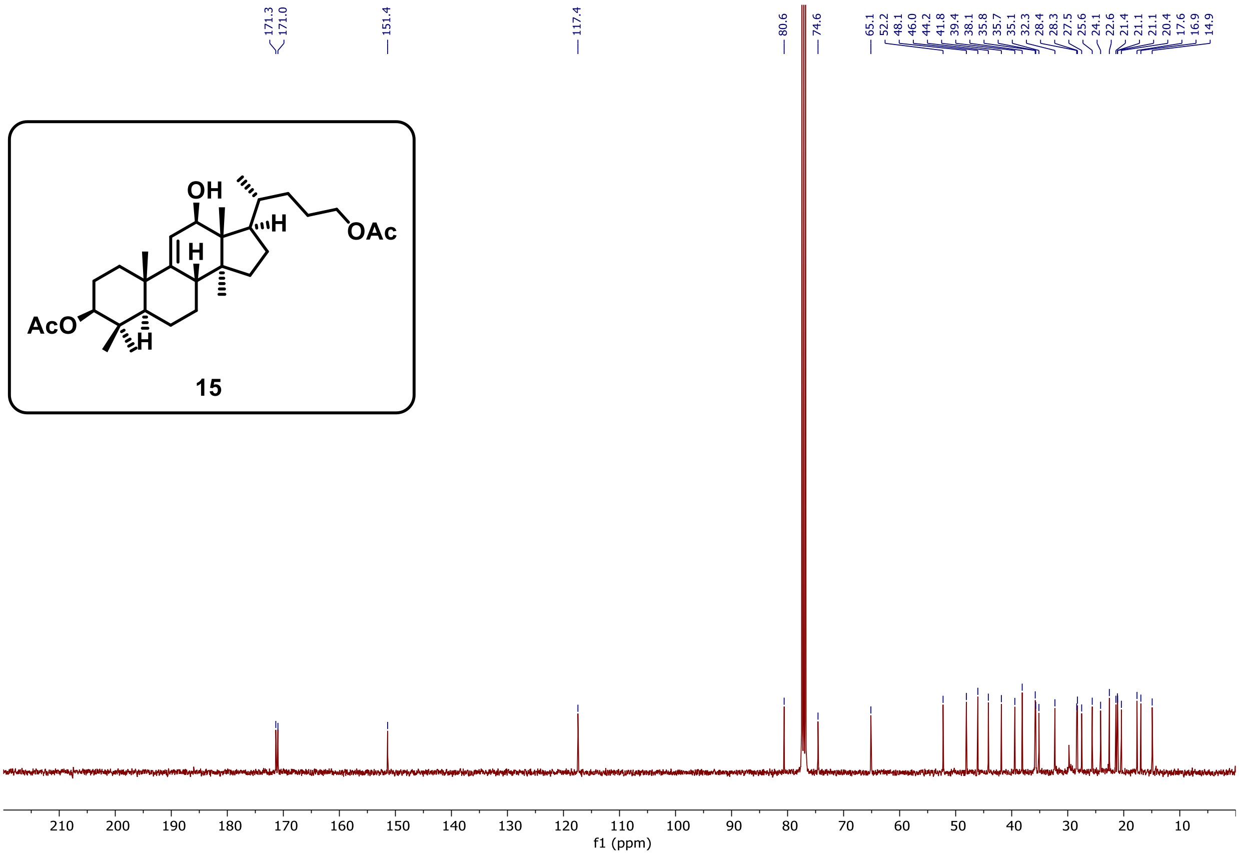

^{1}H NMR of **16**

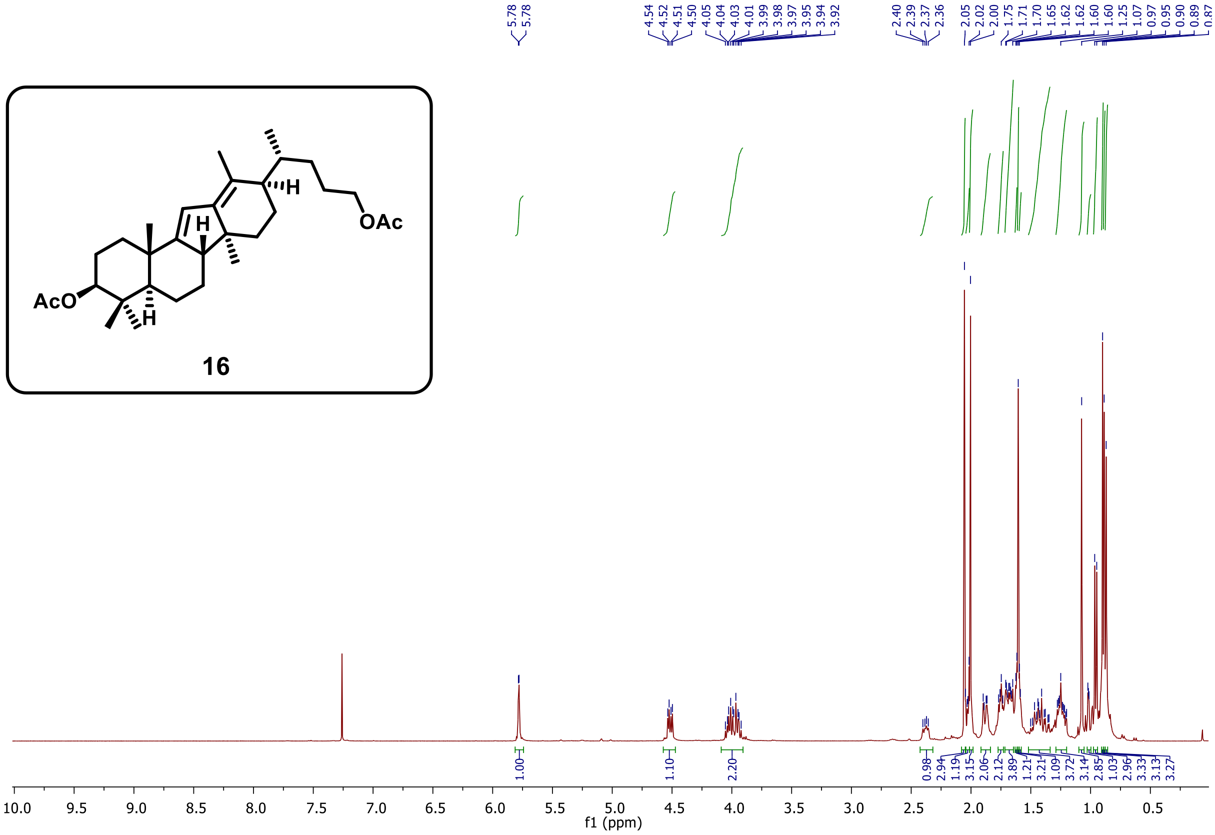

^{13}C NMR of **16**

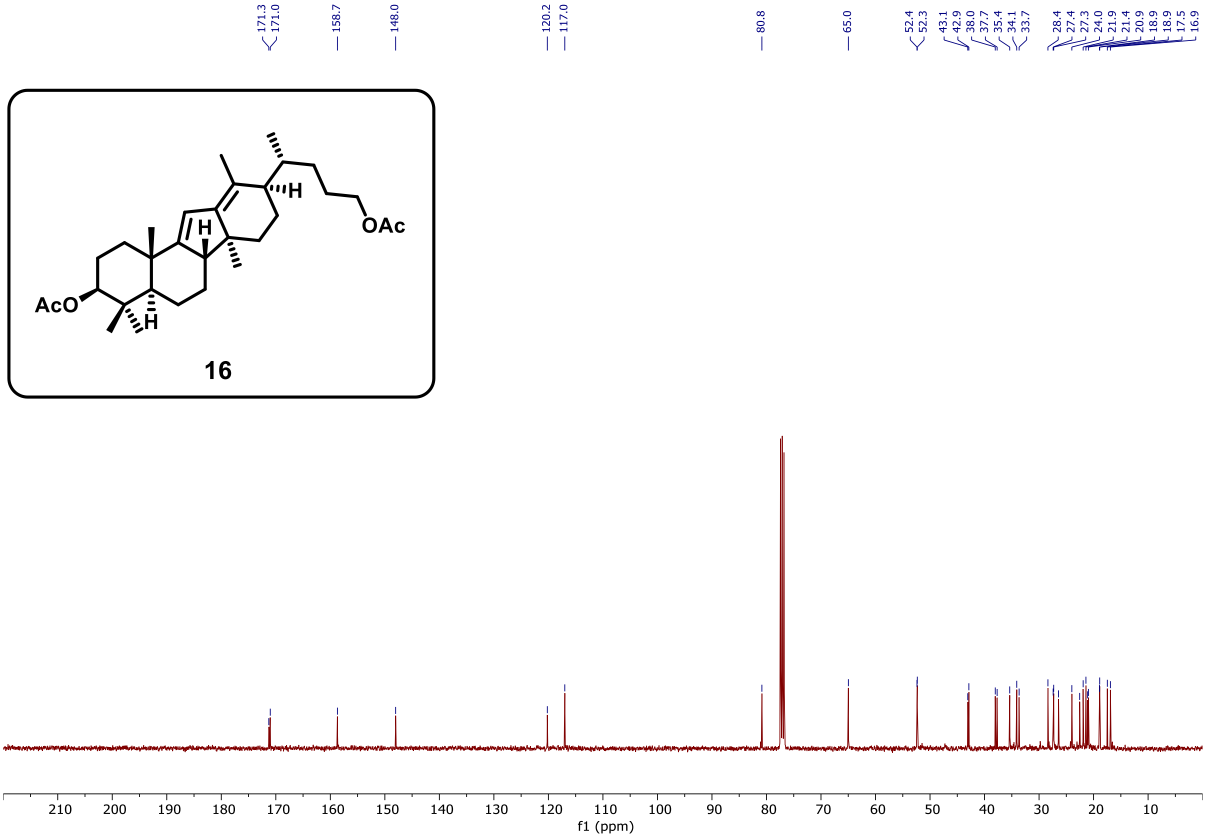

^{1}H NMR of **17**

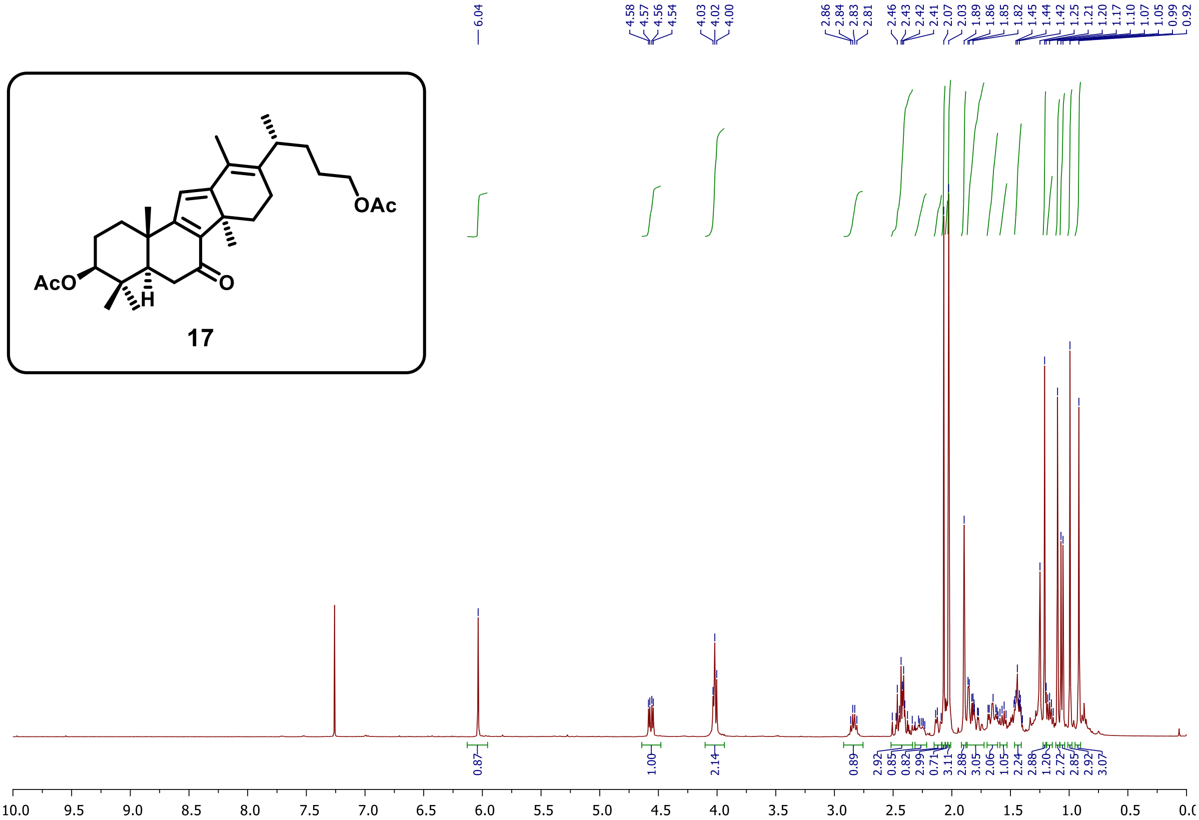

17

^{13}C NMR of **17**

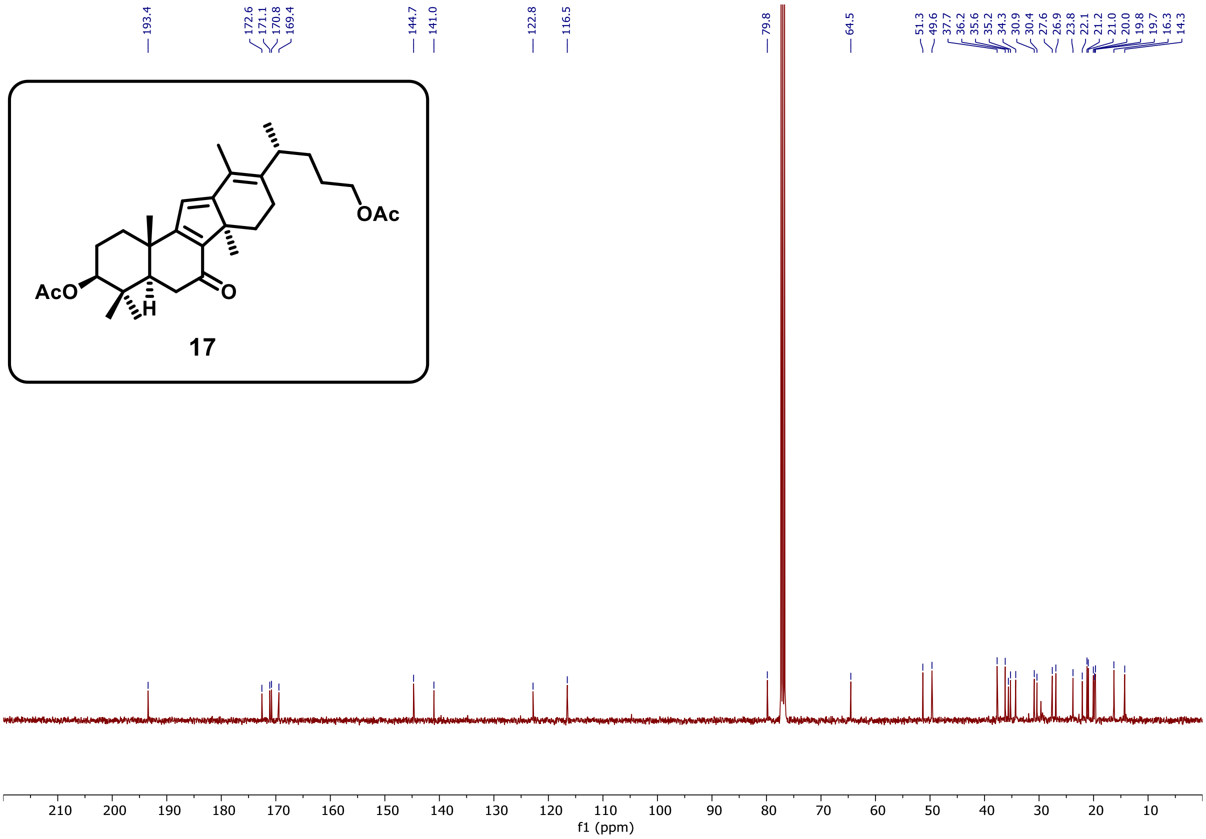

17

^{1}H NMR of **26**

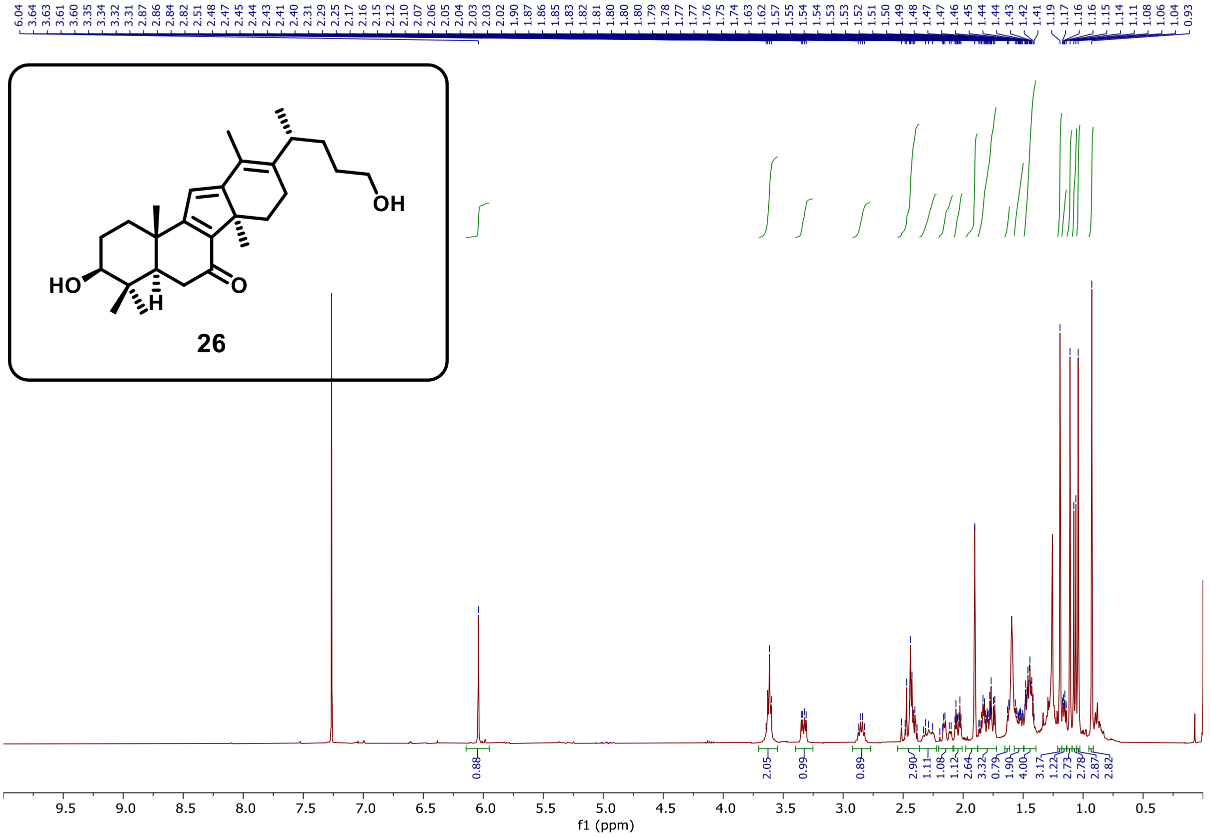

^{13}C NMR of **26**

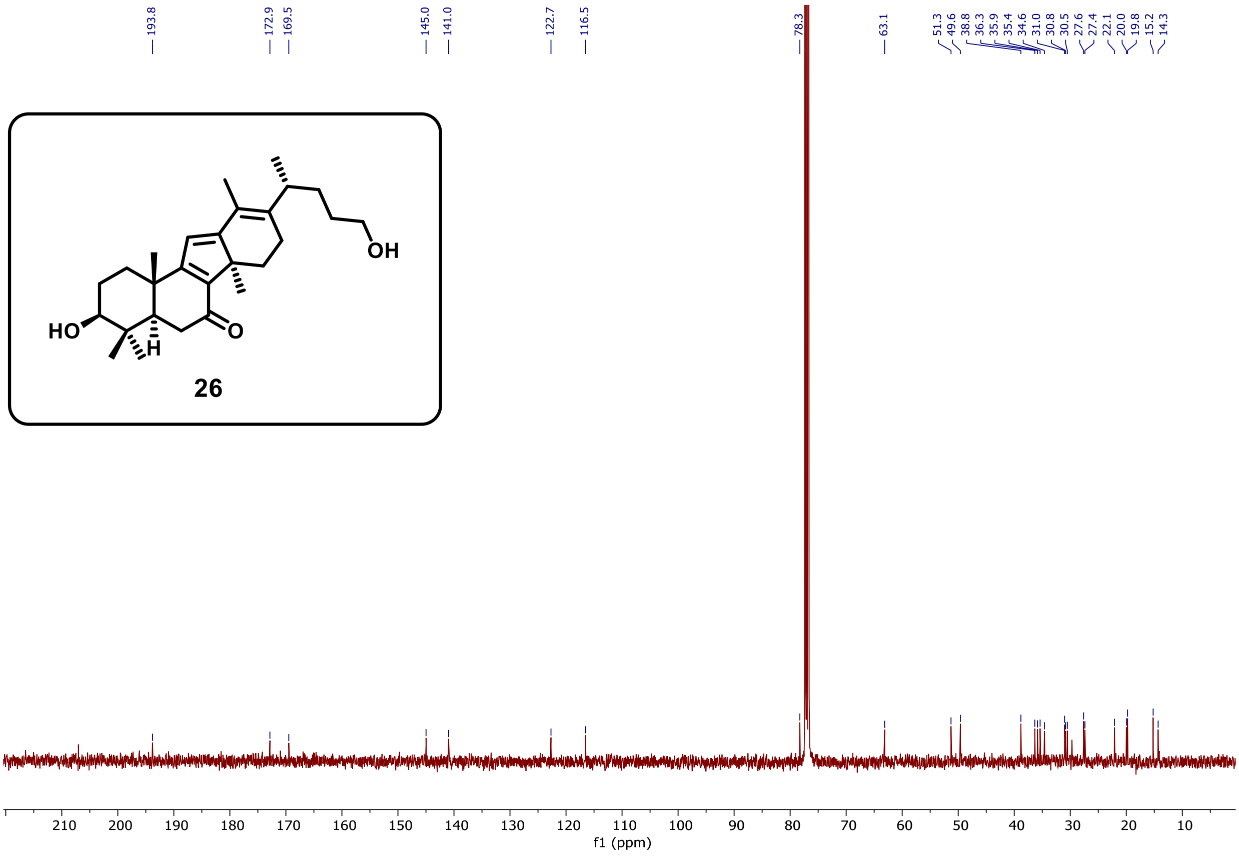

^{1}H NMR of **18**

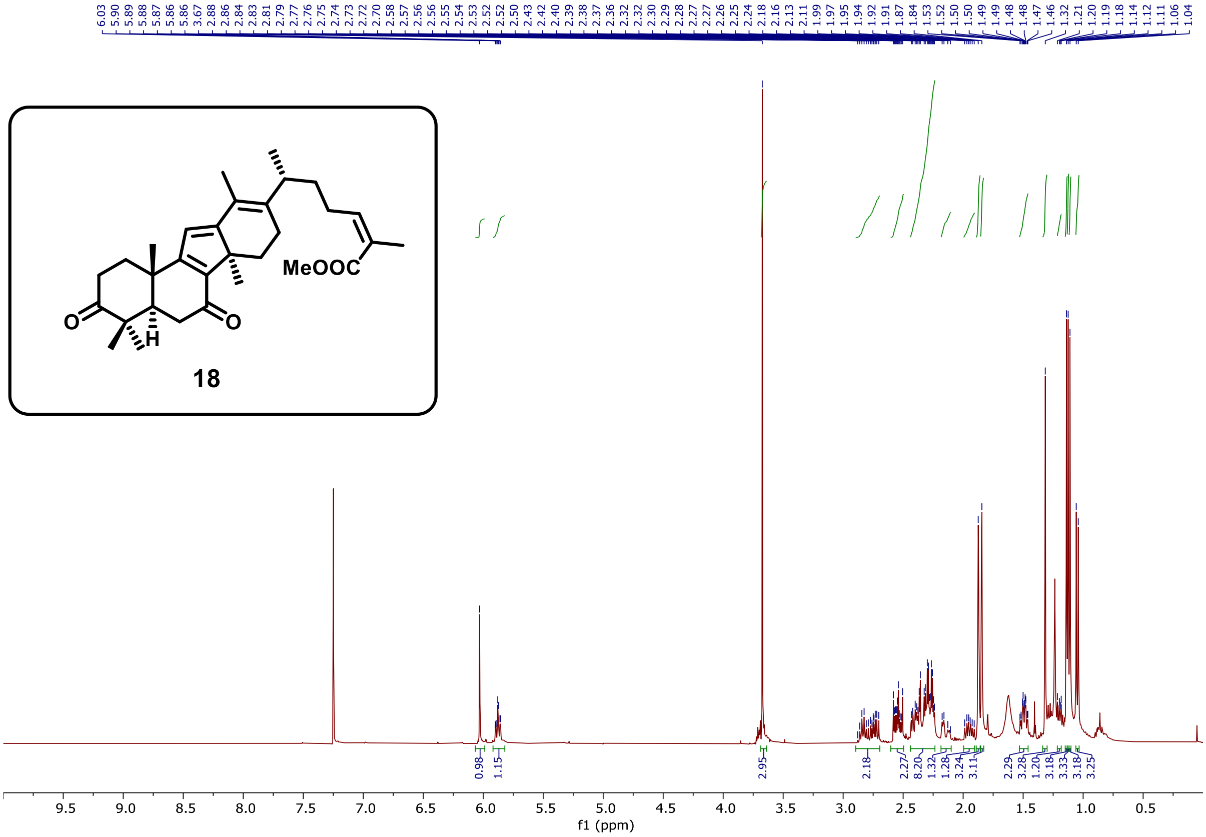

^{13}C NMR of **18**

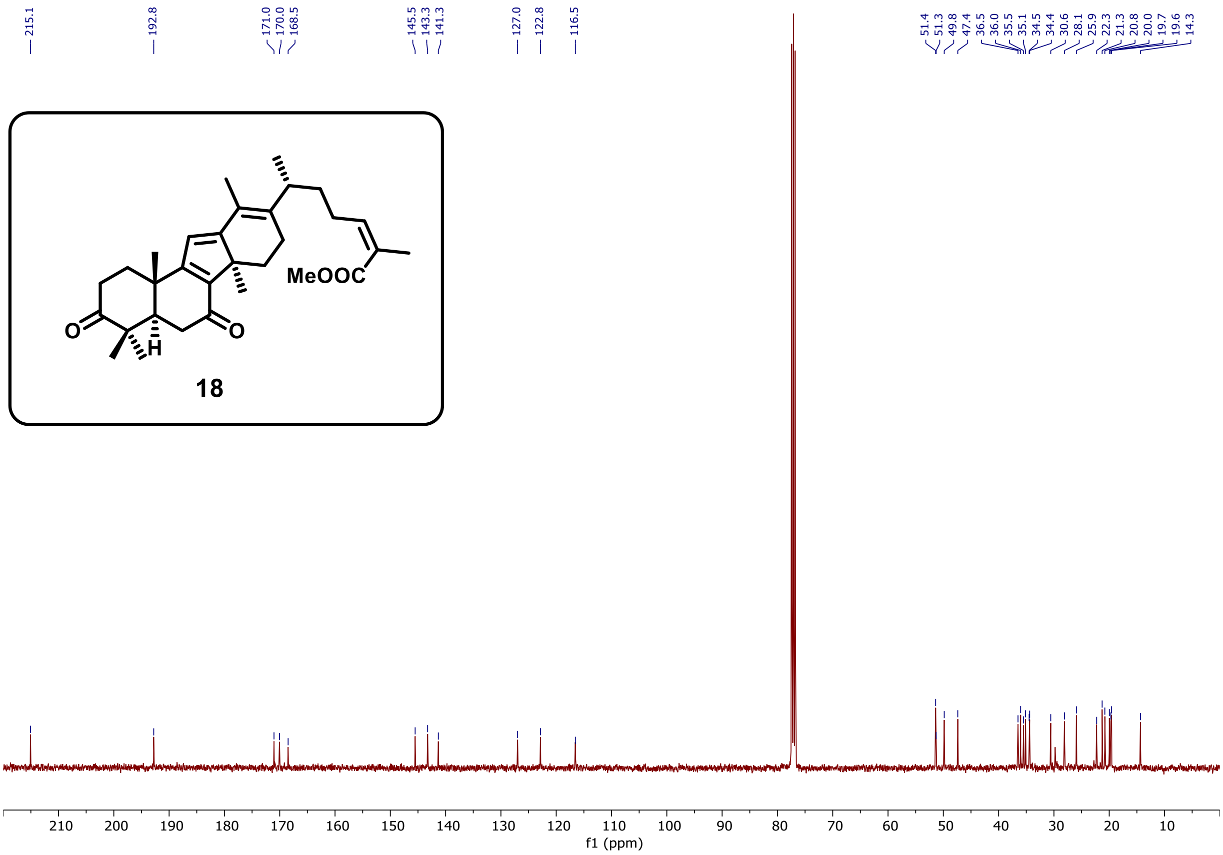

^{1}H NMR of **6**

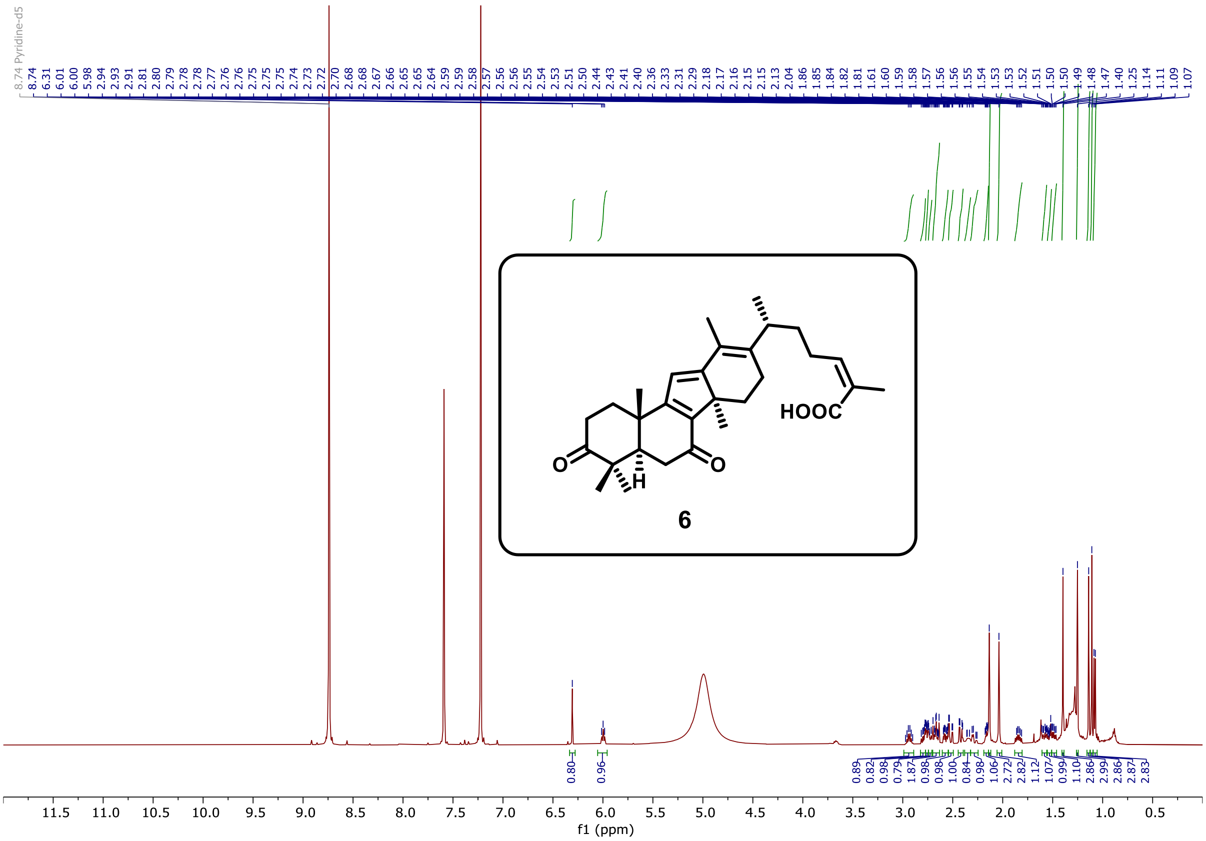

^{13}C NMR of **6**

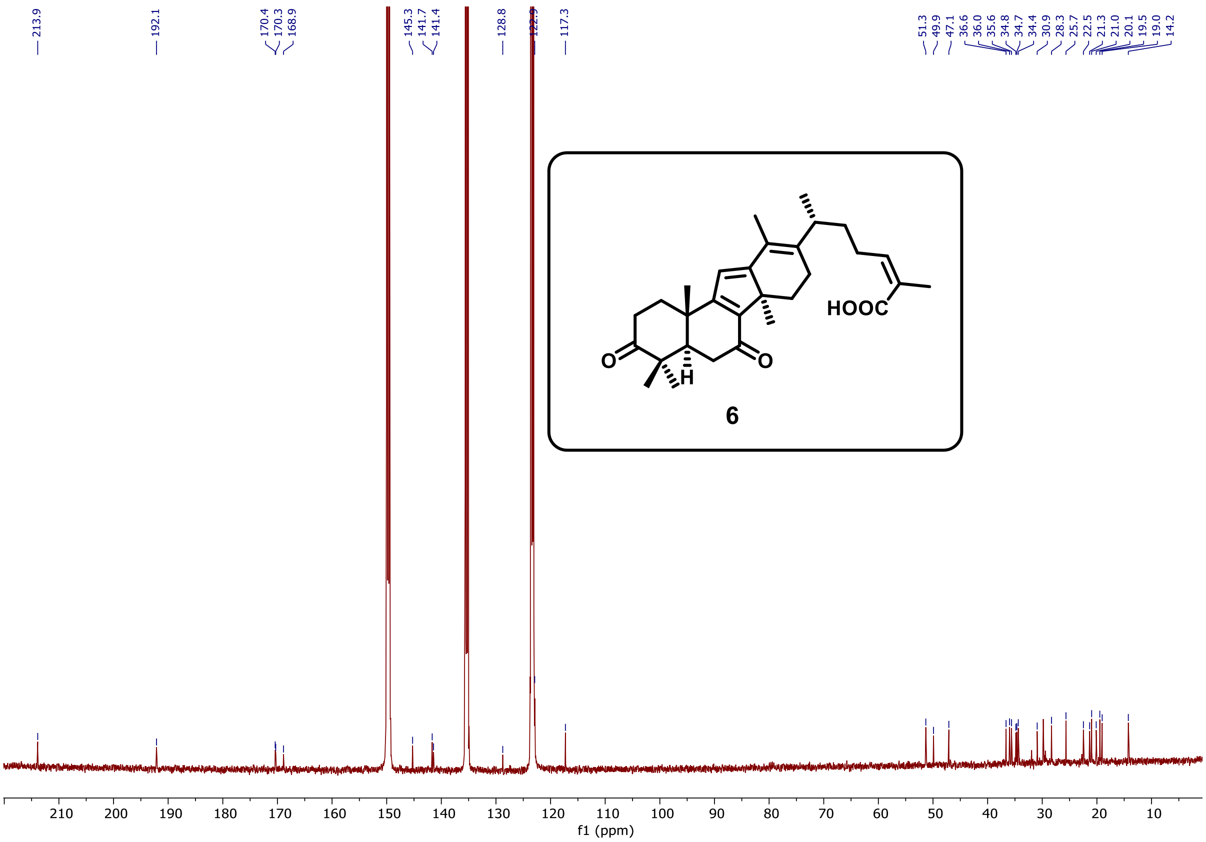

^{1}H NMR of **19**

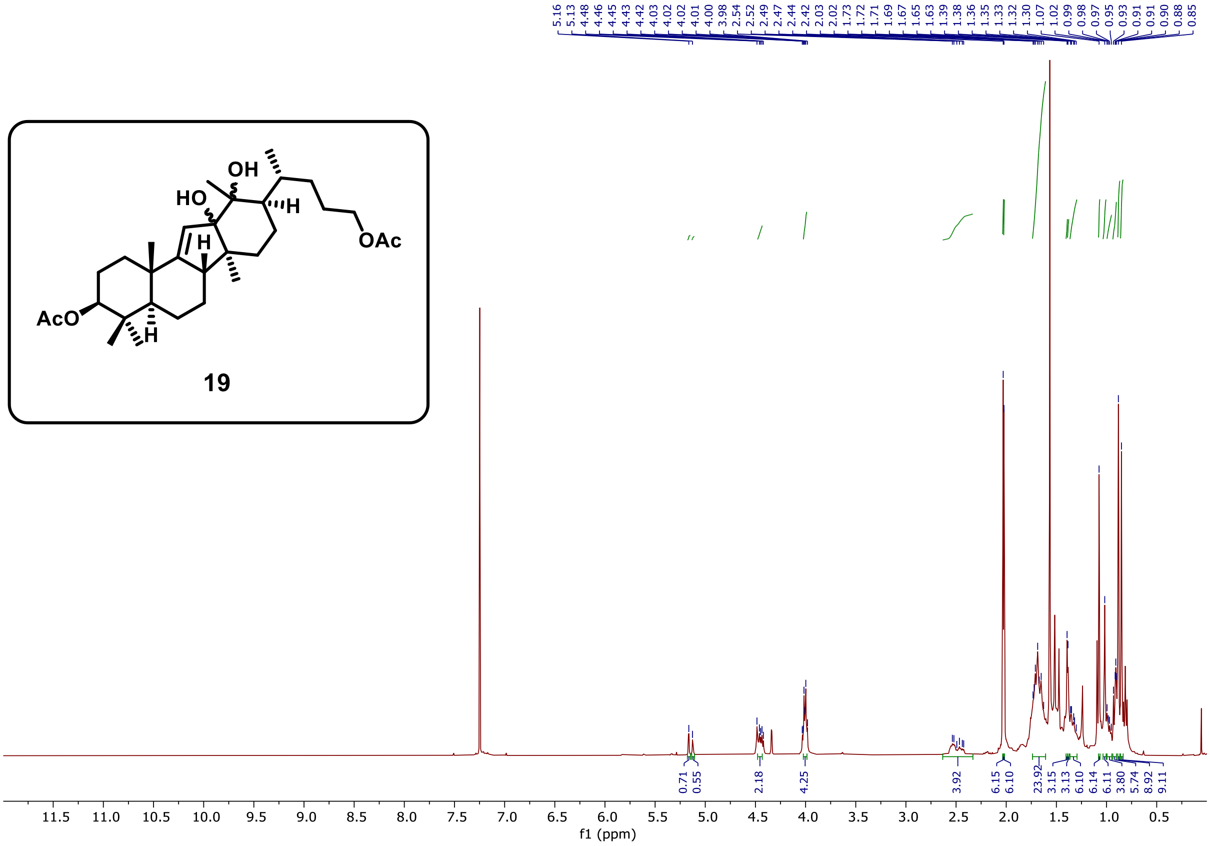

^{13}C NMR of **19**

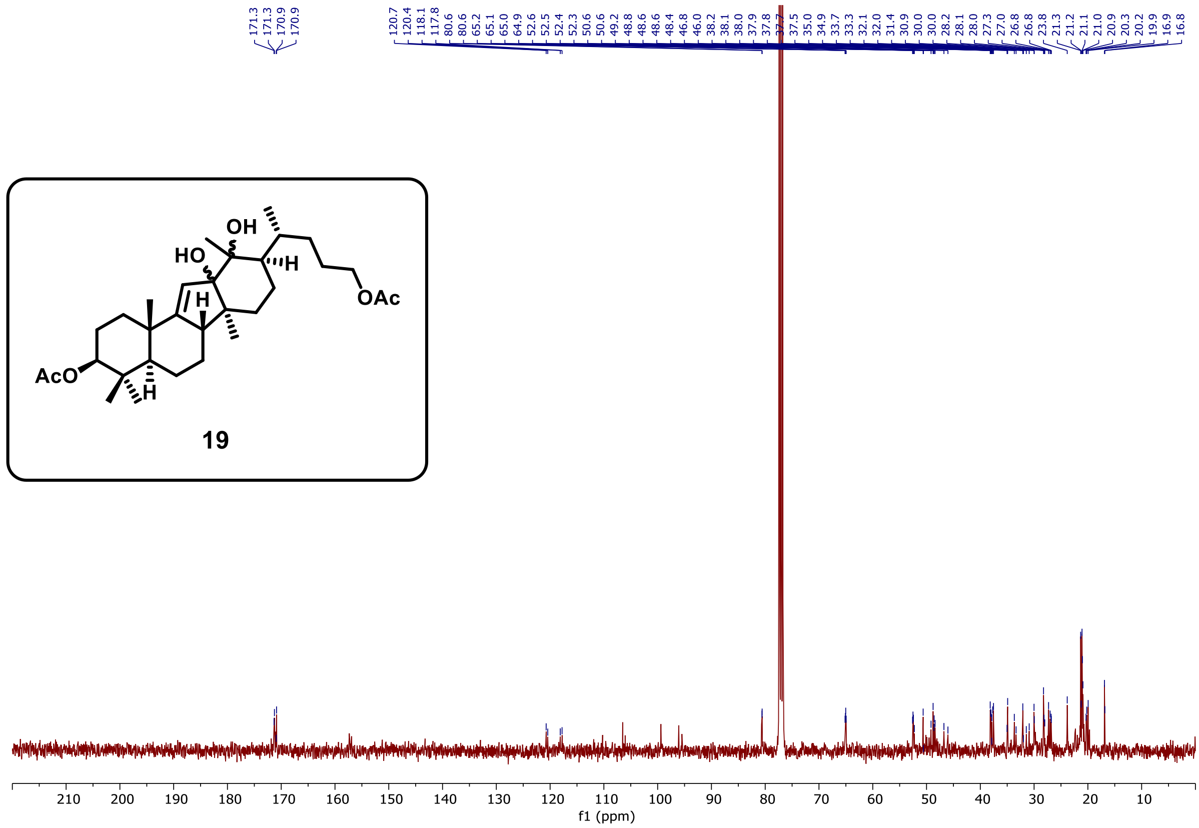

^{1}H NMR of **20**

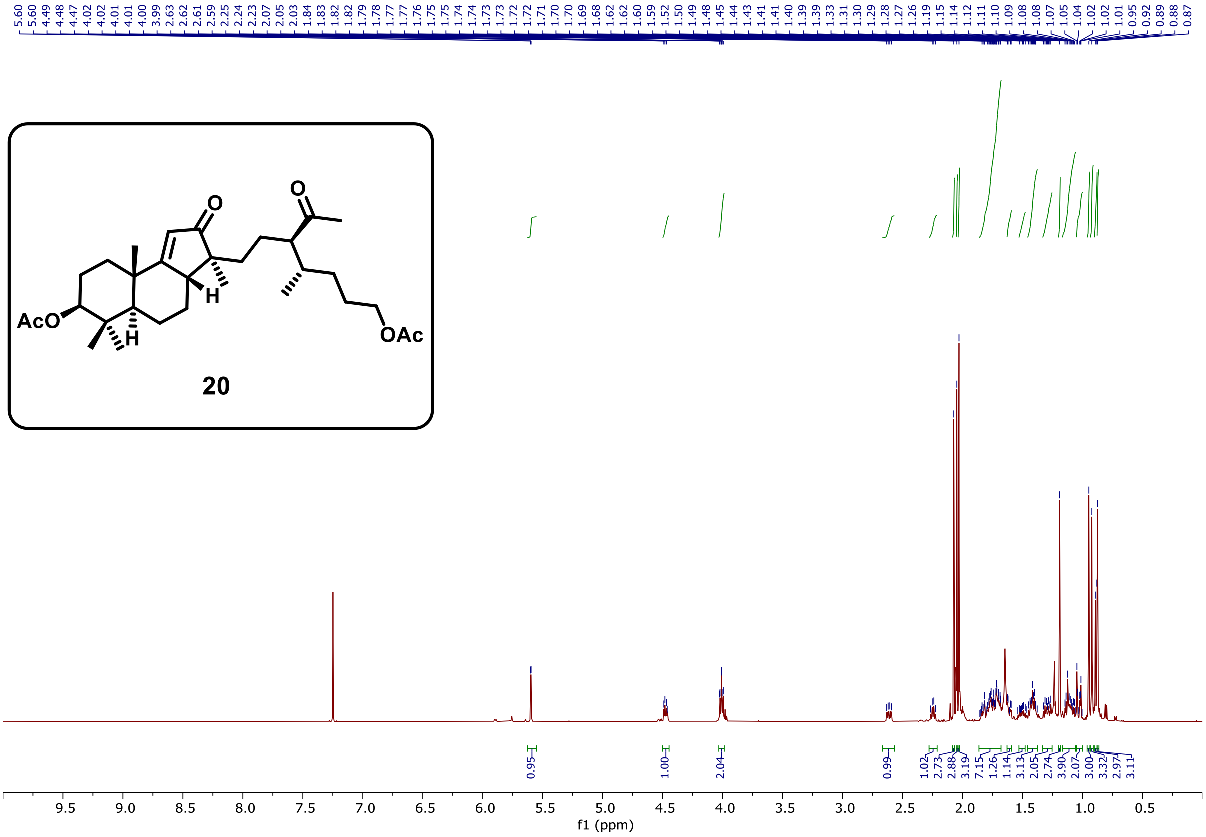

^{13}C NMR of **20**

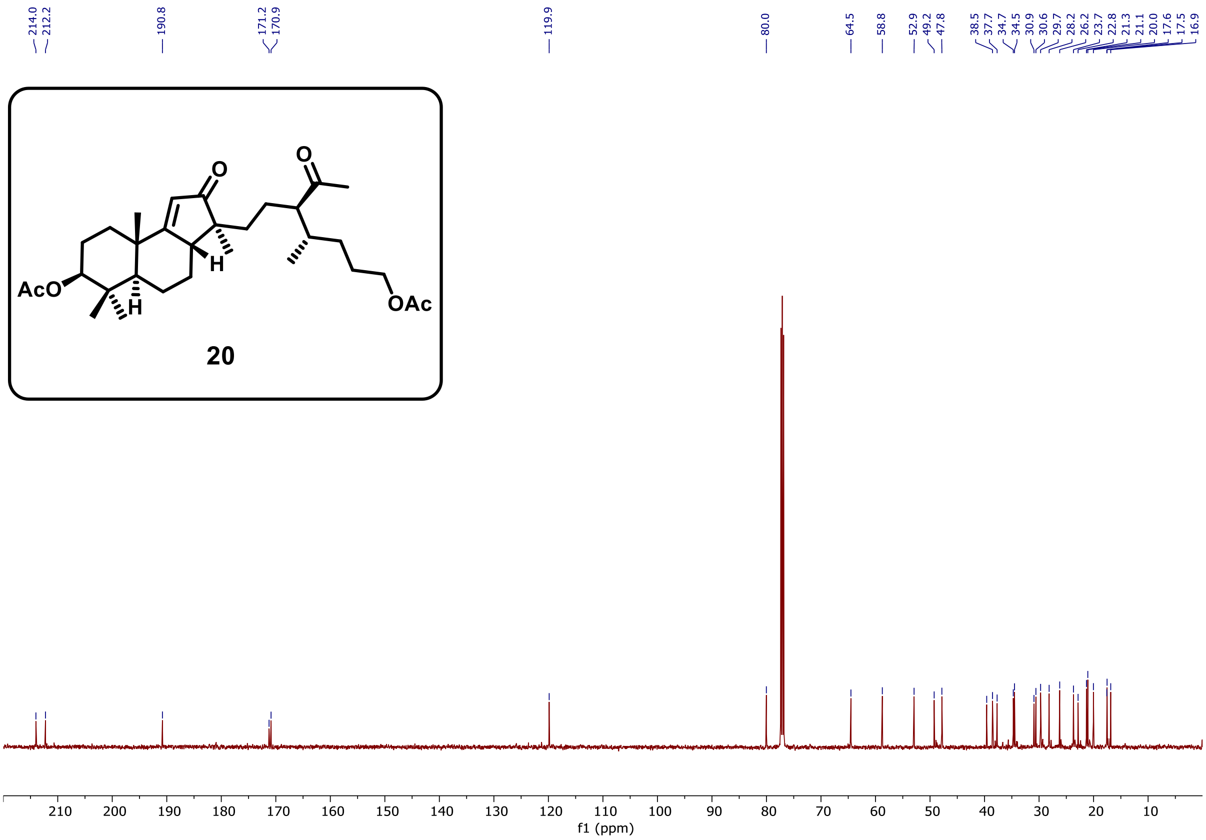

^{1}H NMR of **21**

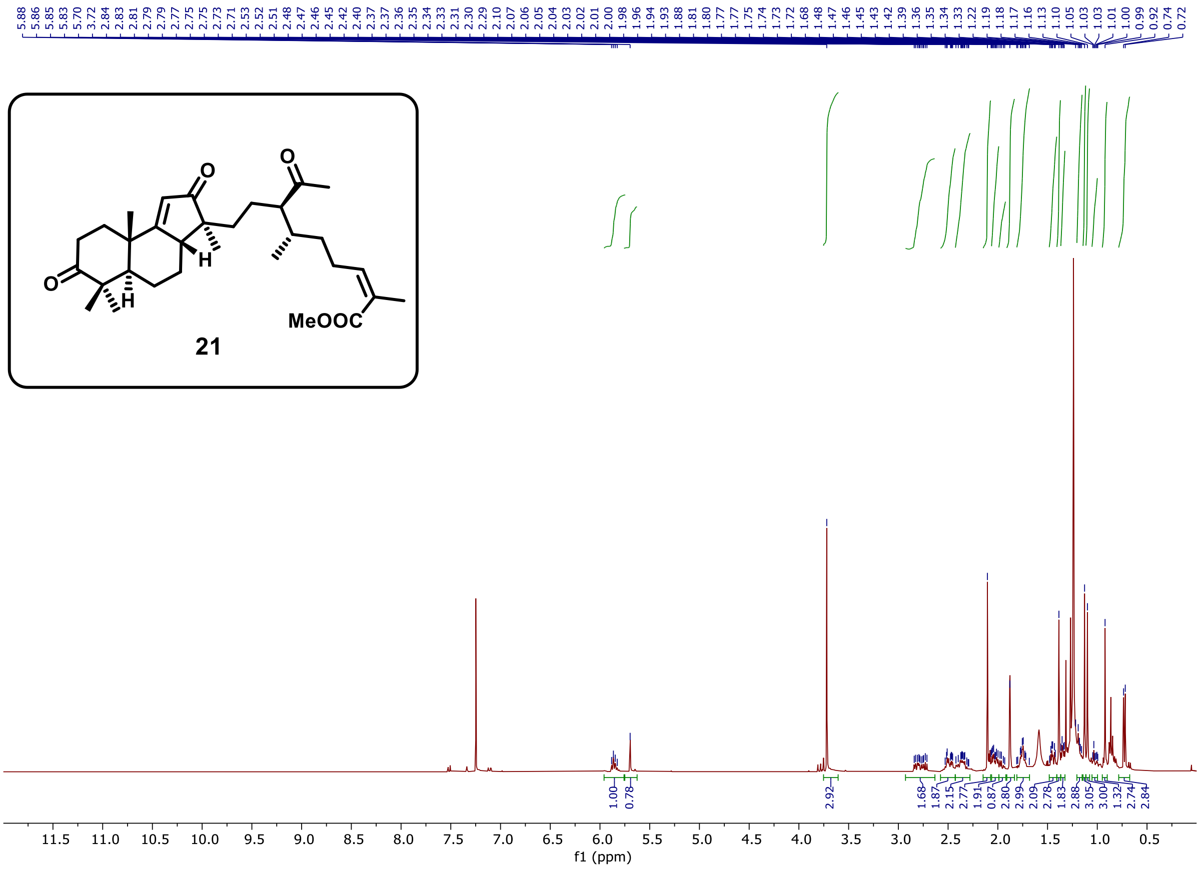

^{13}C NMR of **21**

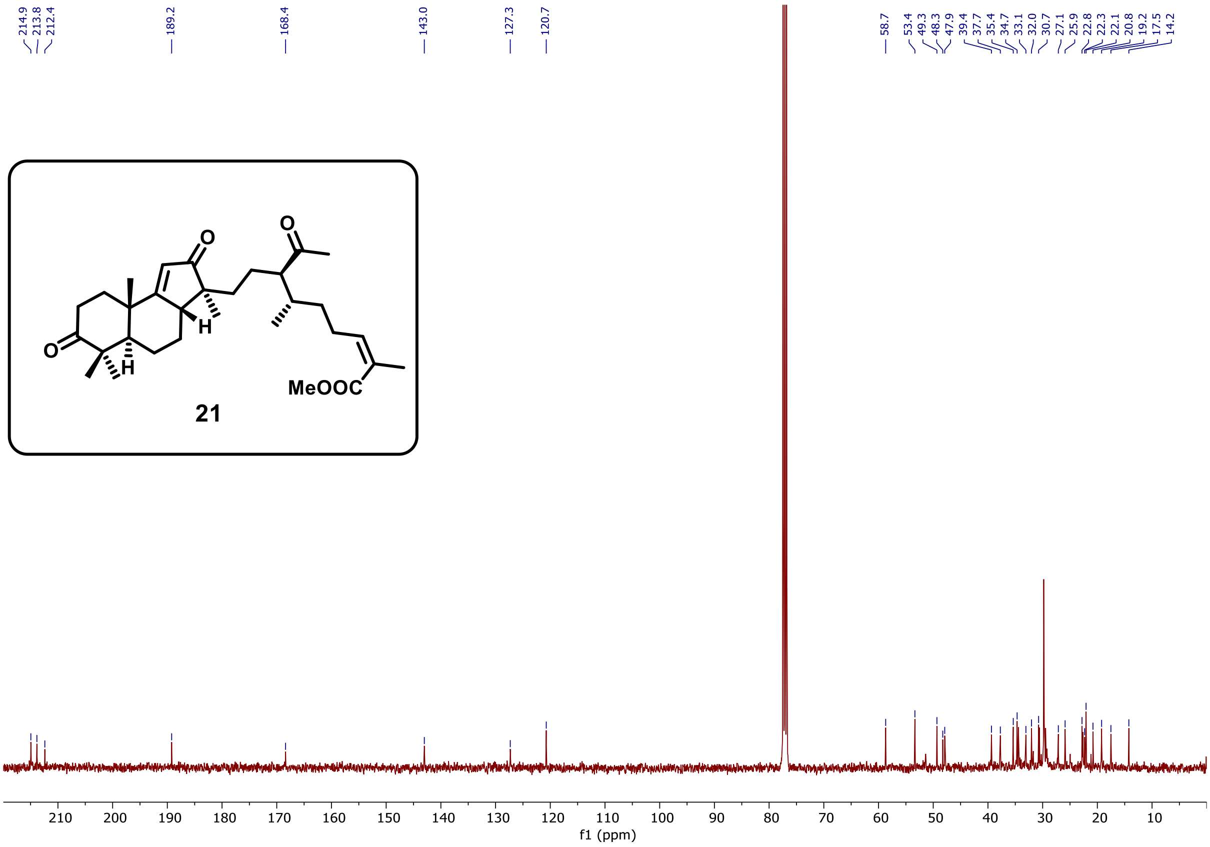

Supplement: SC-016-D5SC00669D-s001 [file SC-016-D5SC00669D-s001.pdf]
